# RASA2 deletion rescues immune synapse dysfunction, enhancing CAR T cell efficacy against DMGs

**DOI:** 10.1136/jitc-2025-013134

**Published:** 2026-03-30

**Authors:** Jorge Ibanez-Vega, Robert Teis, Jennifer K Ocasio, Peter Chockley, Aamir Ansari, Alejandro Allo Anido, Sanya Mehta, Michaela Meehl, Brooke Prinzing, Meghan Ward, David Odde, Suzanne Baker, Giedre Krenciute

**Affiliations:** 1 Bone Marrow Transplantation and Cellular Therapy, St Jude Children’s Research Hospital, Memphis, Tennessee, USA; 2 Developmental Neurobiology, St Jude Children’s Research Hospital, Memphis, Tennessee, USA; 3 Department of Chemical Engineering and Material Science, University of Minnesota, Minneapolis, Minnesota, USA; 4 Department of Biomedical Engineering, University of Minnesota, Minneapolis, Minnesota, USA; 5 Therapy Modeling and Design Center, University of Minnesota, Minneapolis, Minnesota, USA

**Keywords:** Chimeric antigen receptor - CAR, Immunotherapy, Central Nervous System Cancer

## Abstract

**Background:**

Chimeric antigen receptor (CAR) T-cell therapy has demonstrated safety and modest efficacy against diffuse midline gliomas (DMGs), a highly aggressive pediatric brain tumor. However, mechanisms of CAR T-cell resistance in DMG settings remain unknown.

**Methods:**

We compared the efficacy of B7-H3 CAR T-cells between SJ-DIPGX7c (DMG) and U87-MG (adult glioblastoma) patient-derived cell lines and showed impaired efficacy both in vitro and in vivo. We performed live-cell imaging and single-cell RNA sequencing to investigate deficiencies in immune synapse (IS) formation between CAR T-cells and DMGs. Lastly, we genetically deleted *RASA2*, a negative regulator of T cell activation, and evaluated the resulting impact on IS formation and quality, as well as in vitro and in vivo functionality.

**Results:**

We show that limited efficacy of B7-H3 CAR T-cells is due to DMG-mediated inefficient interaction between CAR T-cells and DMG cells. Specifically, DMG cells impair the IS formation, resulting in poor CAR T-cell activation, cytokine secretion, and limited anti-tumor response in vivo. RASA2 deletion improved CAR T-cell activation through the formation of a more functional IS. RASA2-deleted CAR T-cells exhibited enhanced calcium flux, increased accumulation of activated signaling molecules and lytic granules at the synapse, and increased actin cytoskeleton dynamics, which produced larger synaptic areas and resulted in enhanced migration ex vivo. Further, RASA2-deleted CAR T-cells demonstrated improved in vitro functionality and superior early in vivo anti-tumor responses against DMGs compared with controls.

**Conclusions:**

Our study highlights the importance of understanding tumor-specific factors that limit CAR T-cell response and using this information to design superior next-generation CAR T-cells. Specifically, we identify cytoskeleton remodeling and T cell motility as therapeutically actionable targets for future engineering approaches.

**WHAT IS ALREADY KNOWN ON THIS TOPIC**

- Chimeric antigen receptor (CAR) T cell interaction with tumor cells is imperfect due to a disorganized immune synapse when compared with T-cell receptor:Major histocompatibility complex (MHC) synaptic organization. However, this has not been studied in the context of brain tumors.

**WHAT THIS STUDY ADDS**

- For the first time, we characterize the immune synapse between diffuse midline glioma (DMG) and CAR T cells. We show that the synapse is dysfunctional, with defects that are more pronounced in DMGs and specific to this tumor type, as they are not observed in other pediatric brain tumors. The impaired immune synapse formation in CAR T cells limits their functionality and anti-DMG response, and deletion of RASA2 can improve these dysfunctions.

**HOW THIS STUDY MIGHT AFFECT RESEARCH, PRACTICE OR POLICY**

- Our study highlights that effective CAR T-cell and tumor-cell interactions are crucial for achieving optimal therapeutic outcomes in DMG, and that cytoskeletal remodeling and T-cell mobility are therapeutically actionable targets for future CAR T-cell engineering strategies.

## Background

 Diffuse midline gliomas (DMGs) are universally lethal cancers of the central nervous system that affect pediatric and adolescent patients. Surgical resection is impractical due to the high risk of damaging critical structures in the brain, including the brain stem. Current standard of care therapy is radiotherapy, which has only been able to extend overall survival by 3–4 months, while chemotherapy has not shown great efficacy.^1^ Thus, non-standard therapies, such as immunotherapy, arise as promising options. Indeed, GD2-targeting Chimeric Antigen Receptor (CAR) T-cells have demonstrated safety and encouraging clinical activity in altered histone 3 lysine to methionine (H3K27M) DMGs.^2^,^6^ Similarly, B7-H3-targeting CAR T-cells have shown safety and preliminary activity in early-phase trials.^5^ Despite encouraging early reports, tumor growth control by CAR T-cell treatment is limited, indicating the need for improved, next-generation CAR T-cells.

The initial response of CAR T-cells on tumor cell interaction depends on the assembly of a stable immune synapse (IS), a highly organized membranous structure that is formed at the T-cell/tumor-cell interface.^7^ The IS is produced by rapid polarization of the T-cell cytoplasm toward the synaptic membrane. The interface is characterized by the clustering of T-cell receptor (TCR) or CAR molecules^8^ along with accumulation of the actin cytoskeleton, the microtubule-organizing center (MTOC), lytic granules, and key signaling molecules such as zeta-chain-associated protein kinase (ZAP70) and Phospholipase C gamma (PLCγ).^9^ The quality of the IS, defined as the accumulation of actin cytoskeleton, signaling molecules, and lytic machinery to the synaptic membrane, directly correlates with CAR T-cell effector function and is predictive of in vivo performance.^10^ Consequently, improving the stability and quality of the IS represents a viable strategy for improving CAR T-cell therapeutic potential. While we have previously shown that synapse-tuned CARs have increased potency,^11^ the precise role of the IS in the anti-tumor efficacy of CAR T-cells against DMG remains uncharacterized.

Here, we provide a comprehensive characterization of the IS formed between CAR T-cells and DMG cells. We demonstrate that suboptimal T-cell and DMG-cell interactions impair CAR T-cell effector function. Using single-cell RNA sequencing (scRNA-seq), we show that DMG-engaged CAR T-cells are transcriptionally inert and downregulate the RAS signaling pathway. We demonstrate that deletion of RASA2, a negative regulator of the RAS pathway,^12^ in CAR T-cells facilitates the assembly of superior-quality IS and enhances antitumor responses both in vitro and in vivo against DMG models. We further show that RASA2 deletion improves the mobility and migration of CAR T cells, but that lack of persistence remains a challenge to overcome. Together, our findings show that optimal IS formation is critical for successful CAR T-cell response against DMG and identify RASA2 as a viable target for future engineering strategies.

## Results

### CAR T-cell response against DMGs is limited in vivo and in vitro

**Figure 1:**
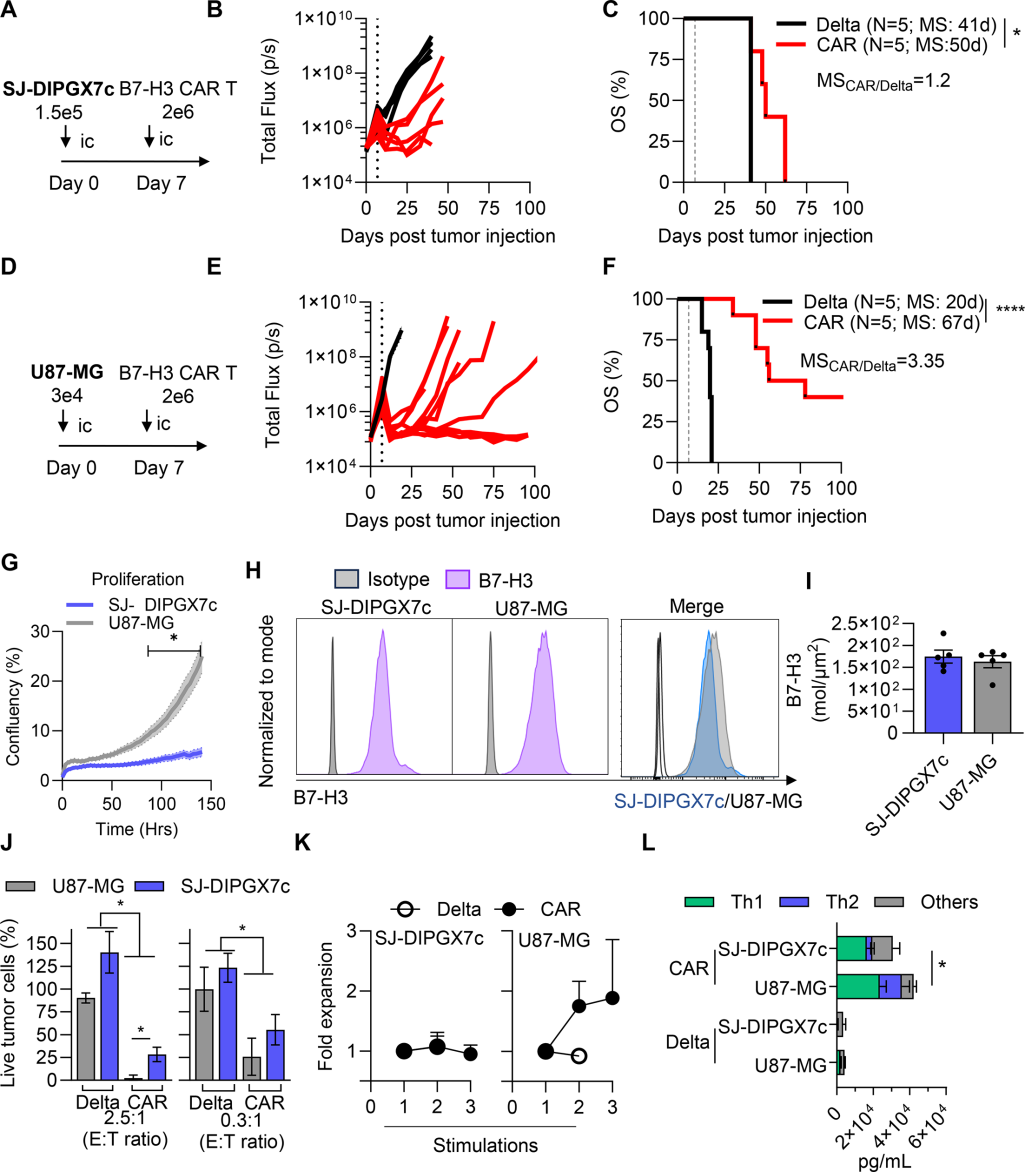
CAR T-cells have reduced effector functions in vitro and in vivo against DMGs in comparison to GBM. (**A**) Schematic of the in vivo experimental design for DMG intracranial implant model. SJ-DIPGX7c cells (1.5×105) were implanted intracranially (ic) into the brain cortex, followed by a single ic dose of 2×106 B7-H3- or Delta-CAR T-cells 7 days post-tumor injection. (**B**) Total flux from tumor cells in all mice treated with CAR T-cells. The tumors were measured weekly using bioluminescence imaging. (**C**) Kaplan-Meier survival analysis of mice treated with CAR T-cells (dashed line indicates T-cell infusion time) log rank (Mantel-Cox) test (N=5, *p<0.05. Median survival (MS) ratio=CAR MS divided by Delta MS). (**D**) Schematic of the in vivo experimental design for U87-MG intracranial implant model. U87-MG cells (3×104) were implanted intracranially (ic) into the brain cortex, followed by a single ic dose of 2×106 B7-H3- or Delta-CAR T-cells 7 days post-tumor injection. (**E**) Total flux from tumor cells in all mice treated with CAR T-cells. The tumors were measured weekly using bioluminescence imaging. (**F**) Kaplan-Meier survival analysis of mice treated with CAR T-cells (dashed line indicates T-cell infusion time) log rank (Mantel-Cox) test (N=5, ****p<0.0001. MS ratio=CAR MS divided by Delta MS). (**G**) Quantification of tumor cell (SJ-DIPGX7c and U87-MG) proliferation in vitro over time in mixed growth media (50% DIPG media and 50% U87-MG media), measured as percentage of confluency by Incucyte imaging (N=3, two-ways ANOVA with Sidak’s multiple comparison test, *p<0.05). (**H**) Representative histogram of B7-H3 expression in SJ-DIPGX7c and U87-MG, measured by flow cytometry. (**I**) B7-H3 molecules per cell quantification, normalized by their surface area (µm2) (antigen density) in SJ-DIPGX7c and U87-MG (N=4–7, technical repeats). Error bars represent mean and SEM. (**J**) MTS-based cytotoxicity assay of B7-H3-CAR T cells and Delta-CAR T-cells (control) against SJ-DIPGX7c and U87-MG tumor cells at high (2.5:1) and low (0.3:1) effector:target ratio (**E:T**). (N=3–4 T cell donors, Kruskal-Wallis test, Dunn’s multiple comparison test. *p<0.05). Error bars represent mean and SEM. (**K**) Serial stimulation assay using effector T-cells (B7-H3- or Delta-CAR T-cells) against U87-MG (N=6 T cell donors) and SJ-DIPGX7c (N=3–4 T Cell Donors) target cells at 2:1 E:T ratio. Fresh target cells were added every 7 days. Error bars represent mean and SEM. (**L**) Summary plots of cytokine production by B7-H3-CAR T-cells or Delta-CAR T-cells in the supernatant when cultured with U87-MG and SJ-DIPGX7c cells at 2:1 E:T ratio after 24 hours of stimulation. Cytokines were measured using MILLIPLEX cytokine assay (N=5–7, one-way ANOVA, Fisher’s LSD multiple comparison test, *p<0.05). ANOVA, analysis of variance; DMGs, diffuse midline gliomas; GBM, glioblastoma; OS, overall survival. Error bars represent mean and SEM.

CAR T-cells have shown promising results in early-phase clinical trials against DMG tumors but failed to achieve a complete response.^4^ To investigate the mechanisms driving CAR T-cell resistance in DMGs, we first compared the in vivo anti-tumor efficacy of second-generation B7-H3-specific CAR T-cells^13^ (CAR, (online supplemental figure S1) compared with interacting but non-signaling B7-H3-Delta CAR T-cells (Delta-CAR, (online supplemental figure S1) in an adult glioblastoma model (U87-MG) vs a pediatric DMG model (SJ-DIPGX7c) (figure 1A, B, D and E). We found that B7-H3-specific CAR T-cells extended the median overall survival of U87-MG-bearing mice by 3.35-fold compared with Delta-CAR T-cells, whereas only 1.2-fold improvement was shown for SJ-DIPGX7c model (figure 1C,F). We acknowledge that the established in vivo tumor models used in this experiment differ, requiring different tumor cell numbers (SJ-DIPGX7c>>U87 MG) to be injected for successful tumor implantation. Thus, a potential explanation for the difference in anti-tumor response is that B7-H3-CAR T-cells were simply unable to eliminate the larger tumor burden. However, while fewer cells were implanted, U87-MG tumors grew more aggressively in vivo, as reflected by the shorter median overall survival of control (Delta-CAR) treated mice (20 days vs 41 days, respectively). Consistent with this observation, our in vitro assays demonstrated that U87-MG cells indeed proliferate significantly faster than SJ-DIPGX7c cells (figure 1G). Yet, B7-H3-CAR T cells were more effective against the faster-growing U87-MG tumors. We next asked whether differences in B7-H3 antigen density might account for this differential CAR anti-tumor response. However, both cell lines express equivalent levels of B7-H3, as determined by flow cytometry and absolute antigen quantification (figure 1H,I). Thus, antigen density also does not account for the observed difference in anti-tumor response. Together, this suggests that DMG-specific CAR T cell resistance mechanisms may be involved.

Given the significant difference in tumor control of B7-H3 CAR T-cells against SJ-DIPGX7c and U87-MG in vivo, we next evaluated whether the three main components of CAR T-cell functionality (cytotoxicity, expansion, and cytokine secretion) were reduced in SJ-DIPGX7c compared with U87-MG in vitro. We first comprehensively evaluated the cytotoxicity of B7-H3 CAR T cells against U87-MG and SJ-DIPGX7c using standard MTS cytotoxicity assays, IncuCyte time-resolved killing assays, and single-cell apoptosis imaging. First, we performed co-cultures at high (2.5:1) and low (0.3:1) effector to target (E:T) ratios and measured tumor cell viability 24 hours later by MTS. Our results show that CAR T cells were more effective at killing U87-MG cells than SJ-DIPGX7c at both high and low E:T ratios (figure 1J).

We further characterized killing kinetics using IncuCyte automated live-cell imaging at a low E:T ratio (0.25:1). Our results indicate that CAR T-cells eradicated U87-MG cells faster than SJ-DIPGX7c cells, measured by the reduction in total tumor area over time (online supplemental figure S2A). This kinetic difference was corroborated at the single-cell level via live cell spinning disc confocal microscopy (online supplemental figure S2B). To quantify the dynamics of individual interactions, CAR T-cells were labeled with CellTrace Violet and the calcium indicator Cal-520 AM, while tumor cells were labeled with CellTracker Red CMPTX and the membrane impermeable viability dye DRAQ7. By using calcium flux as a proxy for the beginning of T-cell and tumor-cell interaction and DRAQ7 internalization to mark the onset of apoptosis, we determined that the mean time to kill a single U87-MG cell was approximately 3 hours, whereas ~7 hours were needed for SJ-DIPGX7c (online supplemental figure S2C and D). Overall, our results demonstrate that CAR T-cells are less effective and less efficient at killing SJ-DIPGX7c cells compared with U87-MG cells.

We next evaluated whether T-cell expansion was impaired following exposure to SJ-DIPGX7c compared with U87-MG. We used a repeated stimulation assay in which CAR T-cells were re-challenged with fresh tumor cells at 2:1 E:T ratio every 7 days until the T-cells failed to clear the tumor or expand. At the end of each stimulation, we measured the cumulative CAR T-cell expansion. Over two rounds of stimulation, CAR T-cells expanded 1.57 times more when co-cultured with U87-MG than with SJ-DIPGX7c (figure 1K). Notably, CAR T-cells co-cultured with U87-MG expanded beyond Delta-CAR T-cells levels, whereas with SJ-DIPGX7c CAR T-cells were indistinguishable from Delta-CAR T-cells.

Finally, we assessed the cytokines secreted by CAR T-cells after 24 hours of initial co-culture (2:1 E:T ratio) using Milliplex human cytokine multiplex assay. Overall, CAR T-cells exposed to SJ-DIPGX7c secreted significantly lower amounts of cytokines in comparison to U87-MG cells (figure 1L). This difference was primarily driven by higher Th1 (IFNγ, TNFα, granulocyte–macrophage colony-stimulating factor (GM-CSF), and IL-2) and Th2 (IL-4, IL-5, IL-10, and IL-13) response in U87-MG, with significant differences in IL-5 and IL-13 (online supplemental figure S3).

Altogether, our findings demonstrate that B7-H3-specific CAR T-cell effector function is significantly impaired on exposure to SJ-DIPGX7c tumors in vitro and in vivo compared with U87-MG, even though U87-MG cells grow more aggressively and B7-H3 antigen density on the surface of both cell lines is equivalent.

### IS is severely impaired in CAR T-cells interacting with DMGs

**Figure 2:**
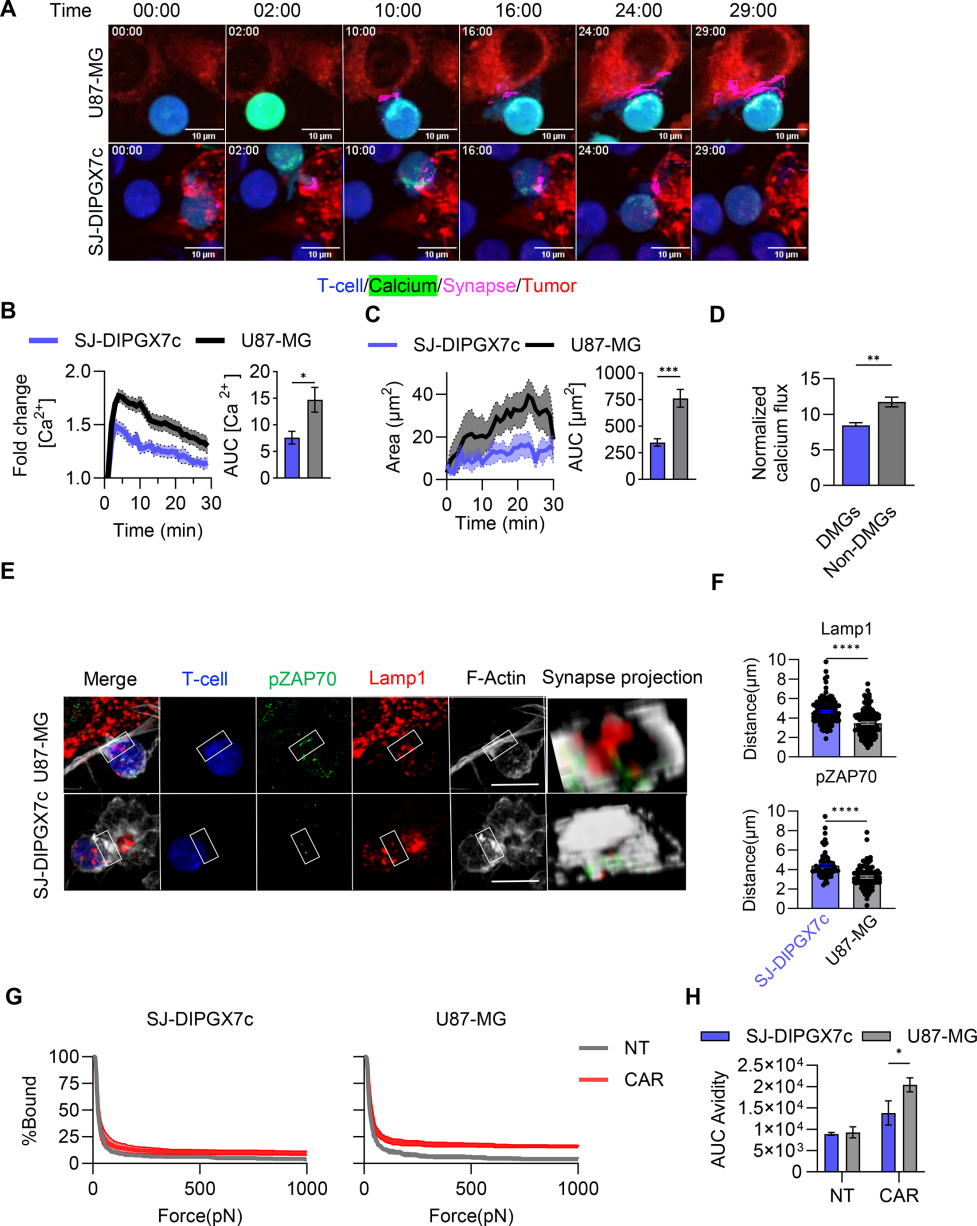
DMGs impair CAR T-cell immune synapse formation. (**A**) Representative confocal live cell time-lapse images of CAR T-cells interacting with SJ-DIPGX7c and U87-MG. CAR T-cells were labeled with CellTrace violet (Blue) and CAL520AM (Green) for calcium flux, and tumor cells were labeled with CellTracker Red-CMTPX (Red). The synapse was automatically generated as the intersection between T-cell and tumor cell labeling (Magenta) (mean Z-projection is shown scale bar=10 µm). (**B, C**) Quantification of calcium flux and synaptic size in CAR T-cells on tumor cell interaction shown in (**A**), respectively (cells=35-90, N= 2 T cell donors, Unpaired t-test, *p=0.0023, ***p=0.0005). Error bars represent mean and SEM. (**D**) Normalized calcium flux by B7-H3 density per cell, comparing non-DMG (GBM:U87-MG, MB:D425, HDMBO3, DAOY, EPN: ST1, ST2, and HGG:HGG42) and DMG (SJ-DIPGX7c, SJ-DIPGX9c, SJ-DIPGX29c, SJ-DIPGX37c, HSJD-DIPG007) interacting CAR T-cells (cells=6–90, N=2 T cell donors, Unpaired t-test, **p=0.0032). Error bars represent mean and SEM. (**E**) Representative confocal images of CAR T-cells (blue) and tumor cells (SJ-DIPGX7c and U87-MG) after 30 min of co-culture. pZAP70 (green), Lysosomes (Lamp1) (Red), and F-actin (white), merge, and synapse projection are shown (single Z-plane is shown scale bar=10 µm). (**F**) Quantification of the accumulation of Lytic granules (Lamp1) and pZAP70 at the immune synapse, measured as the distance of the center of mass of the labeling towards the synapse (smaller values indicate increased accumulation) (N=3 T cell donors, cells=63–134, unpaired t-test. ***p=0.0003, ****p<0.0001). Error bars represent mean and SEM. (**G**) Single cell assessment of T-cell avidity (non-transduced: NT and B7-H3 CAR transduced: CAR) to SJ-DIPGX7c and U87-MG (N=3 T cell donors, mean and SEM are shown). (**H**) Area under the curve (AUC) of the percentage of bound cells over the increasing ramp of acoustic force (N=3 T cell donors, two-way ANOVA with Fisher least significant difference (LSD) test. *p<0.05. Mean and SEM are shown). ANOVA, analysis of variance; DMGs, diffuse midline gliomas; EPN, ependymoma; HGG, high-grade glioma; MB, medulloblastoma. Error bars represent mean and SEM.

To better understand why CAR T-cell activation is diminished against DMGs, we next examined the assembly of the IS, a highly organized structure that is initiated immediately on T-cell and tumor cell engagement. Effective IS formation is critical for efficient CAR-mediated signaling and subsequent anti-tumor response.^14 15^ We first characterized the quality of this interaction using live cell spinning disc confocal microscopy to quantify calcium (Ca^2+^) flux and dynamic changes in synapse area. Ca^2+^ flux and synaptic contact area are established indicators for T-cell activation^16^ and cell adhesion,^17^ respectively. CAR T-cells were labeled with CellTrace Violet and the calcium indicator Cal-520 AM, and tumor cells were labeled with CellTracker Red CMPTX prior to co-culture (figure 2A). To quantify the synaptic area, we measured the spatial intersection of T-cell and tumor-cell fluorescent masks.^18^ On DMG interaction, CAR T-cells exhibit a significantly reduced calcium response (figure 2A, B and D) and establish smaller synaptic contact areas compared with CAR T-cells interacting with U87-MG (figure 2A,C).

To elucidate if poor CAR T-cell interaction/activation is a distinct feature of DMGs, we performed the same live-cell imaging experiments as described above against high-grade glioma (HGG), ependymoma (EPN), medulloblastoma (MB) (online supplemental figure S4C and D). We normalized the calcium response to respective B7-H3 antigen density for each cell line (online supplemental figure S4A,B and E) to ensure that observed differences in calcium response between cell lines are not simply attributed to differences in antigen expression level. Overall, DMGs elicit a significantly lower calcium response compared with the broader cohort of non-DMG brain tumors (figure 2D and online supplemental figure S4E). We further demonstrate that the calcium response was significantly lower for CAR T cells against DMG compared with MB, GBM, and EPN, but was not significantly lower than that of a non-DMG pediatric HGG. These results suggest that poor CAR T-cell interaction/activation quality may be unique to DMGs and other HGGs.

Next, we hypothesized that small IS contact areas observed between CAR T cells and DMGs correspond with impaired IS formation at the molecular level. We performed confocal microscopy to quantify the accumulation of CAR, phosphorylated ZAP70 (pZAP70), lytic granules (LAMP1), and the F-actin cytoskeleton, as well as the recruitment of CD45 and the polarization of the centrosome (γ-Tubulin) at the synaptic interface after 30 min of interaction between CAR T cells and DMGs. Our results demonstrate that CAR T cells exhibit reduced accumulation of the CAR, CD45, pZAP70, and lytic granules at the interface with SJ-DIPGX7c compared with U87-MG, accompanied by impaired centrosome polarization (figure 2E,F, online supplemental figure 5). Notably, we observed that CAR T-cells exposed to SJ-DIPGX7c displayed an indistinct, unstructured distribution of F-actin at the IS (figure 2E). This observation further supports our finding that signaling and lytic machinery fail to properly accumulate during T-cell/DMG interaction.

To evaluate whether this dysfunctional IS formation also affected the physical interaction strength between CAR T-cells and tumor cells, we performed a z-MOVI single-cell avidity assay, which measures the precise binding force between CAR T-cells and tumor cells. Non-transduced (NT) T-cells were used as a control, because Delta-CAR T-cells, while not signaling, will interact with tumor cells in a similar manner as B7-H3 CAR T-cells and trigger a similar binding force. We show that CAR T-cell interaction with U87-MG is stronger compared with SJ-DIPGX7c (figure 2G,H). Collectively, these data establish that the CAR T-cell synapse is structurally weak and functionally deficient on engagement with DMG cells compared with other adult-type and pediatric-type brain tumors.

### Dysfunctional IS causes inert CAR T-cell transcriptional programs on interaction with DMGs

**Figure 3:**
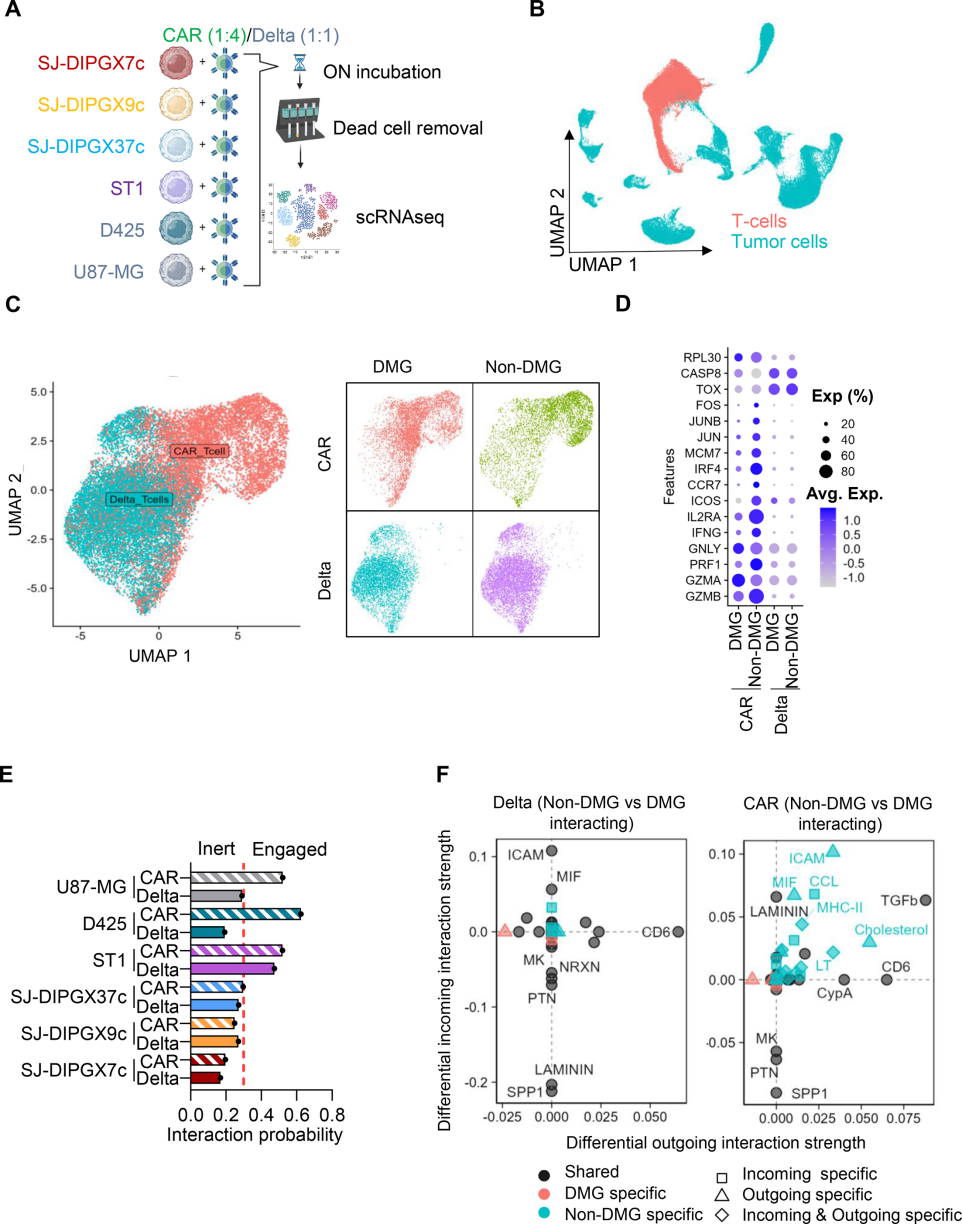
CAR T-cells have a low-activation and inert phenotype on DMG interaction. (**A**) Schematics of scRNA-seq assay in vitro co-culture of CAR T-cells against different brain tumor cell lines. CAR T-cell E:T ratio was 1:4 and Delta-CAR T-cell 1:1. Co-cultures were incubated overnight (ON), and live cells were magnetically selected prior to performing scRNA-seq. (**B**) UMAP representation of the co-culture scRNA-seq data, depicting T-cell and tumor cell clusters. (**C**) Uniform Manifold Aproximation and Projection (UMAP) representation of re-clustered T-cells depicting their functionality (Delta or CAR) and their interacting tumor (DMG (SJ-DIPGX7c, SJ-DIPGX9c, and SJ-DIPGX37c), non-DMG (ST1, D425, and U87-MG)). (**D**) Bubble plot of the differential gene expression between CAR and Delta-CAR T-cells interacting with DMG or non-DMG tumors. (**E**) CellChat weighted interaction probability between CAR and Delta-CAR T-cells against different tumor cells, defining inert and engaged T-cells regarding their relative interaction probability value (~0.3). (**F**) Interaction bubble plot, depicting the differences between non-DMG and DMG interacting T cells in terms of interaction pathway strength in incoming and outgoing signaling. DMG, diffuse midline glioma; E:T, effector to target; scRNA-seq, single-cell RNA sequencing.

To gain mechanistic insight into how DMGs are shaping the CAR T-cell transcriptional landscape on interaction, we performed scRNA-seq on T-cell and tumor cell co-cultures. B7-H3-CAR T-cells or Delta-CAR T cells were co-cultured with three DMG cell lines (SJ-DIPGX7c, SJ-DIPGX9c, and SJ-DIPGX37c), one EPN cell line (ST1), one MB cell line (D425), and one adult glioma cell line for comparison (U87-MG) (figure 3A). Tumor cells were co-cultured with B7-H3 CAR T-cells at a 1:4 E:T ratio and B7-H3 Delta-CAR T-cells at a 1:1 E:T ratio overnight. We chose a lower E:T ratio for the functional B7-H3 CAR compared with Delta-CAR to account for tumor cell loss due to the CAR T cell killing. In this way, sufficient live tumor and CAR T-cells remained in each condition for scRNA-seq. After overnight incubation, tumor cells and T cells were collected. Dead cells were removed using the Miltenyi Dead Cell Removal Kit, yielding highly viable samples for scRNAseq using the PARSE Bioscience single-cell whole transcriptome kit. Sequencing data was processed using the split pipeline (PARSE Bioscience) and analyzed in R studio^19^using Seurat^20^package. High-quality cells were filtered based on standard QC metrics. T-cell populations were identified from tumor cells by automatic cell annotation with SingleR^21^and CellDex^21^packages (figure 3B).

To explore broad transcriptomic differences in T-cells interacting with DMG versus non-DMG tumor cells (EPN, MB, and adult glioma), we clustered the T-cell population and evaluated the expression of effector-associated genes. Our analysis revealed that transcriptional differences between DMG and non-DMG conditions were unique to signaling CAR T-cells (figure 3C,D). Specifically, CAR T-cells interacting with non-DMG tumors showed significantly higher expression of genes associated with cytotoxicity (PRF1 and GMZB), activation (ICOS, IL2RA, and IFNG), and proliferation (JUN, JUNB, and MCM7) compared with DMG-interacting CAR T-cells. Next, we performed Gene Set Enrichment Analysis (GSEA) utilizing Hallmark, KEGG, and GO databases. We found that pathways associated with active T cell function, such as cytokine signaling (examples: HALLMARK IL2/STAT5, IL6/JAK STAT3 and KEGG STAT signaling), cell adhesion and cytoskeleton remodeling (examples: KEGG regulation of actin cytoskeleton and GO regulation of cell adhesion) and T cell activation (examples: HALLMARK KRAS signaling, KEGG MAPK pathway, GO MAPK cascade) are suppressed in DMG-interacting CAR T-cells (online supplemental figure S6A-C). These transcriptomic differences support our functional findings, demonstrating that the dysfunctional IS formation between CAR T-cells and DMGs results in a transcriptionally attenuated response in CAR T-cells.

To explore whether this difference in CAR T-cell phenotype correlates with specific tumor and CAR T-cell interaction pathways, we performed an unbiased assessment of cell-to-cell communication using CellChat.^22^ We analyzed twelve distinct interaction pairs, consisting of either CAR or Delta-CAR T-cells co-cultured with six different tumor models: three DMGs (SJ-DIPGX7c, SJ-DIPGX9c, SJ-DIPGX37c), one ependymoma (ST1), one MB (D425), and one adult glioma (U87-MG). As expected, cell lines interacting with Delta-CAR T-cells exhibited a low total communication probability (figure 3E) with the exception of ST1. This was not surprising, as ST1 cells are prone to inducing unspecific Delta-CAR T-cell stimulation/expansion.^23^ Thus, data from ST1 cells serve as a quality control and support the validity of our approach. When tumor cells were co-cultured with functional B7-H3-specific CAR T cells, the overall interaction probability was increased against U87-MG, D425, and mildly increased against ST1 (figure 3E). However, the communication probability between CAR T-cells and DMG cells failed to increase beyond Delta-CAR T-cell levels across all the three DMG lines evaluated (figure 3E). Further pathway analysis revealed that DMG cells consistently suppressed multiple pathway modules, such as co-stimulatory and cell adhesion interaction pathways (online supplemental figure S6D). Our results indicate that non-DMG-interacting CAR T cells elicit a robustly engaged transcriptional phenotype, but DMG-interacting CAR T cells remain transcriptionally inert.

We next evaluated the differences in the relative contributions of incoming and outgoing interactions between non-DMG and DMG tumor cells and engineered T-cells. As expected, interaction pathway profiles were not significantly altered among Delta-CAR T-cells across all interactions. In contrast, functional CAR T-cells showed significant differential expression of several key pathways (figure 3F). Notably, the intercellular adhesion molecule (ICAM) signaling pathway was the most significantly upregulated incoming signal in the non-DMG and CAR T cells interaction (figure 3F).

To further explore the ICAM signaling axis, we interrogated the tumor cell transcriptome. The ST1 ependymoma data were excluded from this analysis because they had a high basal interaction probability, which may mask key interactions or lead to artifactual findings. First, we show that tumor cells indeed undergo shifts in transcriptional profiles on functional CAR T-cell interaction compared with Delta-CAR T-cell interaction (online supplemental figure S7A). Additionally, GSEA revealed that non-DMG tumors enrich for adhesion and inflammatory responses compared with DMGs (online supplemental figure S7B). As expected, CellChat analysis of tumor cells revealed that ICAM signaling was significantly downregulated as an outgoing signal in DMGs compared with non-DMGs (online supplemental figure S7C), and this was associated with downregulation of *ICAM-1* expression at mRNA level (online supplemental figure S7D).

To validate this finding at the protein level, we performed flow cytometric analysis of ICAM-1 and other immunomodulatory ligands, such as PD-L1 and CD58, first on tumor cells and then, after overnight culture with CAR T cells at a 1:4 E:T ratio. For this, we chose SJ-DIPGX7c, U87-MG, and D425 as they have similar B7-H3 antigen density. We found that U87-MG uniquely had significantly higher baseline expression of ICAM-1 and PD-L1 compared with SJ-DIPGX7c and D425, but all cell lines had equivalent expression of CD58 (online supplemental figure S8). on overnight co-culture with B7-H3 CAR T-cells, all cell lines increased expression of ICAM-1 and PD-L1 compared with Delta-CAR T-cells, and CD58 expression was unchanged (online supplemental figure S9). However, SJ-DIPGX7c showed significantly less upregulation of ICAM-1 than U87-MG and D425, and no trends were observed for PD-L1 (online supplemental figure S9A and B). Altogether, our data supports that DMGs have a deficient interaction with CAR T-cells, possibly driven by low ICAM-1 expression and regulation, which results in a transcriptionally inert CAR T-cell phenotype.

### RASA2 KO improves IS quality

We next sought to determine if the CAR T-cell dysfunctional phenotype on DMG interaction could be overcome by targeting IS formation pathways. As proof of concept, we deleted RASA2 via CRISPR/Cas9 technology in CAR T-cells. The Ras signaling pathway is essential in IS assembly, orchestrating both cytokine signaling^24^ and cell adhesion.^25^ Consequently, the RAS pathway is considered one of the master regulators of T-cell activation,^26^ a role that is consistent with our scRNA-seq findings showing downregulation of the KRAS pathway in CAR T-cells targeting DMGs. We have previously demonstrated that RASA2 deletion in CAR T-cells enhances Ras/MAPK signaling and improves overall CAR T-cell potency.^12^ However, the mechanism by which RASA2 deletion improves IS quality in CAR T cells against DMG remains unclear.

To evaluate this, we deleted RASA2 in CAR T-cells using CRISPR-Cas9 gene-editing, using AAVS1 locus targeting guide-RNA (gRNA) as a control (Ctrl-KO).^12^ Successful RASA2 deletion in CAR T-cells was confirmed by Western Blot (online supplemental figure S10A). RASA2 deletion did not alter CAR transduction efficiency (onlinesupplemental figures 10B,C  S12A,B), nor did it impact CD4/CD8 ratio or effector/memory phenotype (online supplemental figure 10D-G). Consistent with previously published work,^12^ RASA2 deletion increased CD69 surface expression (onlinesupplemental figures 10H,I  S12C,D), indicating that RASA2-KO CAR T-cells are more activated at basal levels compared with Ctrl KO CAR T-cells.

**Figure 4:**
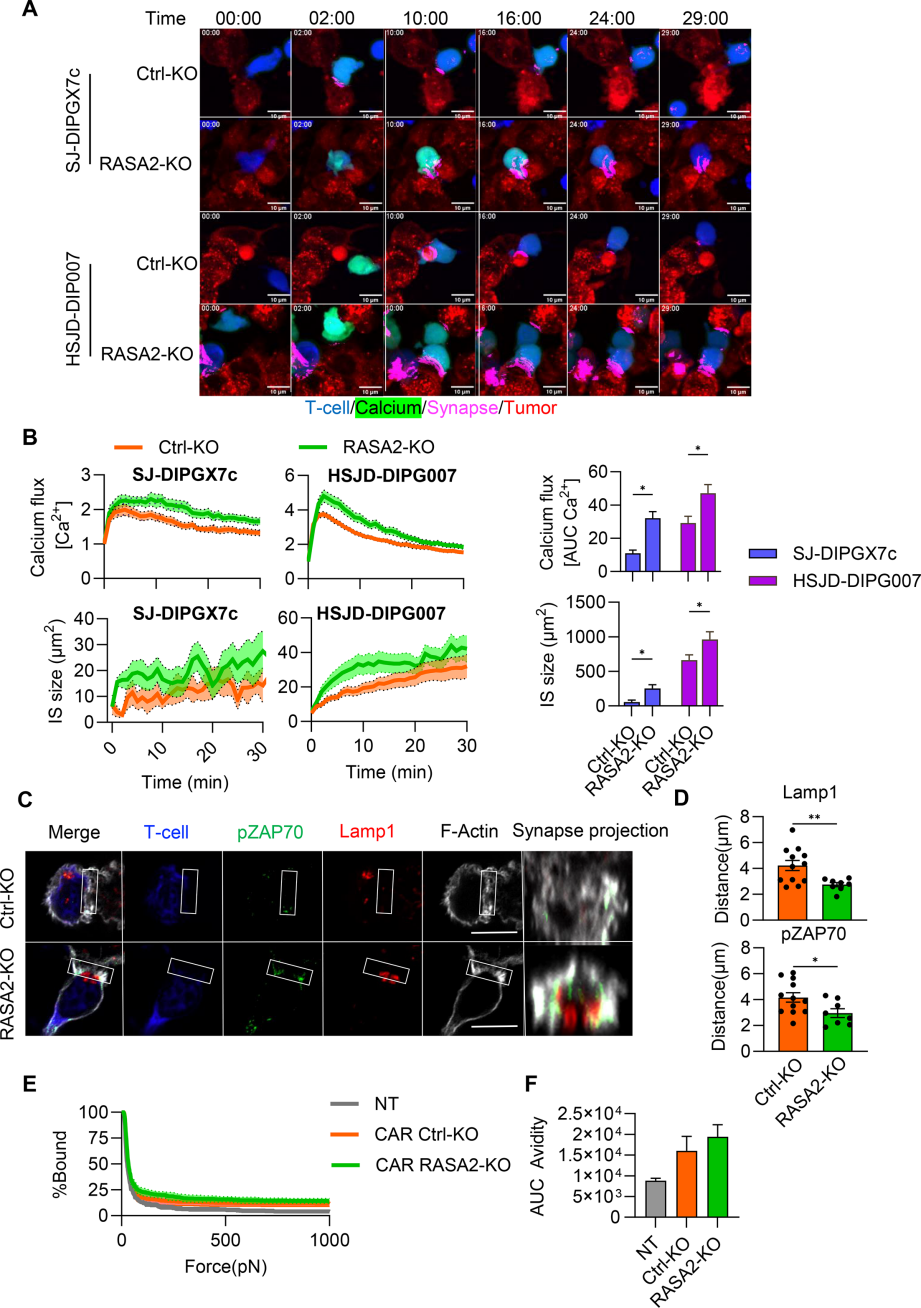
RASA2-KO improves the immune synapse formation on tumor cell interaction. (**A**) Representative time-lapse images of CAR (Ctrl-KO and RASA2-KO) T-cells interacting with DMGs (SJ-DIPGX7c and HSJD-DIPG007) acquired by confocal live cell imaging. CAR T-cells were labeled with Cell Trace violet (Blue) and CAL520 (Green), tumor cells were labeled with CellTracker Red-CMTPX (Red), Synapse was automatically generated as the intersection between T-cell and tumor cell labeling (Magenta) (mean Z-projection is shown scale bar=10 µm). (**B**) Quantification of calcium flux and synaptic size in CAR T-cells on tumor cell interaction, against SJ-DIPGX7c and HSJD-DIPG007, and their respective area under the curve (AUC) analysis (N=2 T cell donors, cells=27–68 total, Two-way ANOVA, Two-stage linear step-up procedure of Benjamini, Krieger, and Yekutieli test, *p<0.0001). Error bars represent mean and SEM. (**C**) Representative confocal images of CAR (Ctrl-KO and RASA2-KO) T-cells (blue) and SJ-DIPGX7c tumor cells interacting after 30 min of co-culture. pZAP70 (green), Lytic granules (Lamp1) (Red), and F-actin (gray), merge, and synapse projection are shown (single Z-plane is shown scale bar=10 µm). (**D**) Quantification of the accumulation of lytic granules (Lamp1) and pZAP70 at the immune synapse, measured as the distance of the center of mass of the labeling towards the synapse (smaller values indicate increased accumulation). (N=2 T cell donors, cells=6–7 per donor, Unpaired t-test. **p=0.0094, *p=0.034). Error bars represent mean and SEM. (**E**) Single cell assessment of T-cell avidity (non-transduced (NT), CAR Ctrl-KO, and CAR RASA2-KO) to SJ-DIPGX7c (N=3 T cell donors, mean and SEM are shown). (**F**) Area under the curve (AUC) of the percentage of bound cells over the increasing ramp of acoustic force (N=3 T cell donors). Error bars represent mean and SEM. ANOVA, analysis of variance; IS, immune synapse.

We next investigated whether RASA2-KO improves CAR T-cell activation on DMG interaction by quantifying calcium flux and synaptic contact area via live cell imaging. Our results show that RASA2-KO increases both calcium flux and the synaptic area in CAR T-cells across multiple DMG cell lines (SJ-DIPGX7c, SJ-DIPGX9c, SJ-DIPGX29c, SJ-DIPGX37c, and HSJD-DIPG007) (figure 4A,B and online supplemental figure S11A-C). This improvement was not DMG-specific as RASA2-KO in CAR T-cells improved IS formation when cultured with GBM (U87-MG), HGG (SJ-HGGX42c), and MB (DAOY) cells, as evidenced by significantly higher Ca^2+^ flux and IS size compared with Ctrl-KO CAR T-cells (online supplemental figure S11A-C). We further determined that the improvement in CAR T-cell activation conferred by RASA2-KO was not constricted to CAR specificity, as IL13Rα2^27^-targeting RASA2-KO CAR T-cells also showed improved Ca^2+^ flux on interaction with DMG (SJ-DIPGX7c and HSJD-DIP007) compared with Ctrl-KO CAR T-cells (online supplemental figure S12E-H).

To determine if RASA2-KO also improved the molecular organization of the IS, we quantified the accumulation of signaling molecules (pZAP70) and lytic granules (Lamp1) at the synaptic interface between B7H3-CAR T-cells and SJ-DIPGX7c cells (figure 4C). Our results show that RASA2-KO CAR T-cells exhibited higher accumulation of pZAP70 and lytic granules at the synaptic interface (figure 4D), suggesting a restoration of IS molecular organization. Finally, we performed a single-cell avidity assay (as described in figure 2G) to determine if RASA2-KO increases binding affinity. RASA2 ablation in CAR T-cells resulted in a slight upward trend in binding strength against SJ-DIPGX7c but did not reach statistical significance (figure 4E,F). Collectively, these findings show that RASA2-KO improves Ca^2+^ flux, synapse size, and overall IS quality, regardless of the interacting tumor or the CAR target, without fundamentally altering the relative physical interaction strength.

### RASA2 deletion enhances actin cytoskeleton dynamics and CAR accumulation at the IS in T-cells

Thus far, our results show that RASA2-KO improves the IS of B7-H3 CAR T-cells interacting with DMGs. We next sought to determine if stimulation of the CAR alone is sufficient for IS improvement. To test this, we activated B7-H3 CAR T-cells on coverslips coated with only recombinant human B7-H3 for 30 min. We then fixed the samples and analyzed the IS structure by confocal microscopy. Consistent with our previous results, we observed significantly higher accumulation of pZAP70 and lytic granules at the synaptic interface of RASA2-KO CAR T cells, significantly increased synaptic area and F-actin accumulation, and formation of prominent lamellipodia- and filopodia-like structures (online supplemental figure S13A-E). This suggests that CAR triggering alone in the absence of other co-receptors present on tumor cells is sufficient for RASA2-KO to improve the IS quality.

**Figure 5:**
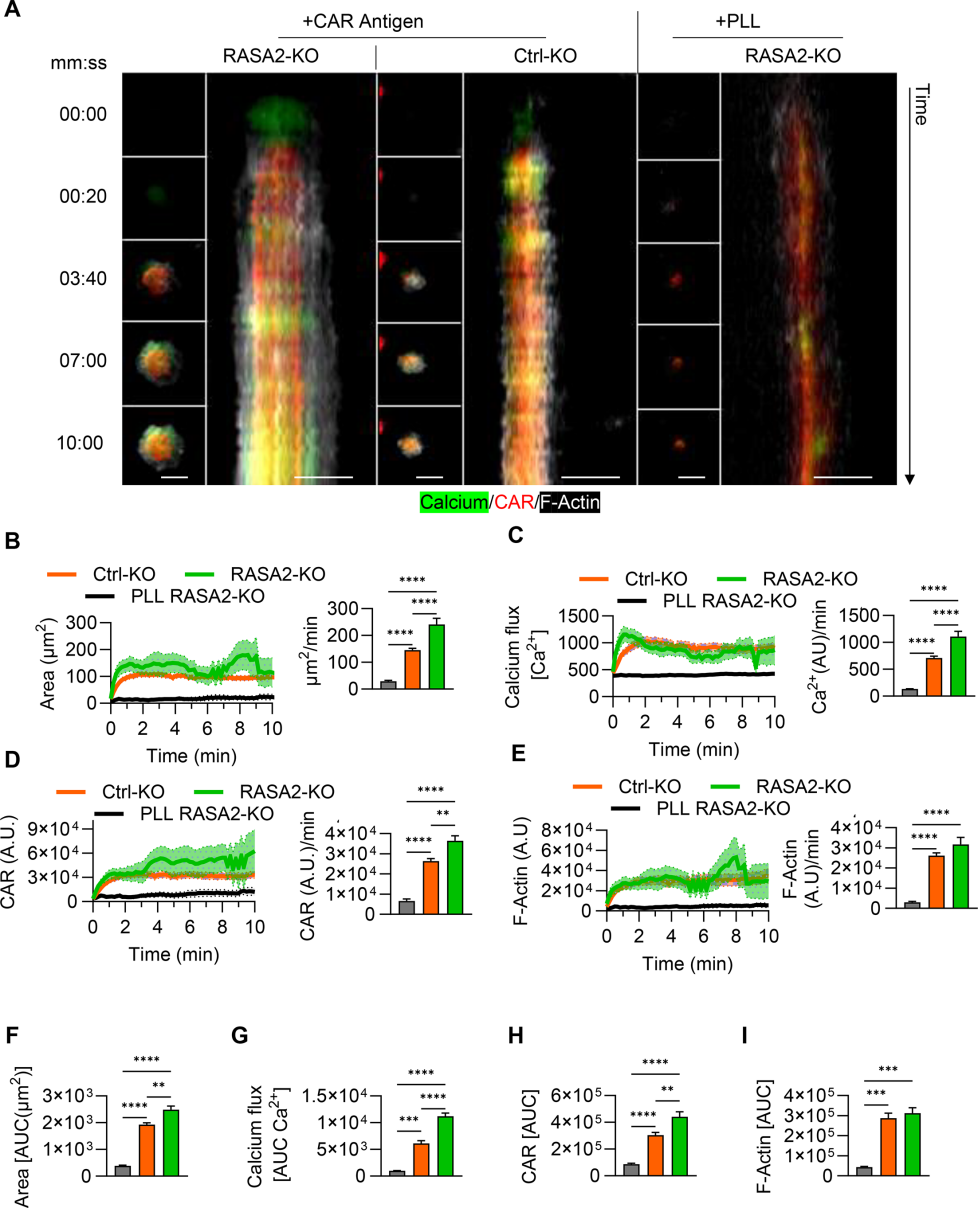
RASA2-KO increases the spreading speed, calcium flux response, CAR molecule accumulation, and actin polymerization at the IS on antigen engagement. (**A**) Representative time-lapse images and re-slices (x-axis represents synapse size and y-axis represents time) of IL13Rα2-CAR T-cells activated onto IL13Rα2-coated surfaces, imaged at the synaptic interface by TIRFM (scale bar=10 µm). (**B**) Quantification of the synaptic size over time, and the spreading speed during the first minute of activation. (**C**) Quantification of calcium flux over time, and the calcium response speed during the first minute of activation. (**D**) Quantification of CAR molecule recruitment over time, and the speed of CAR recruitment during the first minute of activation. (**E**) Quantification of F-actin recruitment over time, and the F-actin accumulation speed at the first minute of activation. (**F–I**) Quantification of the overall synaptic size, calcium flux, CAR recruitment, and F-actin accumulation at the IS, respectively (N=2 T cell donors, total cells=6–31, unpaired t-test. **p<0.01, ***p<0.001, ****p<0.0001). Error bars represent mean and SEM. AUC, area under the curve; IS, immune synapse; PLL, Poly-L-Lysine; TIRFM, total internal reflection microscopy.

We next sought to observe the dynamics of IS formation in RASA2-KO vs Ctrl-KO CAR T-cells. Effective IS formation in CAR T-cells requires the coordinated recruitment and clustering of CAR molecules^28^and rapid actin cytoskeleton remodeling.^29^ To test whether RASA2-KO enhances these processes, we performed total internal reflection microscopy (TIRFM) to visualize the synapse structure in real-time with high axial resolution (~100 nm).^30^ To track CAR molecule recruitment and clustering, we used an IL13Rα2-CAR,^27^ which was directly fused to the mClover fluorescent protein. This CAR was selected because it was the only readily available construct in our hands with a direct mClover fusion, enabling real-time tracking of CAR molecules. F-actin dynamics and Ca^2+^ flux were visualized using SPY650-FastAct and CAL-590 AM, respectively. CAR T-cells were activated on 30 mm glass-bottom dishes coated with 10 µg/mL IL13Rα2 recombinant protein, while 0.01% Poly-L-Lysine (PLL) coated dishes served as an unstimulated control. on antigen engagement, CAR T-cells exhibited significant spreading, CAR clustering, and Ca^2+^ flux, which were absent in PLL controls (figure 5A). The magnitude and kinetics of these responses were significantly enhanced in RASA2-KO CAR T-cells compared with Ctrl-KO CAR T-cells (figure 5A). RASA2-KO accelerated the initial response, as evidenced by increased speeds in spreading (figure 5A,B), Ca^2+^ flux (figure 5A,C), CAR recruitment (figure 5A,D), and F-Actin polymerization (figure 5A,E) within the first minute of activation. RASA2-KO CAR T cells exhibited overall larger synaptic areas (figure 5F), increased cumulative Ca^2+^ response (figure 5G), and increased CAR recruitment (figure 5H) over a 10 min period. However, there were no significant changes in the overall accumulation of F-Actin at the synaptic interface (figure 5I), suggesting that RASA2-KO primarily modulates the speed of F-Actin remodeling rather than its total density.

To test whether this effect of RASA2 deletion is unique to CAR biology, we activated T cells through the endogenous TCR using glass-bottom dishes coated with 10 µg/mL CD3/CD28 antibodies and performed TIRFM. Both Ctrl-KO and RASA2-KO T-cells showed characteristic cell spreading, Ca^2+^ flux, and polarization (evidenced by centrosome docking at the synaptic interface) on stimulation (online supplemental figure S13F). Consistent with our CAR-based findings, RASA2-KO T-cells established larger synapses accompanied by a slight increase in Ca^2+^ flux and the formation of actin-foci structures at the IS (online supplemental figure S13F,G). Additionally, RASA2-KO T-cells exhibited accelerated centrosome docking to the synaptic interface compared with Ctrl-KO T-cells (online supplemental figure S13F,G). Overall, these results demonstrate that RASA2-KO is a universal tool for improving IS formation and dynamics in T-cells, whether they are engaged via the endogenous TCR or synthetic CAR molecule.

Together, our results indicate that stimulation of the CAR or TCR alone is sufficient for RASA2 deletion to enhance IS synapse formation and dynamics. Additionally, we show that RASA2-KO consistently enhances IS size and speed of formation.

### RASA2 deletion in CAR T-cells improves cytotoxicity but not persistence

To interrogate whether enhanced IS formation in RASA2-KO CAR T-cells rescues core effector functions in vitro (cytotoxicity, expansion, and cytokine secretion) against DMGs, we first evaluated killing kinetics by live-cell imaging (as described in online supplemental figure S2). Our results demonstrate that the deletion of RASA2 in B7-H3 CAR T-cells increased the killing speed against both SJ-DIPGX7c (low B7-H3 expression; online supplemental figure S14A and B) and HSJD-DIPG007 (high B7-H3 expression; online supplemental figure S14A,C), indicating that RASA2-KO improves cytotoxic efficiency. We next evaluated the proliferation capacity using the previously described repeat stimulation assay. RASA2-KO CAR T-cells exhibited a moderate increase in cumulative expansion against all DMG cell lines and persisted an average of 2 additional stimulations against SJ-DIPGX7c only (online supplemental figure S15A).

We next characterized CAR T-cell cytokine secretion using Milliplex human cytokine multiplex assay. Supernatants collected 24 hours post-first tumor cell exposure revealed that GM-CSF, TNF-α, and IL-2 were significantly increased in RASA2-KO CAR T-cells across different DMGs (SJ-DIPGX7c, SJ-DIPGX37c, and HSJD-DIPG007) compared with Ctrl-KO cells (online supplemental figure S15B). This contributed to an overall improvement in the Th1 response (online supplemental figure S15C). Similar trends were observed with IL13Rα2 CAR T cells (online supplemental figure S16).

Altogether, these data establish that RASA2 deletion improves CAR T-cell effector functions against DMGs in vitro. Specifically, we evidenced improved CAR T-cell cytotoxicity and Th1-type cytokine secretion but no significant differences in T-cell expansion or persistence.

### RASA2-KO CAR T-cells have a rapid initial anti-tumor response in vivo

**Figure 6:**
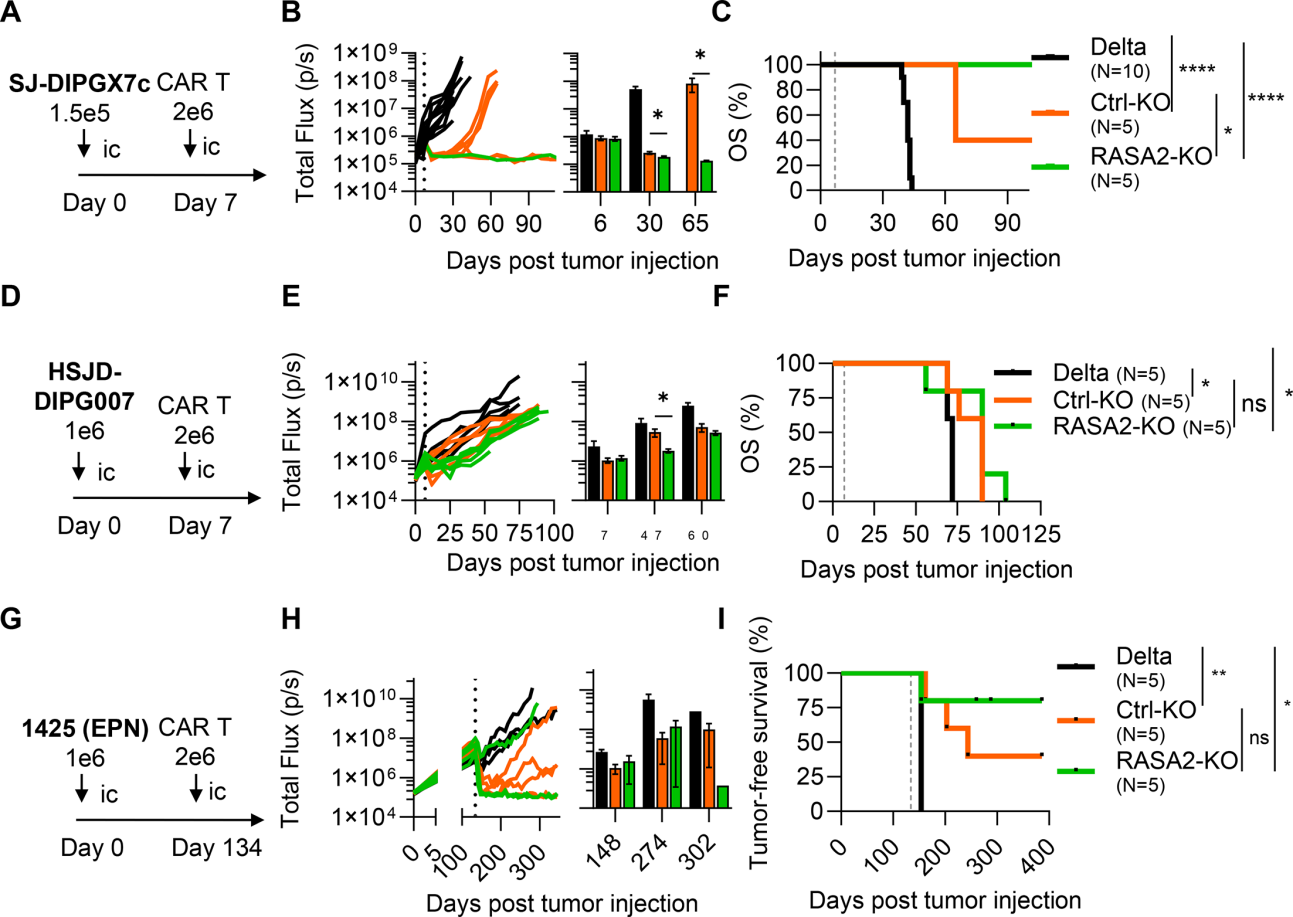
RASA2-KO improves early in vivo anti-tumor response against DMG and ependymomas. (**A, D**, **G**) Schematic of the in vivo experimental design for tumor intracranial implantation model. Tumor cells were implanted intracranially (ic) into the brain cortex, followed by a single ic dose of 2×10^6^ B7-H3-(Ctrl- and RASA2-KO) or Delta-CAR T-cells after confirmation of tumor engraftment. (**B, E, H**) Total flux from tumor cells in all mice treated with CAR T-cells. The tumors were measured weekly using bioluminescence imaging. Dashed lines represent the time of CAR T-cell treatment (multiple Mann-Whitney U test, *p<0.05). Error bars represent mean and SEM. (**C, F, I**) Kaplan-Meier survival analysis of mice treated with CAR T-cells (dashed line indicates the time of CAR T-cell treatment) log rank (Mantel-Cox test, N=5–10, *p<0.05, **p<0.001, ****p<0.0001). DMG, diffuse midline glioma; EPN, ependymoma.

To evaluate whether RASA2 KO in CAR T cells renders a therapeutic benefit against DMGs, we tested the in vivo efficacy of RASA2-KO B7-H3-CAR T-cells against two patient-derived orthotopic xenograft models: SJ-DIPGX7c and HSJD-DIPG007. Our results demonstrate that RASA2-KO improved tumor burden control in both models (figure 6A, B, D and E). However, overall survival was extended only against SJ-DIPGX7c (figure 6C) but not HSJD-DIPG007 (figure 6F).

We next tested if RASA2-KO could improve CAR T-cell efficacy against non-DMG brain tumors. For this, we implanted the 1425 EPN cell line^31^ into the cortex of NSG mice (figure 6G). Treatment with RASA2-KO or Ctrl-KO CAR T cells was initiated on MRI confirmation of established tumors. Although RASA2-KO CAR T-cells achieved complete tumor eradication in 80% of mice (4/5), compared with 40% (2/5) in control-KO CAR T-cells, this improvement did not translate into a significant survival benefit (figure 6H,I).

Finally, we tested the in vivo efficacy of RASA2-KO IL13Rα2-specific CAR T-cells against SJ-DIPGX7c (online supplemental figure S17A). RASA2-KO IL13Ra2-specific CAR T-cells elicited a rapid initial anti-tumor response and maintained tumor control for 30–40 days postinfusion (online supplemental figure 17), a pattern consistent with our observations in the HSJD-DIPG007 model (figure 6D–F). However, RASA2-KO did not confer a survival advantage (online supplemental figure 17).

### Enhanced adhesion and migration contribute to early in vivo responses but lack of persistence remains a challenge

Our in vivo data demonstrate that RASA2-KO CAR T-cells exhibit a potent initial anti-tumor response but fail to prevent disease progression. Thus, we sought to determine mechanisms that govern these responses. We first treated HSJD-DIPG007-bearing mice with Ctrl-KO or RASA2-KO CAR T-cells, harvested CAR T-cells from the brain after 3 days, and performed whole-transcriptome analysis (online supplemental figure S18A). Consistent with our in vitro findings, GSEA revealed that RASA2-KO CAR T-cells in vivo have significant enrichment in cell adhesion pathways (online supplemental figure S18B). Additionally, there were no significant differences in cytokine and chemokine expression and exhaustion phenotype except for T-cell immunocreceptor with Ig and ITIM domains (TIGIT), which was significantly downregulated in the RASA2-KO condition (online supplemental figure 18C,D). We further observed significant upregulation of cytoskeleton-related genes *MYO10, MYH9,* and *CDC42BPB* (online supplemental figure S18D) which are involved in T cell motility and migration.^32^,^34^

**Figure 7:**
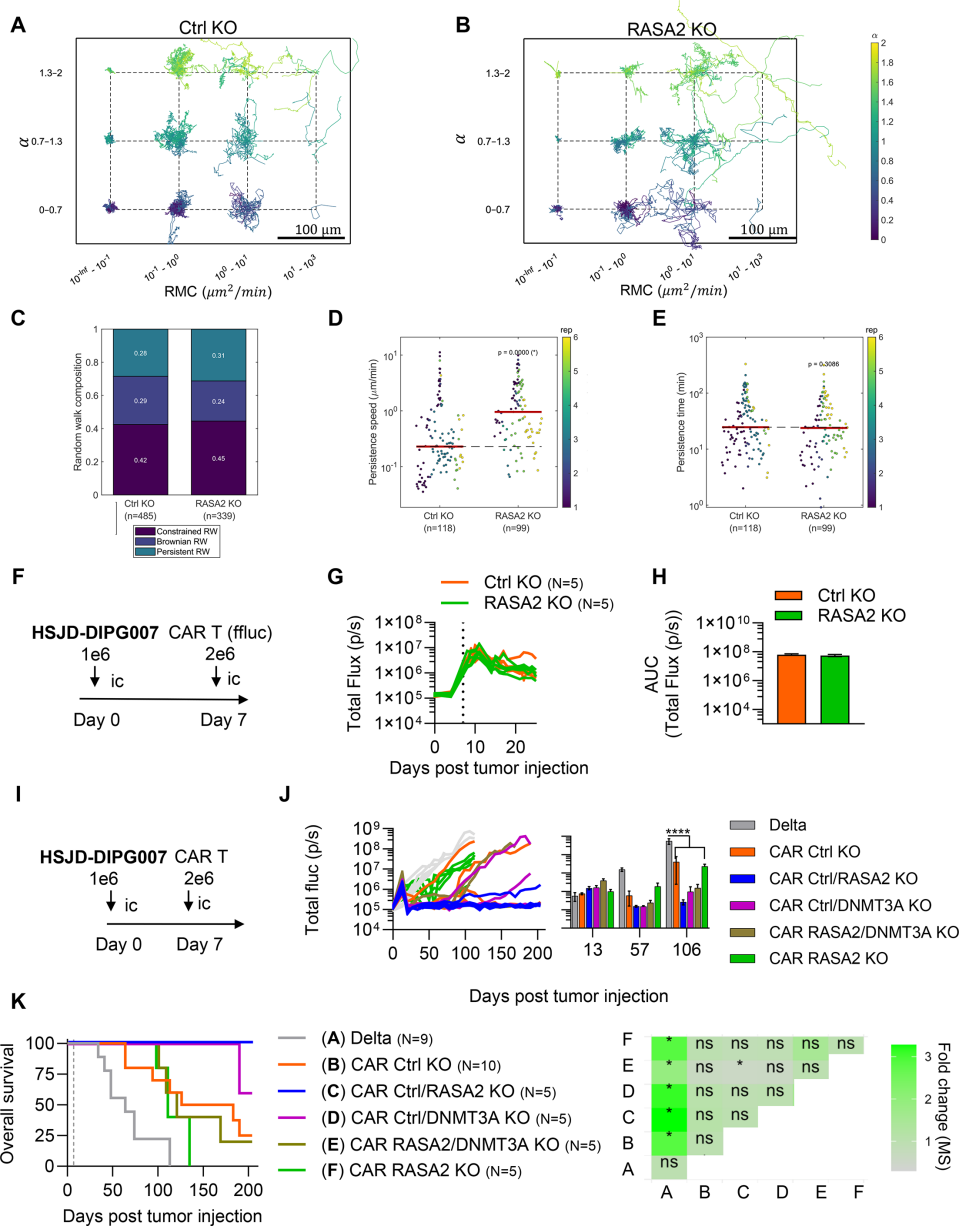
RASA2-KO improves migration, but persistence is still a limitation for long-lasting CAR T-cell antitumor response. (**A, B**) Windrose plots of T-cell trajectories for Ctrl-KO and RASA2-KO. (**C**) The stacked bars report the fraction of tracks assigned to Random Walk (RW) models (Persistent, Brownian, and constrained). Individual tracks fitted to the persistent random walk (PRW) model, showing persistence speed (**D**) and time (**E**). (N=3, cells=67–238, Kruskal-Wallis tests, *p<0.05). (**F**) Schematic of the in vivo experimental design for tumor intracranial implantation model. Tumor cells were implanted intracranially (ic) into the brain cortex, followed by a single ic dose of 2×10^6^ B7-H3-(Ctrl- and RASA2-KO) expressing GFP-firefly luciferase 7 days post-tumor injection (N=5 mice per group). (**G**) Total flux from T-cells in all mice treated with CAR T-cells. (**H**) Bar graph showing the total T-cell bioluminescence, calculated as the area under the curve (AUC) from (**G**). Error bars represent mean and SEM. (**I**) Schematic of the in vivo experimental design for DMG intracranial implant model. HSJD-DIPG007 cells (1×10^6^) were implanted intracranially (ic) into the brain cortex, followed by a single ic dose of 2×10^6^ B7-H3- (KOs mix) or Delta-CAR T-cells 7 days post-tumor injection. (**J**) Total flux from tumor cells in all mice treated with CAR T-cells. The tumors were measured weekly using bioluminescence imaging (Uncorrected Fisher's LSD test,****p<0,0001). Error bars represent mean and SEM. (**K**) Kaplan-Meier survival analysis of mice treated with CAR T-cells, represented by the gray dashed line (log rank (Mantel-Cox) test, N=5–10). The heatmap shows the fold change in the median overall survival between all conditions against Delta-treated mice, and their respective p values (*p<0.05). DMG, diffuse midline glioma; RMC, random motility coefficient.

To validate RASA2-KO enhancement of CAR T cell motility, we evaluated the migration of RASA2-KO CAR T cells using an established ex vivo brain slice platform.^35^ Fluorescently labeled (CellTracker Green CMFDA) CAR T-cells were applied to freshly prepared 300 µm thick coronal brain slices stained with isolectin GS-IB4 for vasculature reference. Brain slices were imaged in an inverted Zeiss LSM 7 line-scanning confocal microscope at 37°C in 5% CO_2_ with a frame interval of 2 min for 3–9 hours. Images were processed and analyzed using TrackMate^36^ and MATLAB.^37^ To describe the migratory properties of CAR T-cells, we computed the mean squared displacement (MSD) for each track and fitted well-established diffusion models^38 39^ (Methods). Our results demonstrate that RASA2 deletion significantly enhanced multiple parameters of CAR T-cell motility, including median track speed (online supplemental figure S19A) and random motility coefficient (online supplemental figure 19). RASA2-KO CAR T-cells also showed longer trajectories compared with Ctrl-KO T-cells as evidenced by the Windrose plot (figure 7A,B). Next, using corrected Akaike information criterion (AICc), we determined which diffusion model best described each T-cell track, and we found that RASA2 deletion increased the number of tracks following constrained-like and persistent-like diffusion models, with the concomitant reduction of the Brownian-like diffusion (figure 7C). For the T-cell tracks that were best described by the Persistent random walk model, a typical diffusion model for describing T-cell migration, RASA2-KO increased the persistence speed (figure 7D) without affecting the persistence time (figure 7E). These data suggest that RASA2-KO indeed enhances CAR T-cell migration, which together with adhesion, may contribute to improved early in vivo responses.

We next sought to determine if the lack of RASA2-KO CAR T-cell long-term anti-tumor control was associated with poor persistence. We treated mice bearing wild-type HSJD-DIPG007 (no ffluc) with ffluc-expressing Ctrl-KO and RASA2-KO CAR T-cells and tracked bioluminescence over time to evaluate the persistence. Both Ctrl-KO and RASA2-KO CAR T-cells had peak bioluminescence signal at 3 days post CAR T-cell injection that slowly dissipated over time (figure 7F,G). Area under the curve analysis revealed that CAR T cell persistence is not improved by RASA2-KO (figure 7H), suggesting that lack of persistence is a challenge that RASA2-KO alone does not overcome.

We previously demonstrated that the deletion of DNA (cytosine-5)-methyltransferase 3A (DNMT3A) effectively sustains long-term T-cell persistence.^40^ We hypothesized that incorporating a persistence-enhanced population (DNMT3A KO) would facilitate the eradication of recurrent tumor cells spared by RASA2-KO CAR T-cells. To test this, we performed an in vivo experiment using mixed B7-H3-CAR T-cell products containing RASA2 KO (for rapid tumor debulking) or DNMT3A KO (for long-term surveillance) at a 1:1 ratio (figure 7I, online supplemental figure 20).

Contrary to our expectations, the addition of DNMT3A-KO CAR T-cells did not enhance long-term anti-tumor control (figure 7J). Surprisingly, co-infusing RASA2-KO B7-H3 CAR T-cells mixed with Ctrl-KO B7-H3 CAR T-cells (1:1 ratio) resulted in a durable anti-tumor response (figure 7J), leading to a significant improvement in overall survival compared with control mice (figure 7K). These unexpected findings may be partially explained by the requirement for a heterogeneous T cell population and diverse phenotypes in an effective CAR T-cell response.^41 42^

## Discussion

Here, we demonstrate for the first time that the limited anti-tumor response of CAR T-cells against DMGs is, among other causes, due to the formation of a low-quality, dysfunctional IS. This synaptic deficiency is characterized by attenuated calcium flux and impaired polarization to the synaptic interface between CAR T-cells and DMGs. Consequently, these defects lead to reduced CAR T-cell activation (evidenced by diminished cytokine secretion and reduced expansion), cytotoxicity, and overall anti-tumor activity against DMGs. Finally, we established that this IS formation deficiency can be circumvented through the deletion of RASA2 in CAR T-cells. By optimizing the IS architecture and dynamics, RASA2-deleted CAR T-cell showed superior effector function and enhanced therapeutic potency against DMGs and other pediatric brain tumors.

Previous studies have established that antigen density on target cells, together with CAR biophysical properties (affinity, avidity, and density), is the primary determinant of CAR T-cell response.^43 44^ High antigen density or high CAR affinity can lead to rapid exhaustion, whereas low antigen density or affinity renders CAR T-cells ineffective.^45^ However, our findings demonstrate that DMG-specific factors can override this antigen-density dependency, impairing CAR T-cell function even at high antigen levels.

Furthermore, our comparative analysis across pediatric brain tumors (EPN, MBs, DMGs, and HGGs) identifies DMGs as the most profoundly suppressive tumor type for CAR T-cell activation and function. Of interest, DMGs uniquely suppressed GZMB/PRF1 cytotoxic signatures but resulted in upregulation of GZMA and GNLY cytotoxic signatures in CAR T-cells. Previous reports have shown that GZMB-mediated cytotoxicity is more efficient than GZMA-mediated cytotoxicity.^46^ This suggests that DMGs may uniquely suppress more efficient killing mechanisms, forcing CAR T-cells to compensate by using less efficient strategies. While the specific mechanisms of this suppression remain to be fully elucidated, our data identified the IS formation as the keystone of this inhibitory mechanism. This is evidenced by a dysfunctional IS that fails to trigger optimal signaling and structural remodeling necessary for effective anti-tumor response.

Our scRNA-seq data showed that the Ras signaling pathway is significantly suppressed in CAR T-cells interacting with DMGs. As a proof of concept, we targeted this pathway by deleting RASA2, building on our previous experience.^12^ While this strategy resulted in improved IS formation, it is possible that targeting other impaired pathways, such as JAK/STAT signaling, could yield comparable or superior therapeutic outcomes.

We previously demonstrated that optimizing the IS should lead to higher avidity between CAR T-cells and tumor cells.^11^ Given the robust enhancement in IS structure and dynamics, it was unexpected to find that deleting RASA2 in CAR T-cells marginally strengthened the interaction between RASA2-KO CAR T-cells and DMG cells, without reaching statistical significance. The absence of stronger interactions despite optimized IS formation suggests that other factors may be influencing the interaction between CAR T-cells and DMG cells. This observation further supports our hypothesis that dysfunctional IS formation is driven by DMG-specific factors. Further analyses suggested that a likely candidate for this impaired T-cell and DMG interaction might be the low expression of adhesion molecules, such as ICAM-1.^47^,^49^ ICAM-1 is a critical ligand for LFA-1-mediated T-cell adhesion,^50 51^ and its low expression in DMGs, which are characterized by their non-inflammatory nature,^52^ likely contributes to their low-avidity phenotype. Furthermore, since ICAM-1 expression is typically induced by inflammatory cytokines, such as IFN-γ and TNF-α,^50 53^ the diminished cytokine production by CAR T-cells on DMG interaction might drive an insufficient signal to upregulate adhesion molecules. Therefore, while we have successfully improved IS formation from the CAR T-cell perspective, the tumor-intrinsic factors that maintain a dysfunctional IS remain a critical area for future investigation.

We identified that RASA2-KO CAR T-cells have potent initial anti-tumor responses in vivo, but persistence limits durable responses. This is consistent with previous findings from large-scale in vivo CRISPR screens, which showed that RASA2-KO CAR T cells were enriched at early time points but did not persist long-term against hematological malignancies.^54^ Ongoing clinical trials for DMGs and other brain tumors incorporate multiple CAR T-cell infusions (NCT04196413, NCT05768880, NCT05835687), which may overcome the need for CAR T-cell persistence. In this context, the enhanced initial potency of RASA2-KO CAR T-cells may be particularly beneficial at each infusion. Therefore, preclinical modeling using multiple CAR T-cell injections in tumor-bearing mice is warranted. Nevertheless, we hypothesize that CAR T-cell products with improved persistence will ultimately lead to superior therapeutic efficacy.

Here, we leveraged our previously described DNMT3A-KO strategy to boost T-cell persistence.^40^ We hypothesize that combining the highly efficient killer CAR T-cell product (RASA2-KO) with our ‘persister’ CAR T-cell product (DNMT3A-KO) at a 1:1 ratio would achieve rapid initial tumor debulking followed by durable surveillance of residual tumor cells. Intriguingly, only the co-infusion of RASA2-KO and Ctrl-KO CAR T-cells significantly augmented B7-H3 CAR T-cell responses in our DMG model. These findings suggest that heterogeneous CAR T-cell populations may offer distinct therapeutic advantages. However, the unexpected lack of synergy between RASA2-KO and DNMT3A-KO populations warrants further mechanistic investigation. One possibility is that the increased Th1 response in RASA2-KO T cells might boost Ctrl-KO T cell functionality, resulting in a positive synergy.^55^ In line with this, our group has previously shown that DNMT3A-KO CAR T-cells have increased expression of IL-10.^40^ It is possible that IL-10 build-up in the tumor microenvironment may negatively impact the effect of RASA2-KO CAR T-cells; however, this must be experimentally tested.

Finally, our results revealed that RASA2-KO improves the dynamics of cytoskeletal remodeling, leading to not only improved IS formation but also increased CAR T-cell mobility. This allowed the CAR T-cells to be faster, more potent killers. While many published works have focused on improving CAR T-cell movement in terms of tumor homing/migration,^56^ our data show that CAR T-cell movement per se is also important. This is particularly impactful for tumors like DMG, which grow diffusely and within healthy neural networks, requiring CAR T-cells to travel to capture malignant cells and mount a meaningful anti-tumor response. While more in-depth analyses are needed, we propose that cytoskeleton remodeling and T cell mobility are critical therapeutically actionable targets for future CAR T-cell engineering strategies.

In summary, we have demonstrated that inefficient IS formation is a distinct feature of DMGs compared with other brain tumor types and can be restored through genetic editing of the CAR T-cells. Overall, our study underscores the importance of investigating tumor-specific resistance mechanisms to inform the development of disease-tailored genetic engineering strategies that maximize the potency and durability of CAR T-cell therapy.

## Materials and methods

### Cell lines

The following cell lines were purchased from American Type Culture Collection (ATCC, Manassas, VA): 293T (female fetus), U87-MG (adult male), D341 (pediatric male), D283 (pediatric male), DAOY (pediatric male), D425 (pediatric female). DMG and HGG cell lines (SJ-DIPGX7c (pediatric female), SJ-DIPGX9c (pediatric female), SJ-DIPGX29c (pediatric female), SJ-DIPGX37c (pediatric male), HSJD-DIPG007 (pediatric male), and SJ-HGGX42c (pediatric male)) were kindly provided by Dr. Suzanne J. Baker (St. Jude Children’s Research Hospital). The MB cell line (HDMB03^57^ (originally from Dr. Till Milde) (pediatric male)) was kindly provided by Dr. Martine F. Roussel (St. Jude Children’s Research Hospital). Ependymoma cell lines (ST1 (pediatric female), ST2 (pediatric female), 1425 (pediatric male) were kindly provided by Dr. Stephen Mack (St. Jude Children’s Research Hospital). All cell lines were authenticated using the ATCC’s or Hartwell Center (St. Jude Children’s Research Hospital) human STR profiling cell authentication service and routinely checked for Mycoplasma by the MycoAlert Mycoplasma Detection Kit (Lonza; LT07-318).

### Cell line cultures

U87-MG and DAOY cell lines were maintained in Eagle's Minimum Essential Medium (EMEM) media (ATCC; 30-2003). 293T was maintained in Dulbecco’s Modified Eagle Medium (DMEM) (GE Life Sciences; SH30081.01). All culture media listed above were supplemented with 10% Fetal Bovine Serum (FBS) (GE Life Sciences; SH30071.03) and GlutaMAX (2 mmol/L) (Gibco; 35050061). DMG and HGG cell lines were cultured in Tumor Stem Media as described in.^58^ MB cell lines were cultured in Neurobasal media (Thermofisher; 21103049) supplemented with 2 mmol/L GlutaMAX, 1X B27 (Thermofisher; 12587010), 0.00004% Heparin (Stemcell technologies; 7980), 20 ng/mL EGF (Peprotech; AF-100-15), and 20 ng/mL FGF-b (Peprotech; AF-100-15). Ependymomas (EPN) cell lines were cultured in Neurobasal media supplemented with 1 mM Sodium Pyruvate (Thermofisher; 11360070), 2 mmol/L GlutaMAX, 1X B27, 1X N2 (Thermofisher; 17502048), 10 ng/mL EGF, and 10 ng/mL FGF-b. All cell lines were grown in humidified incubators at 37°C and 5% CO_2_. U87-MG cells were modified to express GFP-firefly luciferase (GFP.ffluc) by retroviral transduction as described.^59^ SJ-DIPGX7 and HSJD-DIPG007 cells were modified to express YFP-firefly luciferase (YFP.ffluc) by lentiviral transduction. DMG, HGG, and EPN cell lines were grown in geltrex-coated (1% Geltrex (Thermofisher; A1413202) in DMEM/F12 HEPES media (Thermofisher; 11330057)) flasks.

### Primary human T cell culture and activation

Human peripheral blood mononuclear cells (PBMCs) were isolated from deidentified elutriation chambers of leukapheresis products obtained from St. Jude Children’s Research Hospital’s donor center. PBMCs were isolated using Lymphoprep (Abbott Laboratories; 07811) gradient centrifugation method. On day 0, PBMCs were stimulated on non-tissue culture-treated 24-well plates (Corning; 3738), which were precoated with CD3 and CD28 antibodies (αCD3/αCD28; Miltenyi Biotec; 130-093-337 (CD3: OKT3) and 130-093-386 (CD28: 15E8)). rhIL-7 (10 ng/mL; PeproTech; 200-7) and rhIL-15 (5 ng/mL; PeproTech; 200-15) were added to cultures on day 1. T cells were maintained in RPMI-1640 supplemented with 10% FBS, GlutaMAX (2 mmol/L), and rhIL-7 (10 ng/mL), rhIL-15 (5 ng/mL) cytokines were added every 3–4 days.

### Constructs and sequences

Generation of the B7-H3-CAR and IL13Rα2-CAR viral vectors has been previously described.^13 27^ The Delta-CAR, which served as a control CAR, was generated by deleting the CD28.CD3ζ signaling domain from the B7-H3-CAR. The sequence of all cloned constructs was confirmed by sequencing performed by Hartwell Center DNA Sequencing Core at St. Jude Children’s Research Hospital with Big Dye Terminator (V.3.1) Chemistry on Applied Biosystems 3730XL DNA Analyzers (Thermo Fisher Scientific; 4337454).

### Retrovirus production and transduction

The generation of the RD114-pseudotyped retroviral particles has been previously described.^59^ Briefly, retroviral particles were generated by transient transfection of 293Vec-RD114 cells which were seeded at 1–2 million cells per 10 cm^2^ dish 2 days before transfection and cultured in DMEM with 10% FBS. Cells were transfected with the CAR retroviral plasmid using the GeneJuice transfection reagent per manufacturer’s protocol (Novagen; 70967). 48 hours viral supernatants were collected, spun down at 400 g for 5 mins and filtered using 0.45 µm filter to remove the cell debris. Viral supernatants were then snap-frozen and stored at −80°C until further use.

For T cell transduction, 500 μL of viral supernatants were spun down on RetroNectin (Clontech; T100B) coated 24-well non-tissue culture plate at 2000 g for 90 min. After the spin, viral supernatants were removed and activated T cells (day 2) were plated at 2.5×10^5^ cells/well in 2 mL of RPMI 1640 supplemented with 10% FBS, 1% GlutaMAX, 10 ng/mL IL-7, and 5 ng/mL IL-15 T cell media (T cell media). 72 hours later, CAR transduced T cells were transferred from the RetroNectin-coated plate to a tissue culture plate and expanded in T cell media. CAR expression was determined 4 to 5 days post-transduction.

### CRISPR-Cas9 knock-out genetic editing

Activated T cells were electroporated with Streptococcus pyogenes Cas9-sgRNA ribonucleoproteins (RNP) complexes targeting RASA2 or AAVS1 (Ctrl), and 24 hours later cells were transduced on RetroNectin-coated plates. RNPs were precomplexed at an sgRNA:Cas9 ratio of 4.5:1, prepared by adding 3 µL of 60 µM sgRNA (Synthego) to 1 µL of 40 µM Cas9 (MacroLab, University of California, Berkeley) and frozen for later use (Guide RNA sequences: AAVS1: GGGAACCCAGCGAGTGAAGA; RASA2: AGGATCGACTTGTGGAACAA. For gene editing, 1×10e6 T cells were resuspended in 17 L of P3 Primary cell buffer (Lonza; V4XP-3032) and added to 3 µL of RNP. A total of 20 μL of cells plus RNPs were nucleofected by using EH-115 program in Lonza 4D-nucleofector (Lonza). One electroporation reaction was collected into one well of a 48-well tissue culture-treated plate containing RPMI 1640 supplemented with 20% FBS, GlutaMAX, IL-7 (10 ng/mL), and IL-15 (5 ng/mL) for 72 hours for recovery. After recovery, the medium was switched to RPMI 1640, containing 10% FBS and GlutaMAX. The cells were then expanded for 10 to 12 days with IL-7 and IL-15 added every 3 to 4 days at the same concentrations indicated above.

### Flow cytometry

All flow cytometry data were acquired with a BD FACSCanto II instrument and analyzed using FlowJo software (FlowJo, Ashland, OR). Samples were washed with and stained in Dulbecco-PBS (DPBS) (Lonza, Basel, Switzerland) with 1% FBS (GE Life Sciences; SH30071.03). For all samples, matched isotypes or known negative controls, for example, NT T cells, served as gating controls. Invitrogen LIVE/DEAD Fixable Aqua Dead Cell Stain kit (Fisher Scientific; L34957) was used as a viability dye. For B7-H3, ICAM-1, CD58, Galectin-9, and PD-L1 expression, cells were stained with anti-human CD276 AlexaFluor647 (R&D; FAB1027R), anti-CD54-APC (Biolegend; 353112), anti-CD58-PE (Biolegend; 330905), anti-Galectin9-PE (Biolegend; 348905), and anti-CD274-PE (Biolegend; 329705), respectively; at 1:100 dilution. Cells were washed twice and then resuspended for flow cytometry analysis. The B7H3-CAR and Delta-CAR were detected with G4SLinker-AlexaFluor647 (Cell signaling; 69 782L). T cell phenotype was assessed with: CD8-PerCP (Biolegend; 344708), CD45RA-APC (Biolegend; 304112), CCR7-FITC (Biolegend; 353216), CD4-PE/Cy7 (Biolegend; 344612, CD69-BV650 (BD biosciences; 563835), CD276-Alexafluor647 (R&D; FAB1027R-100UG), IL13Ra2-PE (Biolegend; 354403). All staining was performed for 15 min at room temperature in the dark.

For B7-H3 and IL13Rα2 quantification, Quantum Alexafluor 647 and R-PE MESF kits (Bangs Lab; 647A and 827A) were used, following manufacturer’s recommendations.

### Western blot

For RASA2-KO efficiency immunoblotting, gene-edited CAR T-cells were pelleted at 400 g for 5 min at 4°C. Each pellet was resuspended in Pierce RIPA buffer (Thermofisher; 89900) supplemented with protease phosphatase inhibitor cocktail (Cell Signaling; 5872S), and incubated at 4°C for 30 min. Cell lysates were centrifuged at 10 000 g for 10 min at 4°C and supernatant was collected. Protein concentration was determined using Pierce BCA protein assay (Thermofisher; 23255). 20 μg of protein were loaded onto 10% tris-glycine SDS gels (Bio-Rad), followed by transfer to a nitrocellulose membrane using the Biorad transfer system. The membrane was washed three times in Tris-buffered saline with Tween (TBST) (TBS+0.1% Tween) and blocked in 5% milk-TBST buffer for 1 hour at room temperature. Primary antibodies (RASA2 (GAP1m): Novus; NBP1-89794, 1:1000 dilution; GAPDH-HRP: Santa Cruz biotechnology; sc-47724, 1:10 000 dilution) were diluted in 1% milk TBST, added to the membrane, and incubated at 4°C overnight. Membranes were washed three times in TBST and incubated with secondary antibodies (anti-Rabbit-HRP, Cell Signaling; 7074; anti-Mouse-HRP, Cell signaling; 7076) diluted in 1% milk TBST for 1 hour at room temperature. Membranes were washed three times in TBST. Membranes were incubated with Clarity Western ECl substrate (Bio rad; 1705060) and imaged on the Odyssey Fc Imaging system (LI-COR Biosciences).

### IncuCyte killing assay

For IncuCyte killing assay, 1×10^6^ tumor cells were labeled with CellTracker Red CMTPX (1:1000) (Invitrogen; C34552) for 30 min and then washed and seeded onto geltrex coated 24-well plate (DMEM F-12 (Thermofisher; 11330057) +1% Geltrex LDEV-Free, hESC qualified (Thermofisher; A1413202)) for 4 hours at 37°C 5%CO_2_. 2.5×10^5^ Ctrl or CAR T-cells were added, and plates were placed in the IncuCyte S3 Live-Cell Analysis System where real-time images of tumor cells (RFP channel) were captured every hour for 96 hours. Cell viability of tumor cells was assessed as total RFP area (µm^2^) per image using IncuCyte S3 Software (Essen Bioscience; version 2022A). Cytotoxicity control was performed by adding 200 µL of 10% Saponin (Sigma-Aldrich; 47036-50 G-F) to tumor cells after 20 hours of acquisition.

### Repeated stimulation assay

Delta and B7-H3-CAR T-cells were co-cultured with tumor cells at an E:T ratio of 2:1. 7 days later, T cells were counted and replated with fresh tumor cells at the same 2:1 ratio. The T cells continued to be counted and stimulated with fresh tumor cells on a weekly basis until the T cells stopped killing tumor cells or fail to expand.

### Cytokine production

At 24 hours post-first stimulation, culture supernatants were collected, and cytokine production was assessed by a 13-plex human cytokine quantification kit (Millipore Sigma; HCYTOMAG-60K). Analysis was performed using a Luminex FlexMap 3D instrument and software (Luminex).

### Cytotoxicity assay

A CellTiter 96 AQueous One Solution Cell Proliferation Assay (Promega; G3580) was used to assess CAR T cell cytotoxicity as previously described.^60^ Briefly, tumor cells were incubated with varying amounts of CAR T cells to assess cytotoxicity at a range of E:T ratios. 24 hours later, the media and T cells were removed, and the remaining tumor cells were quantified with the CellTiter 96 AQueous One Solution Reagent containing a tetrazolium compound (3-(4,5-dimethylthiazol-2-yl)-5-(3-carboxymethoxyphenyl)-2-(4-sulfophenyl)-2H-tetrazolium, inner salt; MTS) and an electron coupling reagent (phenazine ethosulfate; PES). Media-only and tumor-only conditions served as controls to assess percent cytotoxicity. Absorbance was measured at 492 nm by using an Infinite200 Pro MPlex plate reader (Tecan, Männedorf, Switzerland). The percentage of live tumor cells was calculated using the following formula: [(Absorbance of sample−Absorbance of media only)/(Absorbance of tumor only-Absorbance of media only)]×100.

### Live cell imaging

1.5×10^5^ tumor cells were seeded onto geltrex coated μ-slide 8-well chambers (Ibidi; 80807) and incubated overnight at 37°C and 5% CO2. Tumor cells were labeled with CellTracker Red CMTPX (1:1000) (Invitrogen; C34552) for 30 min and then washed and maintained in culture media until image acquisition. 2×10^6^ CAR T cells were resuspended in 1 mL of PBS and labeled with CAL520AM (1:500) (ATTbioquest; 21130) and CellTrace Violet (1:1000) (Thermofisher; C34557) for 1 hour and then washed and maintained in T cell media until image acquisition. At the time of image acquisition, 1×10^5^ CAR T-cells were added to each well preloaded with tumor cells, and the image acquisition was initiated once T cells were detected in the visual field. Images were acquired in a spinning disc confocal microscope (Zeiss Axio Observer with CSU-X spinning disc), using a 63X objective. The acquisition parameters were a 4D image (60 min of acquisition with 1 min of frame, and 20 µm of height with a Z-step of 2 µm, at 37°C and 5% CO_2_).

For CAR T-cell activation onto immobilized antigen, antigen-coated 35 mm glass-bottom dishes (WPI; FD35-100) were prepared by coating with 0.5 µg/mL of rhIL13Ra2 (R&D Systems; 213-ILB-025/CF), αCD3/αCD28 (Miltenyi Biotec; 130-093-337 (CD3: OKT3) and 130-093-386 (CD28: 15E8)), or 0.01% PLL (Sigma-Aldrich; P4707) overnight at 4°C. Then, the coated dishes were washed with PBS and filled with media until CAR T cell seeding. Next, 2.5×10^5^ CAR T cells probed with CAL520AM, SPY650-FastAct (1:1000; Cytoskeleton; CY-SC505), and SPY555-Tubulin (1:1000; Cytoskeleton; CY-SC203), were plated onto the precoated dishes mounted in a total TIRFM (Zeiss Axio Observer 7 with 3i Vector 3 TIRF module). Microscope settings were adjusted prior to image acquisition by using a CAR T cell pre-seeded dish. The acquisition parameters were: 45 min of acquisition with 20 s of frame at 37°C and 5% CO_2_.

The processing and analysis were performed with FIJI (ImageJ) software.^61^ Cell tracking,^62^ Ca^2+^ flux, and synaptic size were performed using the Trackmate plugin as described in.^63^ All tumor and CAR T-cell interactions were recorded over time, and Ca^2+^ flux was measured as the maximum fluorescence emitted by CAL520 signal which was normalized by its value before the first peak of calcium influx on tumor interaction. Total Ca^2+^ flux was quantified as the area under the curve of Ca^2+^ flux registry until 30 min of interaction. For synaptic size quantification, the area of the intersection of independently segmented T cells and tumor cells images was used. For TIRFM image analysis, the synaptic area was measured by segmenting the actin cytoskeleton image and measuring its respective size over time. For calcium flux, F-actin, CAR, and MTOC recruitment to the synaptic interface, the mean fluorescence was measured over time. To evaluate the speed of response, a linear regression was calculated within the first minute of activation, using the slope of the regression as an indicator of speed. To calculate the timing of the MTOC recruitment to the synaptic interface, the first derivative was calculated and plotted, and the first peak of the curve was considered as the time in which the MTOC was recruited to the synapse.

### T cell migration in brain slices

To prepare brain slices, brains were extracted from euthanized FVB/NJ mice. And slices were prepared by using a Vibratome (Lafayette Instruments; 7000SMZ-2) for cut 300 µm thick coronal brain slices. Brain slices were stored temporarily in artificial CSF (aCSF; DI water base, 125 mM NaCl, 25.0 mM glucose, 25.0 mM NaHCO₃, 1.25 mM NaH₂PO₄, 1.0 mM MgCl₂, 1.5 mM CaCl₂) at 4°C.^64^ Prior to imaging, brain slices were labeled with Isolectin GS-IB_4_-AlexaFluor-568 (ThermoFisher; I21412) at a final concentration of 4 µg/mL for blood vasculature identification.

$0.2-0.4\times 10^{6}$ labeled (CellTracker green CMFDA; Thermofisher; C7025) T cells were added to the glass-bottom region of a glass-bottom dish (MatTek; P35G-0-20-C) in 150 µL complete RPMI and co-cultured them with two brain slices for 3–4 hours at 37°C and 5% CO₂. Before imaging, brain slices were washed and resuspended in 1 mL of complete RPMI. To prevent movement during imaging, a tissue culture anchor (Warner Instruments; SHD 40-10;) was placed on top of the slice.

Brain slices were imaged on an inverted Zeiss LSM 7 Live confocal line-scanning microscope at 37°C in 5% CO₂ with a frame interval of 2 min for 3–9 hours. Images were collected with 10×or 20×objective lenses (Plan-ApoChromat 10X, 0.45 NA, Zeiss, or Plan-ApoChromat 20x, 0.8 NA, Zeiss). Two independent acquisitions were performed for each donor. In each dish, we selected 12 positions distributed across different regions and depths of the brain slice (24 positions total per imaging loop). Each xy-plane was acquired together with 3 z-stacks (total depth 20 to 30 µm). To generate 2D data, maximum projection along the z-axis was applied, and if drift was noticed, a custom MATLAB script based on *imregtform* with translation or similarity transforms was applied.

TrackMate^36^ (Fiji) on Python environment was used to identify and track cells. The Laplacian of Gaussian (LoG) detector with an estimated spot diameter of 14 µm was used to detect cells. To limit low-contrast or out-of-field cells, spots were filtered out with a contrast less than 0.15. The LAP tracker algorithm with gap closing was enabled, and both track splitting and merging were disabled. A maximum identifiable distance of 25 µm per minute (50 µm per frame), a maximum gap of 2 frames, and a maximum per-frame gap-closing distance of 62.5 µm were used. Tracks that were at the edge of the brain slice and too close to the glass bottom were excluded. Tracking data was imported into MATLAB to perform subsequent analyses.

For each track, simple one-lag statistics (median step size or frame-to-frame displacement magnitude), and simple directionality statistics (linearity of forward progression and confinement ratio) were calculated. Next, the MSD was calculated by using the overlapping time interval method and fit three candidate random walk models (constrained random walk, Brownian walk, and persistent random walk; Eq. 1-3) to the initial 40% of the MSD vs lag (τ) curve (described in detail below). We used the Corrected AICc (Eq. 4) to assign each track to one of the three random walk models and then estimated the fraction of these walk types in the control- and RASA2-KO conditions.

**Table:** 

Constrained random walk	(1) $MSD\left(\tau \right)=R^{2}\left(1-e^{\frac{-4D_{c}\tau }{R^{2}}}\right)$
Brownian walk	(2) MSD(τ)=4Dτ D: random motility coefficient
Persistent random walk	(3) $MSD\left(\tau \right)=2v^{2}\tau_{p}^{2}\left(\tau /\tau_{p}-\left(1-e^{-\frac{\tau }{\tau_{p}}}\right)\right)$
Generalized power-law walk	(4) $MSD\left(\tau \right)=A\tau^{\alpha }$ α: diffusion exponent
Corrected Akaike information criterion (**$AIC_{c}$**)	(5) $$AIC_{c}=n\ln\left(\frac{RSS}{n}\right)+2k+\frac{2k\left(k+1\right)}{n-k-1}$$

$MSD\left(\tau \right)=\langle ∥r\left(t+\tau \right)-r\left(t\right){∥}^{2}\rangle$is the mean square displacement (with $r\left(t\right)$ as position) and was computed using overlapping time intervals. A common null model for cell migration is an isotropic Brownian Walk (normal diffusion; Eq. 2), with step lengths (frame-to-frame displacement magnitude) following a Rayleigh (2D) or Maxwell (3D) distribution. In this simple model of diffusion, $MSD\left(\tau \right)$ scales linearly with τ for all τ. On a log scale, this model is a straight line with a slope=1 (the diffusion exponent α=1 in the generalized power-law walk (Eq. 4)). On the other hand, the constrained random walk (Eq. 1), which behaves like Brownian walk with a motility coefficient ($D_{c}$) at low τ, but appears constrained (confined) at high τ. In this model, the early MSD scales linearly with τ(slope=1 in the log(MSD)vs $\log\left(\tau \right)$ plot), but it eventually saturates (slope=0). Averaged over the full lag range, their combination yields slope<1 (α§amp;lt;1 in Eq. 4; an apparent “subdiffusion”). Biologically, this can occur when T cells engage with cells in the brain parenchyma or become locally arrested. Finally, the persistent random walk model (Eq. 3), usually employed for T-cells and other rapidly migrating cells, cells maintain a direction at a speed v (persistence speed) for a short time (persistence time P) before turning, while becoming isotropic on long τ. In the log(MSD)vs $\log\left(\tau \right)$ plot, this appears as two straight lines with slope=2 at low τ (ballistic) and slope=1 at high τ (diffusive), which when fitted with a single line (Eq. 4) would typically yield an average α§amp;gt;1.

Next, to estimate which model best describes a given track, we fit these three models to each cell’s MSD curve using *fmincon* in MATLAB and computed the RSS. Since the number of free parameters (k) varied between these models, we used an information criterion to rank these fits, while noting that MSD points across lags are correlated when overlapping intervals are used. Specifically, we used corrected Akaike information criteria to account for the small track size (n’; Eq. 5).

### Fixed cell imaging

#### Antigen-coated coverslip preparation and CAR T cell activation

Antigen-coated coverslips were prepared using N1 coverslips (Thermo Fisher Scientific; 12-545-80P), which were coated with 0.5 µg/mL of rhB7H3 (R&D Systems; 3035-A2-100) or 0.01% PLL (Sigma-Aldrich; P4707) overnight at 4°C. Then, they were washed with PBS and filled with media until CAR T cell seeding. Next, 2×10^5^ CAR T cells were plated onto the pre-coated coverslips for 30 min in a cell culture incubator (37°C/5% CO2).

#### CAR T-cell activation with tumor cells

1×10^5^ tumor cells were seeded onto PLL-coated coverslips and cultured for 1 hour in a cell culture incubator (37°C/5% CO2). Next, 1×10^5^ CAR T cells were plated onto tumor-seeded coverslips for 30 min in a cell culture incubator (37°C/5% CO2).

#### Fixation and staining

After activation, coverslips were washed with cold PBS and fixed with 4% paraformaldehyde (PFA, Electron Microscopy Sciences, 15710) for 10 min at room temperature. Fixed cells were washed twice with PBS, and the remaining PFA was inactivated with blocking buffer (PBS-2% BSA (Sigma-Aldrich; A9418) and 1.5 M glycine (Sigma-Aldrich; G8898)) for 10 min at room temperature. Cells were permeabilized by adding permeabilization buffer (PBS, 0.2% BSA and 0.05% saponin; Sigma-Aldrich; 47036) for 20 min at room temperature. Cells were washed twice with a permeabilization buffer before primary antibody incubation and diluted in the permeabilization buffer, following the manufacturer’s instructions. All the primary antibodies were incubated at 4°C overnight. Cells were washed with permeabilization buffer and incubated with secondary antibodies for 2 hours at room temperature. Finally, cells were washed with permeabilization buffer and PBS before letting them dry for 1 hour at room temperature. Then, coverslips were mounted onto slides using Fluoromount (Thermo Fisher Scientific; 00-4958-02).

#### Antibodies and probes

Primary antibodies and probes with their dilutions are as follows: anti-human Lamp1 (1:50) (Abcam, ab25630); anti-human pZAP70 (1:50) (Cell Signaling Technology, 2701L); anti-human-CD45 (1:100) (Abcam; ab8216), anti-G4SLinker (1:100) (Cell Signaling; 71645), anti-human-γTub (1:100) (Abcam; ab11316), phalloidin-Alexa Fluor 647 (1:200) (Thermo Fisher Scientific, A22287). Secondary antibodies with their dilutions are as follows: anti-rabbit Alexa Fluor 488 (1:200) (Thermo Fisher Scientific, A32731) and anti-mouse Alexa Fluor 568 (1:200) (Thermo Fisher Scientific, A-11004).

#### Image acquisition and analysis

Images were acquired in a spinning disc confocal microscope (Zeiss Axio Observer with CSU-X spinning disc), and the processing and analysis were performed with Fiji (ImageJ) software. Single-cell images shown in the figures were cropped from a larger field. Image brightness and contrast were manually adjusted. To analyze lysosome and pZAP70 recruitment to the IS in antigen-coated coverslips activated CAR T-cells, cell borders were automatically delimited by using the segmented F-Actin channel, then pZAP70, F-Actin, and lysosome MFI were measured. To analyze lysosome, CAR, CD45, γ-Tub, and pZAP70 recruitment to the IS in CAR T cells interacting with tumor cells, T cells were segmented by using the Celltrace violet image, images were average Z-projected, and the center of mass was measured for each label, and the distance to the IS was calculated.

### Single-cell avidity assay

Tumor cells (SJ-DIPGX7C and U87-MG) were seeded into laminin-coated piezo chips from LUMICKS. Tumor cells adhered for 1 hour. T cells were labeled with CellTrace Far Red at 1:1000 dilution for 20 min at 37C 5% CO2. Piezo chips loaded with tumor cells were loaded onto the z-MOVI single-cell avidity analyzer. Labeled T cells were injected into the chip and allowed to incubate on tumor cells for 2 min. After this time, T cells were subjected to increasing the acoustic force ramp from 0 to 1000 pN over 2 min and 30 s. Individual cells were observed, and the exact force requirement for detachment was recorded based on the individual cell leaving the focal plane.

### Total RNA-seq gene expression analysis in tumor cells

Tumor cell RNA was extracted using the RNeasy plus mini kit (Qiagen; 74134). RNA samples were then sent for sequencing at the Hardwell Center (St. Jude). Sequencing quality was assessed by FASTQC, trimming by Trimgalore, and mapping/quantification by Kallisto using the Homo Sapiens Ensembl transcriptome V.96. Gene matrices were loaded into R by using Txi-import, and differential gene expression analyses were done by using DESeq2. GSEA was performed by using the RStudio package ClusterProfiler. Heatmaps were made by using the ComplexHeatmap package in RStudio.

Cell cycling (G2M and S phase), migration, and IS score gene set enrichment scores were obtained by using the Rstudio package uCell with Tirosh *et al* gene list,^65^ Gene ontology Migration pathway (GOBP_CELL_MIGRATION), and IS-related genes (Adhesion: CD58, ICAM1, ICAM2, ICAM3, ICAM4, ICAM5; Activation: CD40, CD70, CD80, CD86, ICOSLG, TNFSF14, TNFSF18, TNFSF4, TNFSF9; Inhibition: CD274, CD276, CEACAM1, IDO1, LGALS3, LGALS9, PDCD1LG2, PVR, VTCN1), respectively.

### CAR T cell isolation from brain tumors and total transcriptome analysis

NSG mice were implanted with 1e6 HSJD-DIPG007, as described above. 2e6 CAR T-cells (AAVS1 and RASA2-KO) were intracranially delivered after 7 days from tumor implantation. Mice were euthanized after 3 days from T cell injection, perfused with PBS, and brain dissected and processed by using the adult brain dissociation kit (Miltenyi; 130–107-677), following manufacturer’s instructions. Dissociated cells were subsequently filtered by using the CD3 Microbeads kit (Miltenyi; 130-097-043) for T cell enrichment. T cell RNA was extracted using the RNeasy Plus mini kit (Qiagen; 74134). RNA samples were then sent for sequencing at the Hardwell Center (St. Jude). Sequencing quality was assessed by FASTQC, trimming by Trimgalore, and mapping/quantification by Kallisto using the Homo Sapiens Ensembl transcriptome V.96. Gene matrices were loaded into R by using Txi-import, and differential gene expression analyses were done by using DESeq2. GSEA was performed by using the RStudio package ClusterProfiler. Transcription factor (TF) activity inference was made by using DecoupleR with the Univariate linear model (ulm). Statistically significant TFs that were differentially regulated between RASA2-KO and Ctrl-KO CAR T-cells were assessed by t-test. Heatmaps were made by using the ComplexHeatmap package in RStudio.

### scRNA-seq gene expression analysis

#### Sample preparation and sequencing

For single cell RNA sequencing, two sets of co-culture arrays were prepared: (1) 2.5×10^6^ Ctrl T cells were co-cultured with 2.5×10^6^ tumor cells (SJ-DIPGX7c, SJ-DIPGX9c, SJ-DIPGX37c, D425, ST1, and U87-MG) and (2) 1×10^6^ CAR T-cells were co-cultured with 4×10^6^ tumor cells (SJ-DIPGX7c, SJ-DIPGX9c, SJ-DIPGX37c, D425, ST1, and U87-MG). Both co-culture arrays were incubated at 37°C 5% CO_2_ for 16 hours. Co-cultures were enzymatically digested with Accutase (Thermofisher; A1110501) for 10 min at room temperature, and cells were filtered by using Miltenyi dead cell removal kit (Miltenyi; 130-090-101). Highly viable cells were recovered and prepared using the Fixation Kit (PARSE Bioscience) according to manufacturer ‘s protocol. After fixation, the cell count was determined by using a Neubauer hemocytometer. A total of 1.14×10^5^ cells from 12 coculture conditions were used, resulting in approximately 9.5×10^4^ cells per condition. These cells were subjected to the Single Cell Whole Transcriptomic kit v2 (PARSE Bioscience) for library construction. Libraries were subsequently sequenced using the Illumina NovaSeq 6000 platform, generating paired-end 200 bp reads.

### Data preprocessing of scRNA-seq

Fastq files from 10 sublibraries were demultiplexed into 12 samples using *split-pipe* pipeline (V.1.0.4p) from PARSE Bioscience. Human reference genome GRCh38/hg38 was used to map and quantify gene expression. Gene expression matrices were loaded into the R package Seurat (V.5) ^66^ for quality control and downstream analyses. We kept high-quality cells by removing cells that have more than 10% of mitochondrial content and less than 200 gene counts. Gene counts were then normalized and transformed with the NormalizeData function.

### Clustering and single-cell data analysis

High variable genes were obtained using the FindVariableFeatures function with default parameters. The top 2000 variable genes were selected as input for ScaleData, integration, and dimension reduction by IntegrateLayers (RPCA method) and RunUMAP functions. The top 15 principal components were used to construct nearest-neighbor graphs and identify clusters using the FindNeighbors functions of Seurat (V.5) R package.

Reference-based annotation with SingleR (V.2.0.0)^21^ R package was used to identify T cells from tumor cells, by using the Human Primary Cell atlas as reference from CellDex R package.^21^

T cells and tumor cells were segregated into their respective coculture condition, and intercellular inferred interaction probabilities between T cells and tumor cells were generated by the CellChat^22^ R package using default settings. CellChat infers communication networks by integrating gene expression with curated databases of ligand-receptor pairs and cofactors, assigning a communication probability score to potential interaction pathways. The aggregated score will define the total probability of interaction between two cells. T cell-weighted interaction probability with tumor cells was obtained for each coculture condition, plus their detailed matrix of interaction per pathway. The pathway interaction probability was subsequently normalized by its maximum value among the different coculture conditions.

Differentially expressed genes were performed using the findMarkers function in the Seurat package, and for GSEA, we used the Molecular Signature Database (MSigDB) by using the MSigDBr^67^ and ClusterProfiler^68^ packages in R. All terms with p<0.05 were considered significant and ranked by the number of genes identified in the group.

### Animals

Mice were euthanized when they met physical euthanasia criteria (tumor size, significant weight loss, signs of distress), or when recommended by St. Jude veterinary staff. For in vivo experiments, NSG mice were treated with CAR T cells generated from one to two different healthy donors. All experiments were performed using 10–12-week-old male NOD.Cg-Prkdc^scid^IL2Rg^tm1wjl^/SzJlnv (NSG) mice. Female mice were excluded from the study because they did not meet weight and age criteria required for survival surgeries established and approved by IACUC. Cages of mice were randomly assigned to treatment groups. The technicians injecting and imaging the mice were not blinded to the names of treatment groups, but they were unaware of their meaning or expected results. The bioluminescence-defined endpoint (total flux greater than 1×10^10^ photons/second) was selected to maximize animal welfare as larger tumor burdens were associated with clinical signs of pain and distress.

### Brain tumor model

U87-MG.GFP.ffluc, SJ-DIPGX7c.YFP.ffluc, HSJD-DIPG007.YFP.ffluc, and 1425.YFP.ffluc cells were implanted intracranially into the cortex of NOD.Cg-Prkdcscid Il2rgtm1Wjl/SzJ (NSG) mice. For intracranial implantation, mice were anesthetized and placed in a stereotactic rodent surgery platform. The scalp was cut using surgical scissors, and a cranial window was made in the skull, over the right cortical hemisphere, by using a dental drill. A sterile, cold Hamilton syringe containing 3X10^4^ U87-MG.GFP.ffluc, 1.5×10^5^ SJ-DIPGX7c.YFP.ffluc, 1×10^6^ HSJD-DIPG007.YFP.ffluc, or 1×10^6^ 1425.YFP.ffluc cells was suspended in 5 uL Matrigel (80% in DPBS) (Corning; 356234). Mice were placed in the stereotactic apparatus. The needle of the syringe was brought into contact with the surface of the brain, slowly inserted 3 mm into the cortex, raised 0.5 mm, and then the cells were injected over 1 min. The syringe was left in place for 30 s to prevent backflow. Wound clips were used to close the surgical site. 2×10^6^ B7-H3-CAR or 2×10^6^ IL13Rα2-CAR T cells were injected 7 (U87, SJ-DIPGX7c, and HSJD-DIPG007) or 135 (consistent tumor implantation for the 1425 ependymoma cell line) days later in the same location in 2 µL of PBS, respectively. Tumor burden was assessed weekly using bioluminescent in vivo imaging system (Perkin Elmer, IVIS) until the humane end point was reached, and mice were euthanized.

### Bioluminescence imaging

Mice were imaged by the St. Jude Center for In Vivo Imaging and Therapeutics. Mice were injected i.p. with 150 ng/kg of D-luciferin 5–10 min before imaging, anesthetized with isoflurane (1.5%–2% delivered in 100% O2 at 1 L/min), and imaged with Xenogen IVIS-200 imaging system (Xenogen, Alameda, CA). Quantification of tumor burden was performed using Living Image software (Perkin Elmer). A region of interest at the head was drawn around each mouse, and total flux was recorded in units of photons/second.

### Statistical analysis

Statistical analyses were performed using GraphPad Prism V.9.0 (Graphpad Software, La Jolla, California, USA). Statistical analyses were only performed when the number of replicates was at least three. For comparisons of two groups, a paired or unpaired t-test was used. Two-tailed t-tests were performed in all instances unless otherwise specified in the figure legend. For comparisons of three or more groups, a one- or two-way analysis of variance was used followed by either Dunnett’s, Sidak’s, or Tukey’s multiple comparisons test. Survival data were analyzed using a log-rank (Mantel-Cox) test. For all experiments, p values less than 0.05 were considered significant.

## Supplementary material

10.1136/jitc-2025-013134 online supplemental figure 1

10.1136/jitc-2025-013134 online supplemental figure 2

10.1136/jitc-2025-013134 online supplemental figure 3

10.1136/jitc-2025-013134 online supplemental figure 4

10.1136/jitc-2025-013134 online supplemental figure 5

10.1136/jitc-2025-013134 online supplemental figure 6

10.1136/jitc-2025-013134 online supplemental figure 7

10.1136/jitc-2025-013134 online supplemental figure 8

10.1136/jitc-2025-013134 online supplemental figure 9

10.1136/jitc-2025-013134 online supplemental figure 10

10.1136/jitc-2025-013134 online supplemental figure 11

10.1136/jitc-2025-013134 online supplemental figure 12

10.1136/jitc-2025-013134 online supplemental figure 13

10.1136/jitc-2025-013134 online supplemental figure 14

10.1136/jitc-2025-013134 online supplemental figure 15

10.1136/jitc-2025-013134 online supplemental figure 16

10.1136/jitc-2025-013134 online supplemental figure 17

10.1136/jitc-2025-013134 online supplemental figure 18

10.1136/jitc-2025-013134 online supplemental figure 19

10.1136/jitc-2025-013134 online supplemental figure 20

10.1136/jitc-2025-013134 online supplemental file 1

## Data Availability

Data are available on reasonable request.
