# Supplementary material for: RASA2 deletion rescues immune synapse dysfunction, enhancing CAR T cell efficacy against DMGs
Source: J Immunother Cancer. 2026 Mar 30;14(3):e013134. doi: 10.1136/jitc-2025-013134 (PMC13052770; doi:10.1136/jitc-2025-013134)
Supplement: online supplemental figure 1 [file jitc-14-3-s001.pdf]

Fig. S1

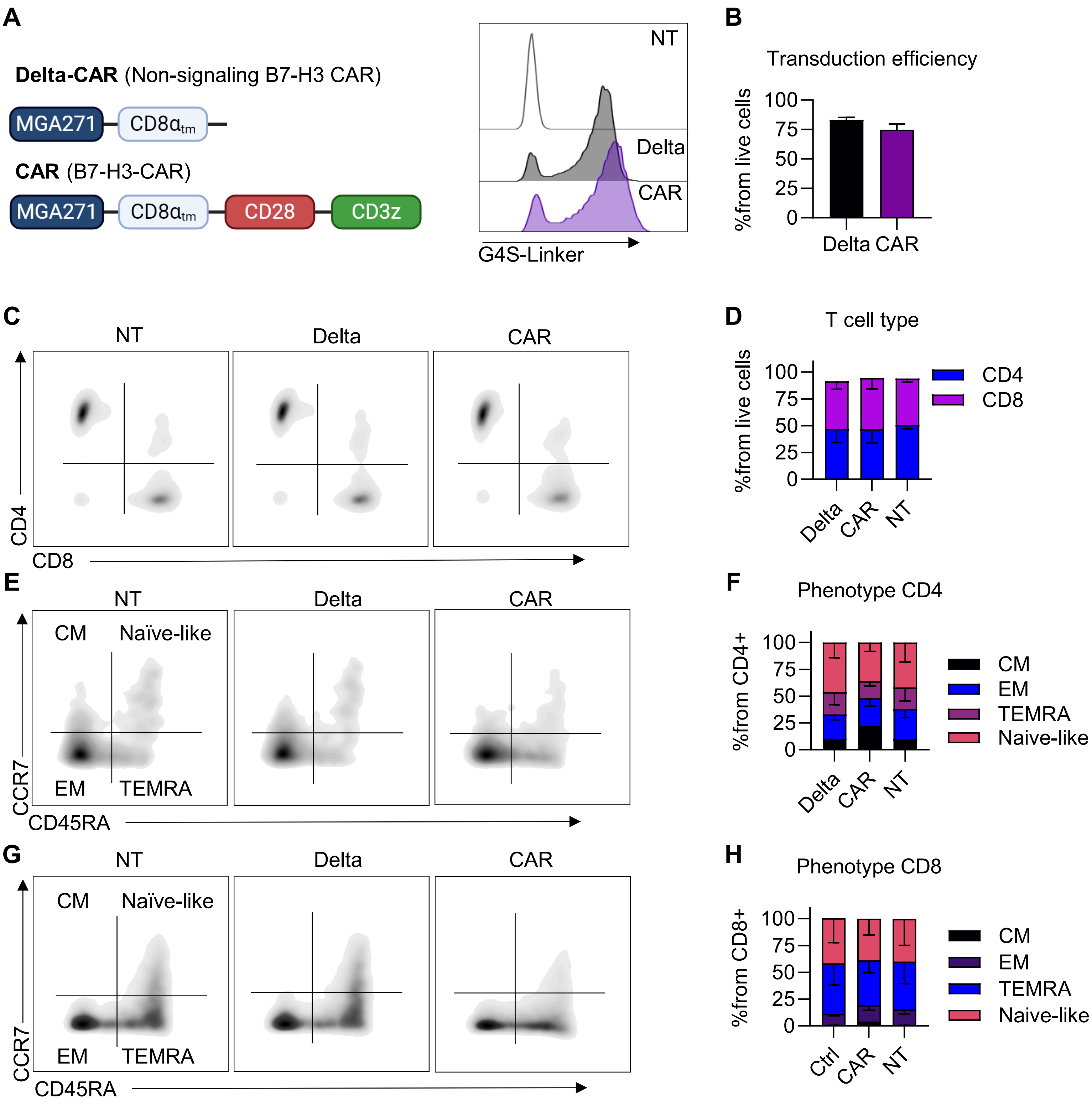

Fig. S1: B7-H3 CAR is efficiently transduced in T cells and does not alter their phenotype. (A), Chimeric antigen receptor schematics for Delta-CAR (used as a control CAR) and B7-H3-CAR constructs, and representative histogram plots of CAR expression (G4S-Linker) on T cells after 5 days of transduction, measured by flow cytometry, showing non-transduced (NT), Delta-CAR (Delta), and B7-H3-CAR (CAR) T-cells. (B) Quantification of transduction efficiency showed in (A) (N=6 T cell donors). (C) Representative density plots of CD4 and CD8 expression on T cells after transduction, compared to non-transduced T cells, measured by flow cytometry. (D) Quantification of CD4 and CD8 proportion in (C) (N=2-8 T cell donors). (E, G) Representative density plots of memory phenotype of CD4 and CD8 T cells, respectively, measured by flow cytometry. (F) and (H) Quantification of the memory phenotype in CD4 and CD8 T cells showed in (E) and (G) (N=4 T cell donors). Central Memory (CM; CCR7+CD45RA-), Effector memory (EM;CCR7-CD45RA-), Terminally differentiated (TEMRA; CCR7-CD45RA+), and Naïve-like (CCR7+CD45RA+).
