# Supplementary figures and images for: RASA2 deletion rescues immune synapse dysfunction, enhancing CAR T cell efficacy against DMGs
Source: J Immunother Cancer. 2026 Mar 30;14(3):e013134. doi: 10.1136/jitc-2025-013134 (PMC13052770; doi:10.1136/jitc-2025-013134)

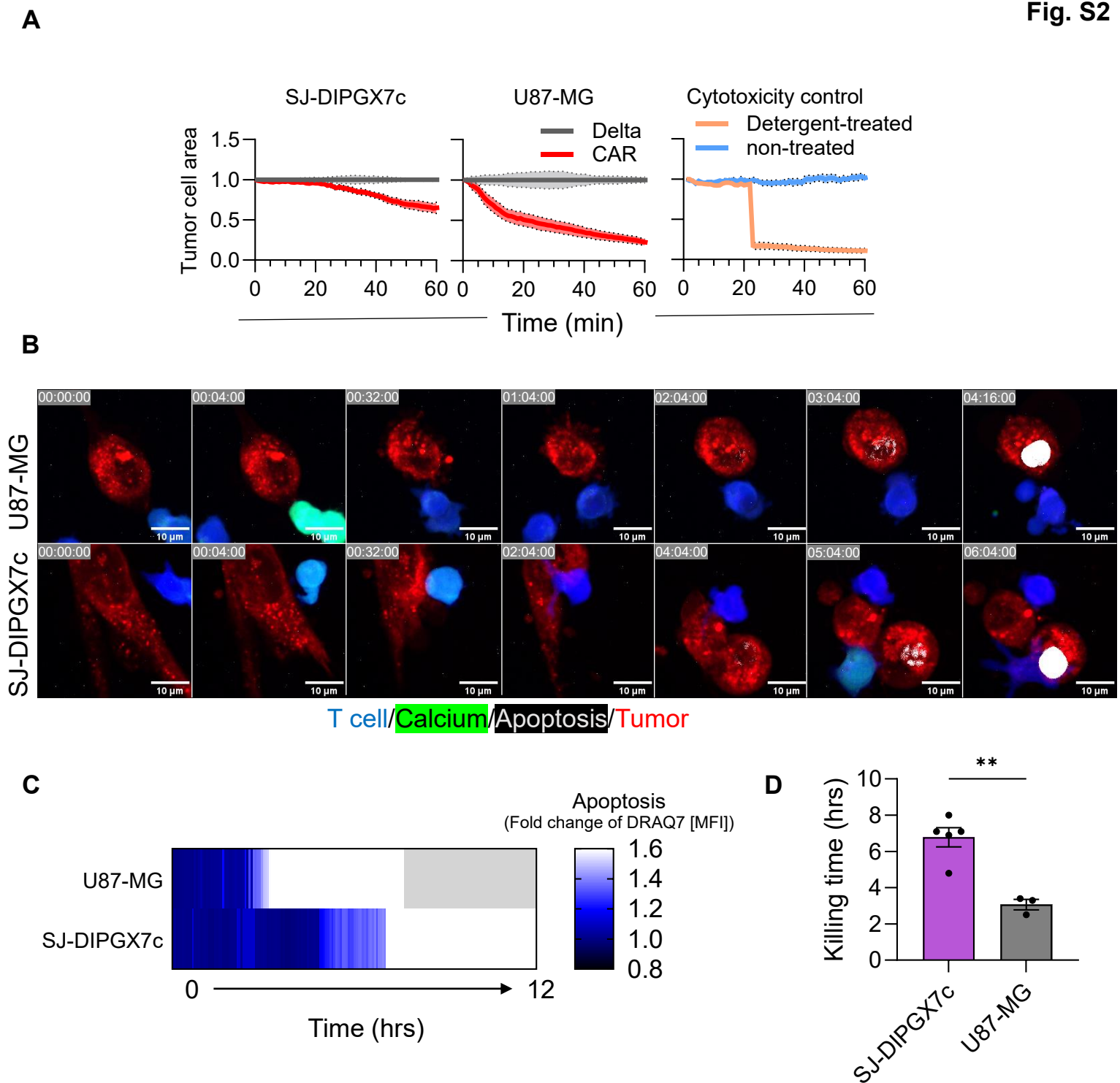

Supplement: online supplemental figure 2 [file jitc-14-3-s002.pdf]

**B**

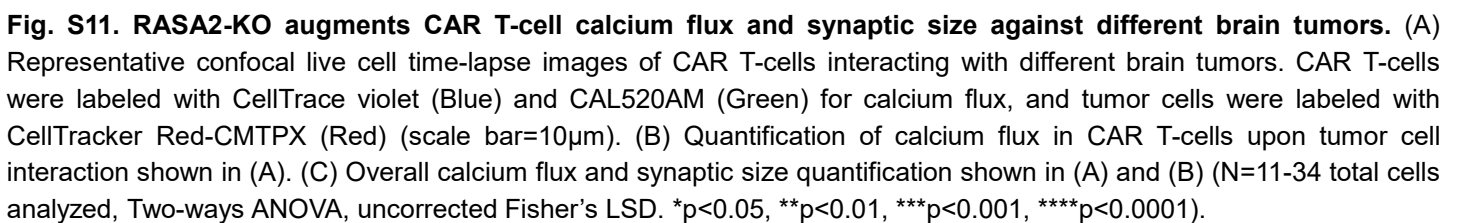

Supplement: online supplemental figure 11 [file jitc-14-3-s011.pdf]

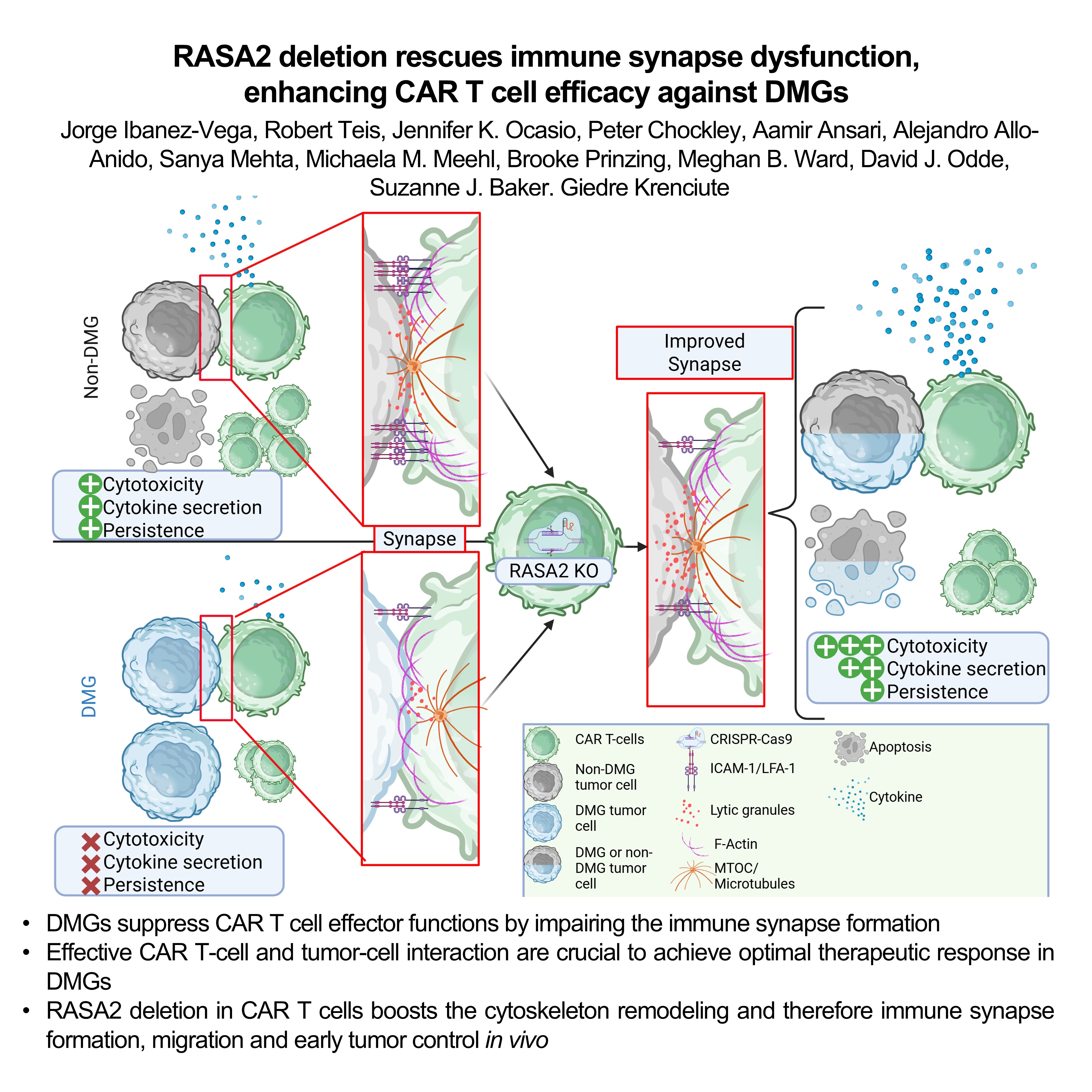

Supplement: online supplemental file 1 [file jitc-14-3-s021.jpg]
