# Supplementary material for: RASA2 deletion rescues immune synapse dysfunction, enhancing CAR T cell efficacy against DMGs
Source: J Immunother Cancer. 2026 Mar 30;14(3):e013134. doi: 10.1136/jitc-2025-013134 (PMC13052770; doi:10.1136/jitc-2025-013134)
Supplement: online supplemental figure 3 [file jitc-14-3-s003.pdf]

Fig. S3

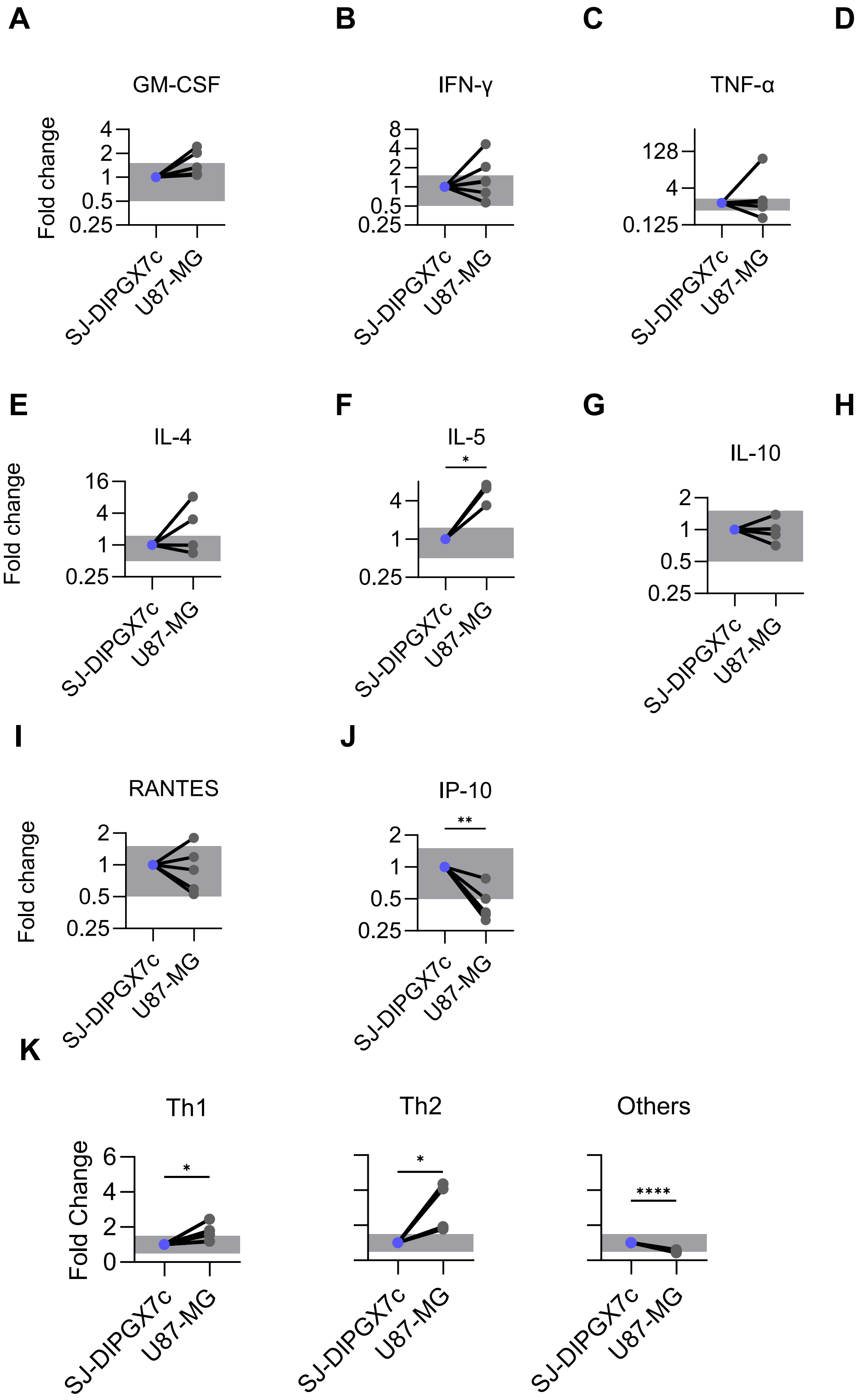

Fig. S3. Th1 cytokine secretion by CAR T-cells is increased in U87-MG compared to SJ-DIPGX7c. (A-J) Individual cytokine secretion measured by Milliplex cytokine quantification kit (GM-CSF, IFN-γ, TNF-α, IL-2, IL-4, IL-5, IL-10, IL-13, RANTES, and IP-10) of CAR T-cells after 24 hrs of co-culture with tumor cells (SJ-DIPGX7c and U87-MG), respectively (N=5-7 T cell donors, paired t-test. *$p<0.05$, **$p<0.01$). (K) Cytokine secretion aggregation by response type. Th1: GM-CSF, IFN-γ, TNF-α, IL-2; Th2: IL-4, IL-5, IL-10, IL-13; and Others: RANTES and IP-10 (N=5-7 T cell donors, paired t-test. *$p<0.05$, **$p<0.01$, $p<0.0001$****).
