# Supplementary material for: RASA2 deletion rescues immune synapse dysfunction, enhancing CAR T cell efficacy against DMGs
Source: J Immunother Cancer. 2026 Mar 30;14(3):e013134. doi: 10.1136/jitc-2025-013134 (PMC13052770; doi:10.1136/jitc-2025-013134)
Supplement: online supplemental figure 4 [file jitc-14-3-s004.pdf]

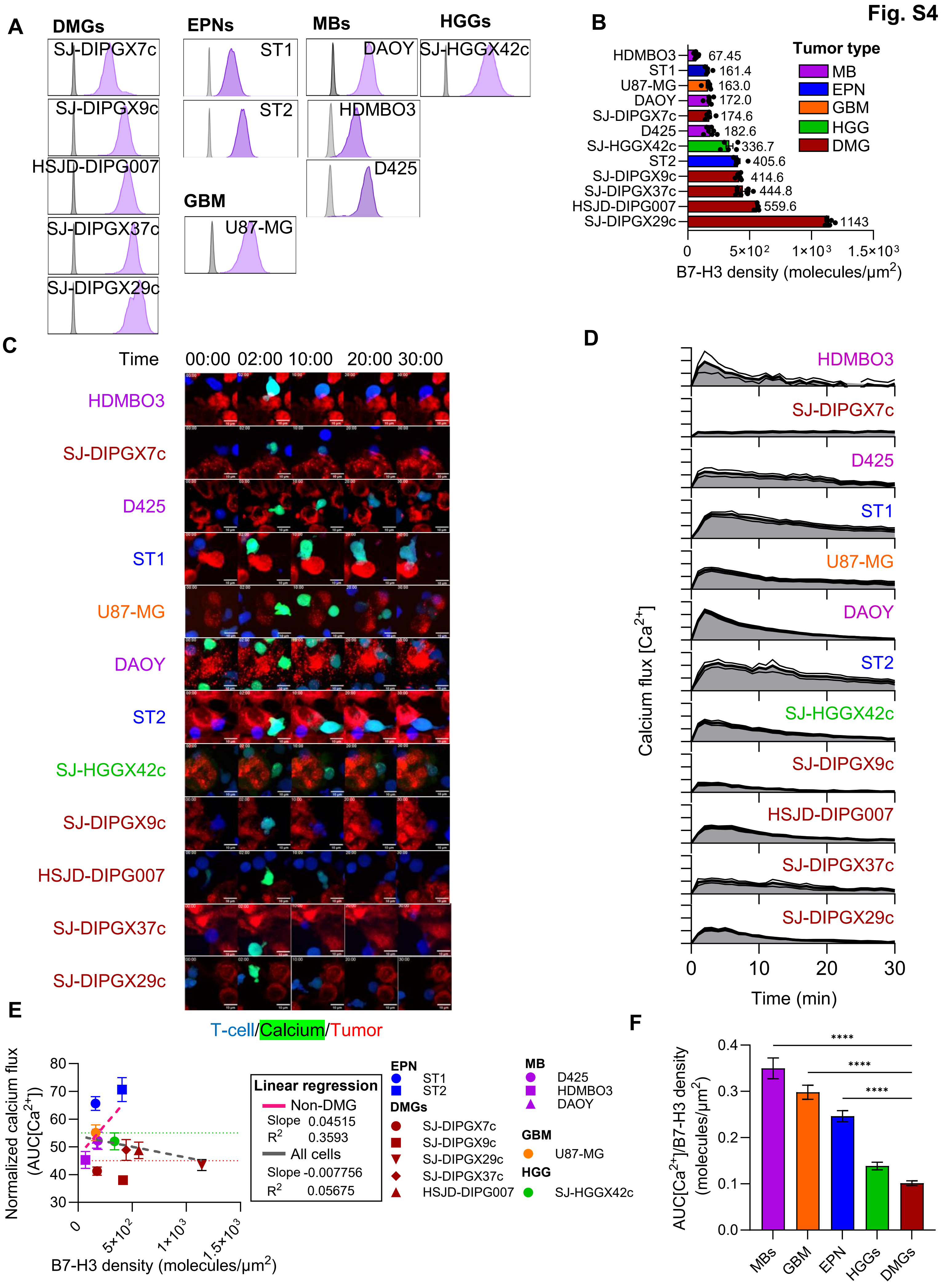

Fig. S4
A
DMGs
EPNs
MBs
HGGs
SJ-DIPGX7c
ST1
DAOY
SJ-HGGX42c
SJ-DIPGX9c
ST2
HDMBO3
HSJD-DIPG007
D425
GBM
SJ-DIPGX37c
U87-MG
SJ-DIPGX29c
B
Tumor type
MB
EPN
GBM
HGG
DMG
HDMBO3 67.45
ST1 161.4
U87-MG 163.0
DAOY 172.0
SJ-DIPGX7c 174.6
D425 182.6
SJ-HGGX42c 336.7
ST2 405.6
SJ-DIPGX9c 414.6
SJ-DIPGX37c 444.8
HSJD-DIPG007 559.6
SJ-DIPGX29c 1143
B7-H3 density (molecules/μm2)
C
Time 00:00 02:00 10:00 20:00 30:00
HDMBO3
SJ-DIPGX7c
D425
ST1
U87-MG
DAOY
ST2
SJ-HGGX42c
SJ-DIPGX9c
HSJD-DIPG007
SJ-DIPGX37c
SJ-DIPGX29c
T-cell/Calcium/Tumor
D
Calcium flux [Ca2+]
Time (min)
E
Normalized calcium flux (AUC[Ca2+])
B7-H3 density (molecules/μm2)
Linear regression
Non-DMG
Slope 0.04515
R2 0.3593
All cells
Slope -0.007756
R2 0.05675
EPN
ST1
ST2
DMGs
SJ-DIPGX7c
SJ-DIPGX9c
SJ-DIPGX29c
SJ-DIPGX37c
HSJD-DIPG007
MB
D425
HDMBO3
DAOY
GBM
U87-MG
HGG
SJ-HGGX42c
F
AUC[Ca2+]/B7-H3 density (molecules/μm2)
****
MBs
GBM
EPN
HGGs
DMGs

Fig. S4. Low calcium response in CAR T-cells is unique to DMGs and not other pediatric brain tumors. (A) and (B) Representative histogram plots for B7-H3 expression on different tumor cells (MBs [HDMBO3, D425, and DAOY], EPN [ST1 and ST2], DMGs [SJ-DIPGX7c, SJ-DIPGX9c. SJ-DIPGX29c, SJ-DIPGX37c, and HSJD-DIPG007], GBM [U87], and HGG [HGG42]) and their respective B7-H3 molecules per surface volume quantification (N=4-7 biological replicates). (C) Representative confocal live cell time-lapse images of CAR T-cells interacting with different brain tumors. CAR T-cells were labeled with CellTrace violet (Blue) and CAL520AM (Green) for calcium flux, and tumor cells were labeled with CellTracker Red-CMTPX (Red) (scale bar= 10 μm). (D) Quantification of calcium flux in CAR T-cells upon tumor cell interaction shown in (C) (N>20 cells analyzed for each tumor type). (E) Correlation plot between total calcium flux of CAR T-cells upon interaction with tumor cells and their respective antigen expression. (F) Bar plot illustrating calcium flux response upon tumor interaction, normalized by the mean antigen expression and aggregated into main tumor types (MBs [HDMBO3, D425, and DAOY], EPN [ST1 and ST2], DMGs [SJ-DIPGX7c, SJ-DIPGX9c. SJ-DIPGX29c, SJ-DIPGX37c, and HSJD-DIPG007], GBM [U87], and HGG [SJ-HGGX42c]) (N=21-206 cells, Ordinary one-way ANOVA, Tukey's multiple comparison test. *p<0.05,***p<0.001).
