# Supplementary material for: RASA2 deletion rescues immune synapse dysfunction, enhancing CAR T cell efficacy against DMGs
Source: J Immunother Cancer. 2026 Mar 30;14(3):e013134. doi: 10.1136/jitc-2025-013134 (PMC13052770; doi:10.1136/jitc-2025-013134)
Supplement: online supplemental figure 5 [file jitc-14-3-s005.pdf]

Fig. S5

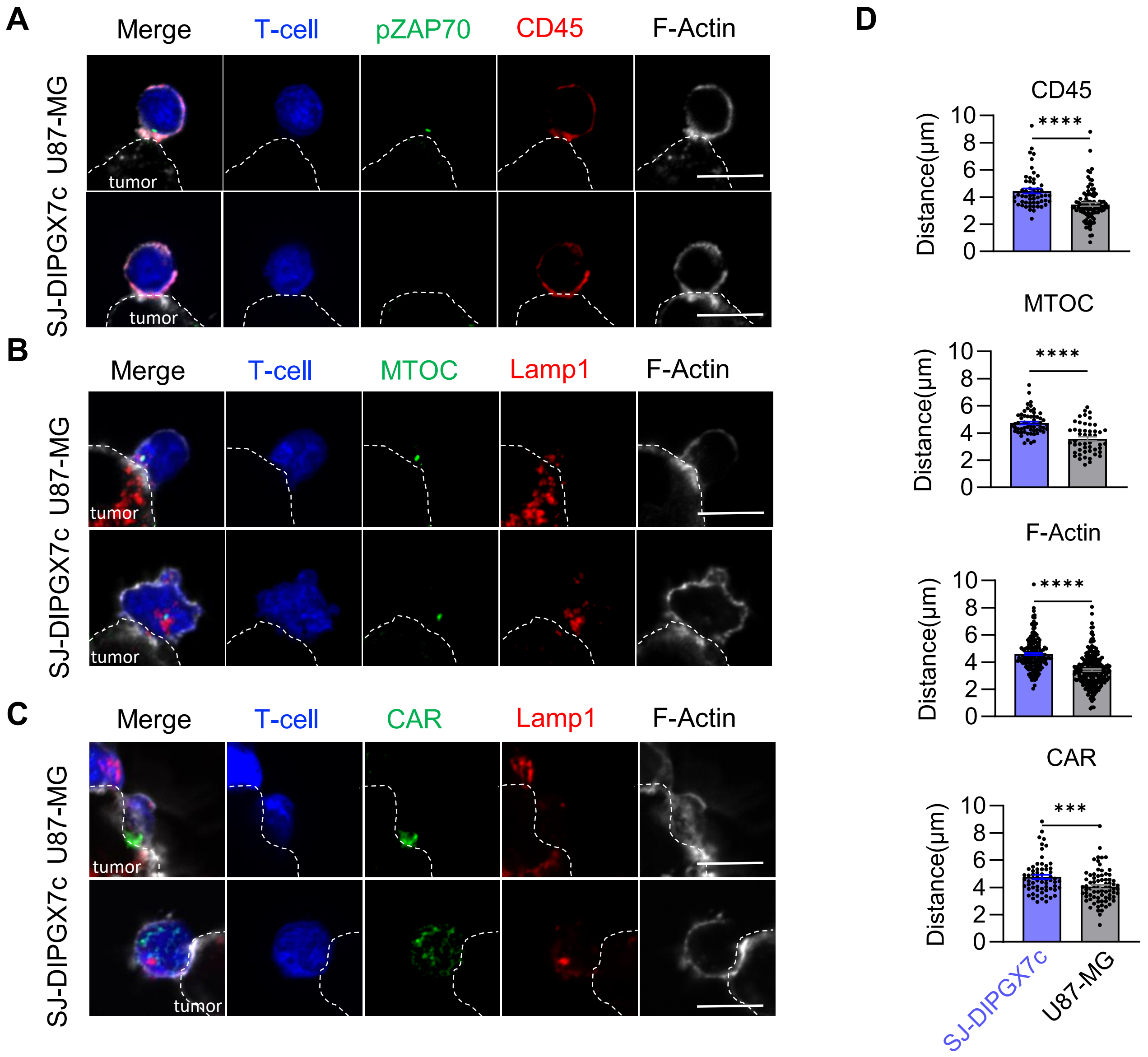

Fig. S5: Cell polarity is impaired during immune synapse formation in CAR T-cells interacting with DMG compared to GBM. (A), (B), and (C) Representative confocal images of CAR T-cells (blue) and tumor cells (SJ-DIPGX7c and U87-MG) interacting after 30 minutes of co-culture. pZAP70/MTOC/CAR (green), CD45/Lysosomes (Lamp1) (Red), and F-actin (gray), and merge are shown (scale bar=10 μm). (D) Quantification of the accumulation of CD45, Centrosome (MTOC), Cytoskeleton (F-Actin), and B7H3-CAR molecules at the immune synapse, measured as the distance of the center of mass of the labeling towards the synapse [smaller values indicate increased accumulation and polarization]. (N=2 T cell donors, cells=49-214 total, Unpaired t-test. ***p=0.0003, ****p<0.0001).
