# Supplementary material for: RASA2 deletion rescues immune synapse dysfunction, enhancing CAR T cell efficacy against DMGs
Source: J Immunother Cancer. 2026 Mar 30;14(3):e013134. doi: 10.1136/jitc-2025-013134 (PMC13052770; doi:10.1136/jitc-2025-013134)
Supplement: online supplemental figure 6 [file jitc-14-3-s006.pdf]

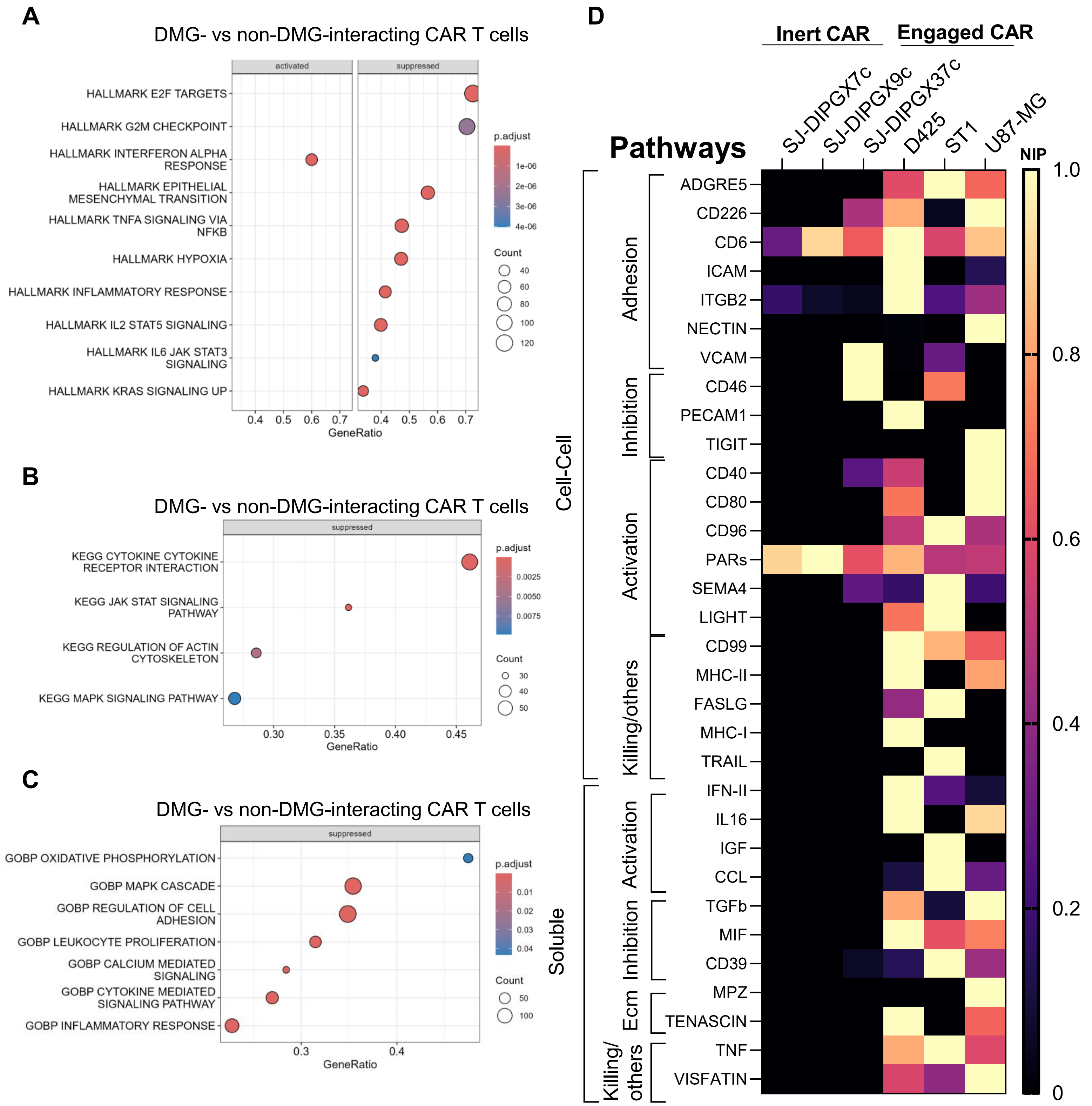

Fig. S6. CAR T-cells are inert to DMG interaction in different cell-to-cell communication pathways. (A), (B), and (C) GSEA enrichment plot of highly differentially regulated genes of DMG- vs. non-DMG-interacting CAR T-cells, using Hallmark, KEGG, and GO pathways databases, respectively. (D) Heatmap illustrating the normalized interaction probability (NIP) for each interaction pathway between CAR T-cells against DMG and non-DMG brain tumors, obtained by CellChat. CAR T-cells interacting with DMGs (SJ-DIPGX7c, SJ-DIPGX9c, and SJ-DIPGX37c) were classified as inert, whereas CAR T-cells interacting with non-DMG cells (D425, ST1, and U87-MG) were classified as engaged CAR T-cells.
