# Supplementary material for: RASA2 deletion rescues immune synapse dysfunction, enhancing CAR T cell efficacy against DMGs
Source: J Immunother Cancer. 2026 Mar 30;14(3):e013134. doi: 10.1136/jitc-2025-013134 (PMC13052770; doi:10.1136/jitc-2025-013134)
Supplement: online supplemental figure 7 [file jitc-14-3-s007.pdf]

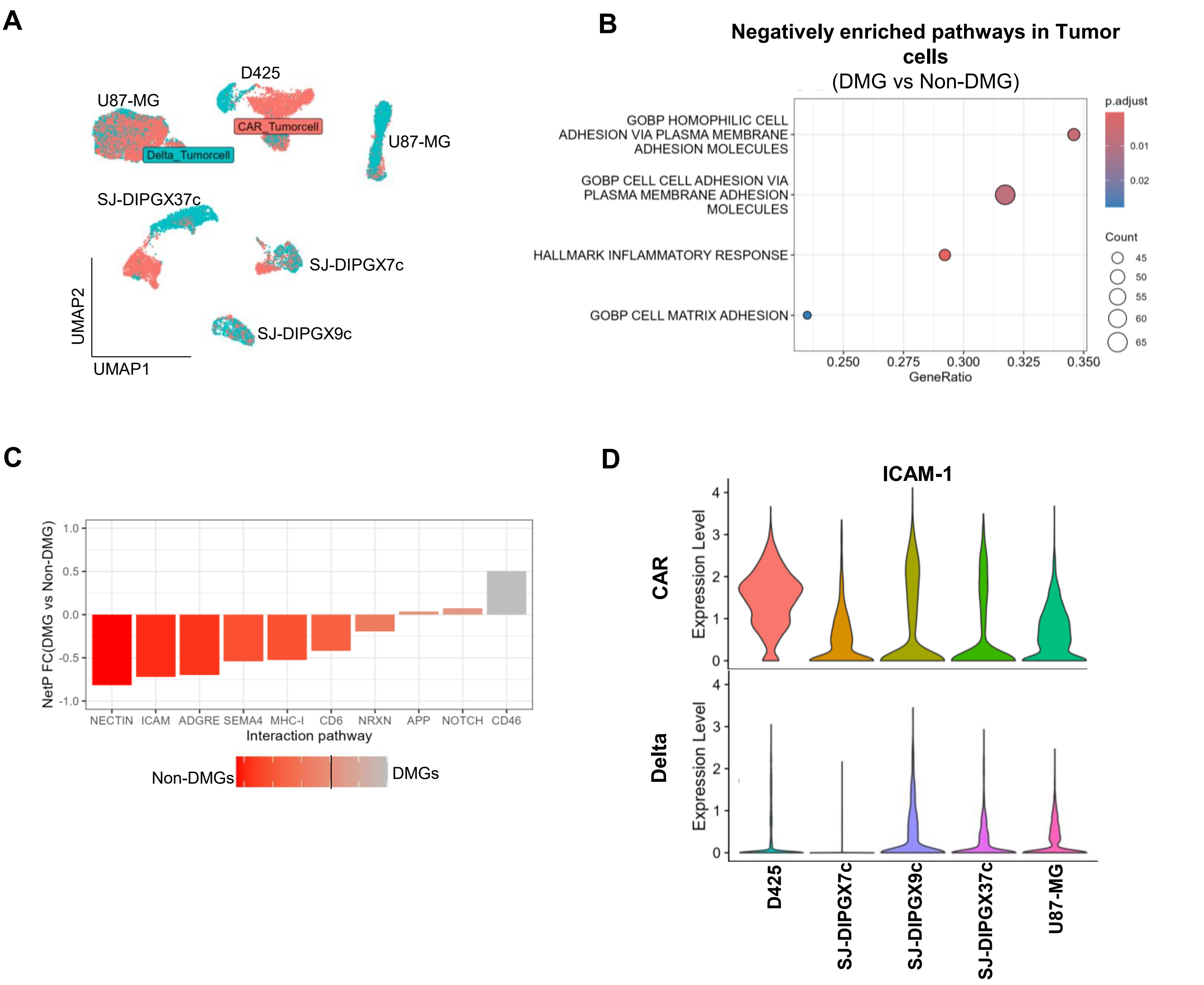

Fig. S7. DMG cells are unable to upregulate ICAM-1 at the same level of non-DMG tumor cells upon CAR T-cell interaction. (A) UMAP representation of the co-culture scRNA-seq data, depicting tumor cells in different co-culture conditions (CAR and Delta). (B) GSEA enrichment plot of highly differentially regulated genes of DMG- vs. non-DMG-tumor cells interacting with CAR T-cells. (C) Bar plot illustrating the fold change in outgoing signaling between DMG and non-DMG tumor cells interacting with CAR T-cells. (D) Violin plot illustrating ICAM-1 gene expression in tumor cells upon Delta or CAR T-cell co-culture.
