# Supplementary material for: RASA2 deletion rescues immune synapse dysfunction, enhancing CAR T cell efficacy against DMGs
Source: J Immunother Cancer. 2026 Mar 30;14(3):e013134. doi: 10.1136/jitc-2025-013134 (PMC13052770; doi:10.1136/jitc-2025-013134)
Supplement: online supplemental figure 8 [file jitc-14-3-s008.pdf]

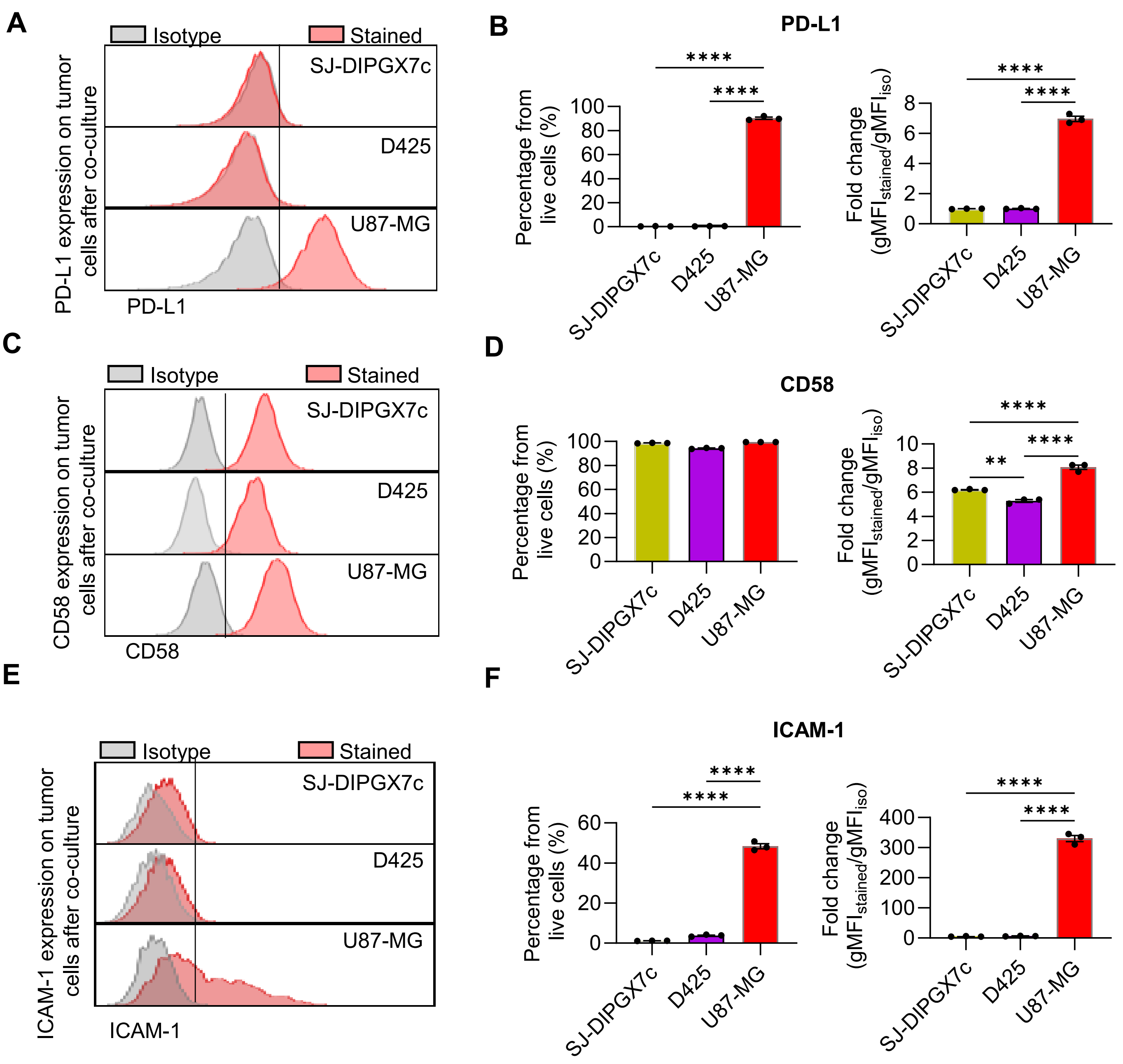

Fig. S8. PD-L1 and ICAM-1 protein levels are downregulated in DMGs. (A) and (B) Representative histogram and quantification of PD-L1 expression (Percentage and geometric mean fluorescence intensity [gMFI]) by flow cytometry on tumor cells (N=3 experimental replicates, One-way ANOVA, Tukey's multiple comparison test. ****$p<0.0001$). (C) and (D) Representative histogram and quantification of CD58 expression (Percentage and geometric mean fluorescence intensity [gMFI]) by flow cytometry on tumor cells (N=3 experimental replicates, One-way ANOVA, Tukey's multiple comparison test). (E) and (F) Representative histogram and quantification of ICAM-1 expression (Percentage and geometric mean fluorescence intensity [gMFI]) by flow cytometry on tumor cells (N=3 experimental replicates, One-way ANOVA, Tukey's multiple comparison test. *$p<0.05$ ****$p<0.0001$).
