# Supplementary material for: RASA2 deletion rescues immune synapse dysfunction, enhancing CAR T cell efficacy against DMGs
Source: J Immunother Cancer. 2026 Mar 30;14(3):e013134. doi: 10.1136/jitc-2025-013134 (PMC13052770; doi:10.1136/jitc-2025-013134)
Supplement: online supplemental figure 9 [file jitc-14-3-s009.pdf]

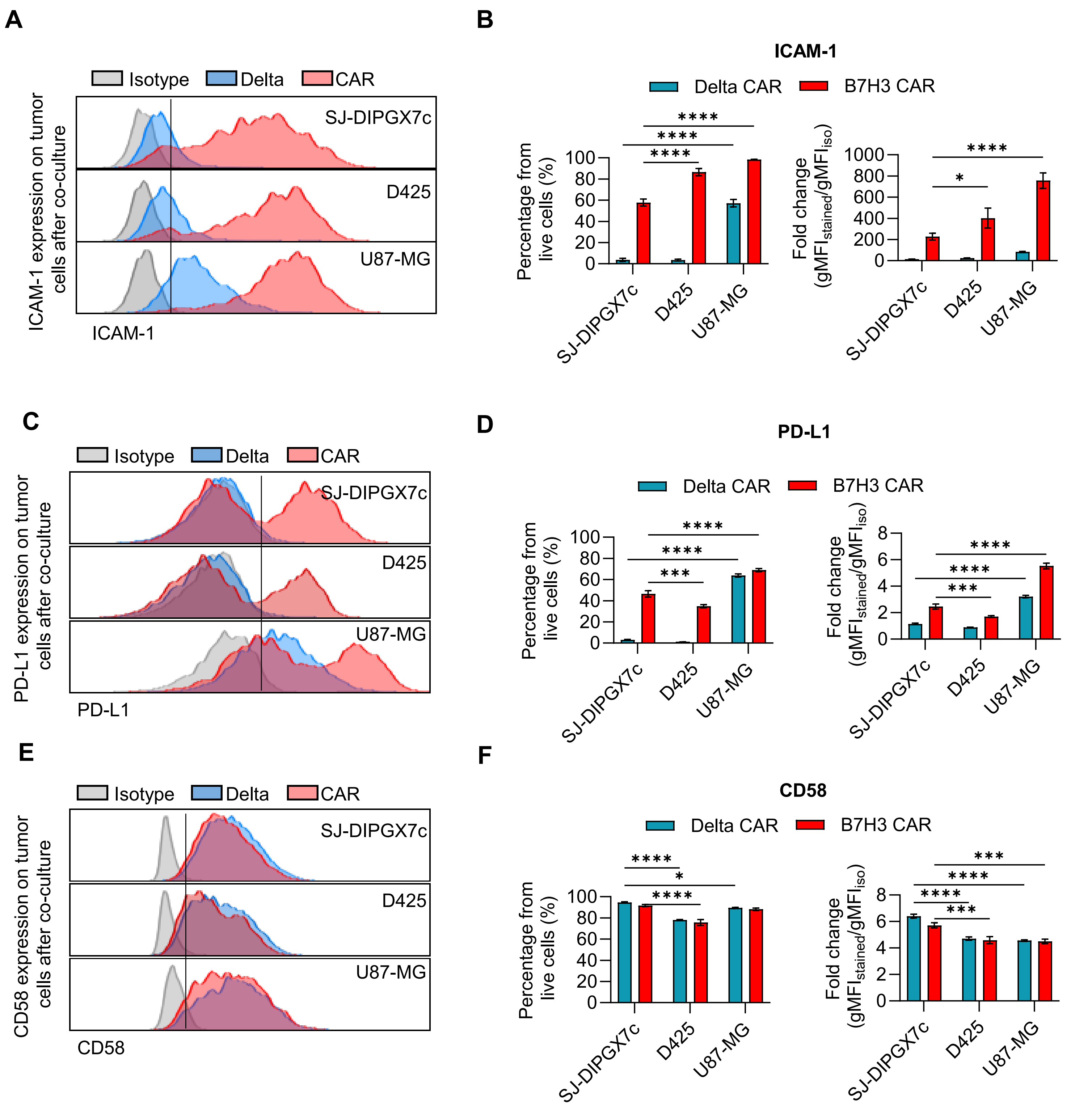


**Fig. S9. PD-L1, and CD58 do not play major role in CAR T-cell and tumor cell interaction when comparing DMG and non-DMG tumor cells.** (A) and (B) Representative histogram and quantification of ICAM-1 expression (Percentage and geometric mean fluorescence intensity [gMFI]) by flow cytometry on tumor cells after 24 hours of co-culture with T-cells (Delta and CAR expressing-cells) (N=3 T cell donors, Two-way ANOVA, uncorrected Fisher's LSD. *p<0.05, ****p<0.0001). (C) and (D) Representative histogram and quantification of PD-L1 expression (Percentage and geometric mean fluorescence intensity [gMFI]) by flow cytometry on tumor cells after 24 hours of coculture with T-cells (Delta and CAR expressing-cells) (N=3 T cell donors, Two-way ANOVA, uncorrected Fisher's LSD. ***p<0.001, ****p<0.0001). (E) and (F) Representative histogram and quantification of CD58 expression (Percentage and geometric mean fluorescence intensity [gMFI]) by flow cytometry on tumor cells after 24hrs of coculture with T cells (Delta and CAR expressing-cells) (N=3 T cell donors, Two-way ANOVA, uncorrected Fisher's LSD. *p<0.05, ****p<0.0001).
