# Supplementary material for: RASA2 deletion rescues immune synapse dysfunction, enhancing CAR T cell efficacy against DMGs
Source: J Immunother Cancer. 2026 Mar 30;14(3):e013134. doi: 10.1136/jitc-2025-013134 (PMC13052770; doi:10.1136/jitc-2025-013134)
Supplement: online supplemental figure 10 [file jitc-14-3-s010.pdf]

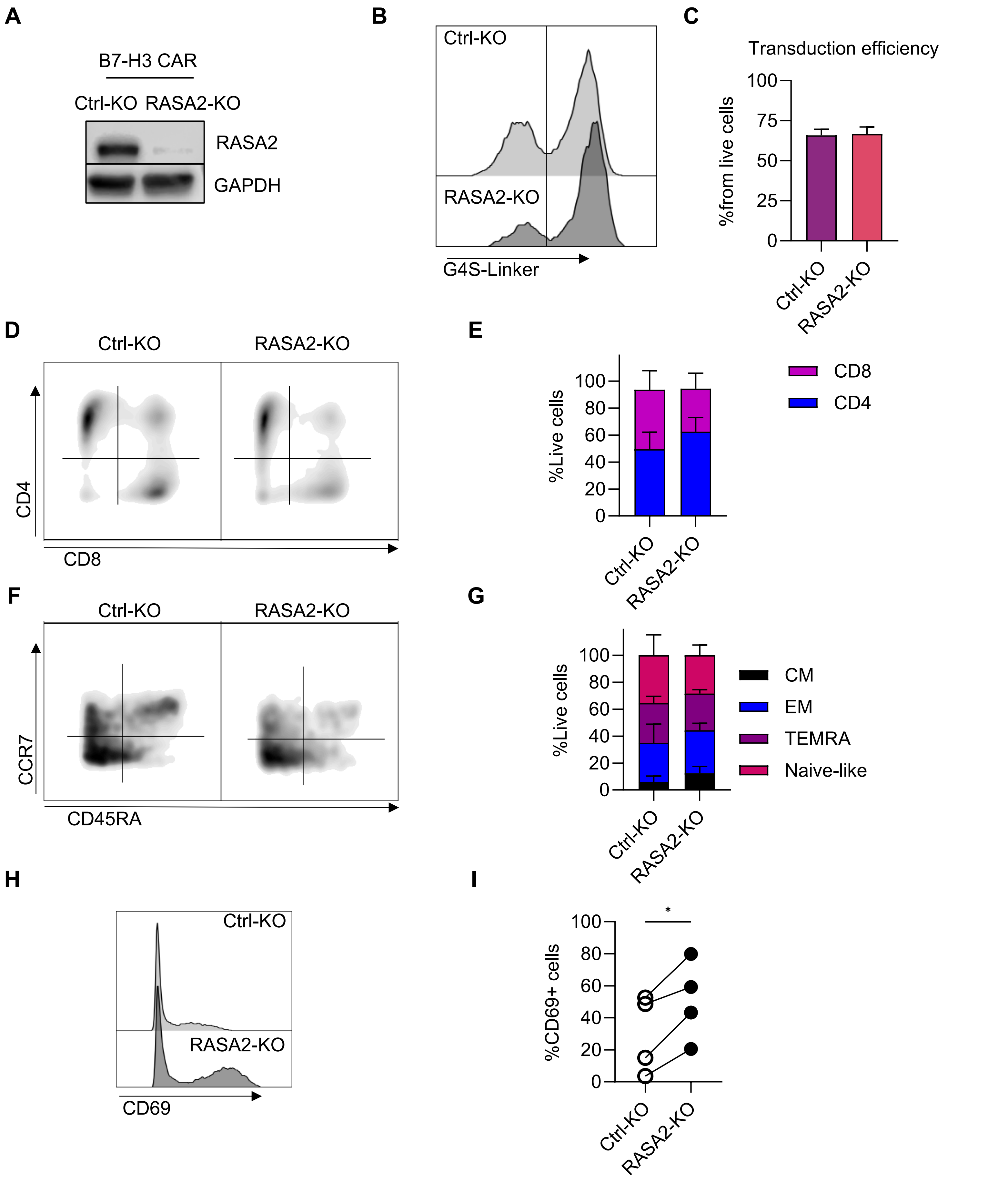
A
B7-H3 CAR
Ctrl-KO RASA2-KO
RASA2
GAPDH
B
Ctrl-KO
RASA2-KO
G4S-Linker
C
Transduction efficiency
%from live cells
100
75
50
25
0
Ctrl-KO
RASA2-KO
D
Ctrl-KO
RASA2-KO
CD4
CD8
E
%Live cells
100
80
60
40
20
0
CD8
CD4
Ctrl-KO
RASA2-KO
F
Ctrl-KO
RASA2-KO
CCR7
CD45RA
G
%Live cells
100
80
60
40
20
0
CM
EM
TEMRA
Naive-like
Ctrl-KO
RASA2-KO
H
Ctrl-KO
RASA2-KO
CD69
I
*
%CD69+ cells
100
80
60
40
20
0
Ctrl-KO
RASA2-KO

Fig. S10. RASA2-KO does not alter CAR transduction or memory phenotype but increases CD69 surface expression in T-cells. (A) Representative immunoblot of RASA2-KO efficiency. (B) Representative histogram plot CAR expression (G4S-Linker) on Ctrl- and RASA2-KO T-cells after 5 days of transduction, measured by flow cytometry. (C) Quantification of transduction efficiency showed in (B) (N=5 T cell donors). (D) Representative density plots of CD4 and CD8 expression on T-cells after transduction and gene KO, measured by flow cytometry. (E) Quantification of CD4 and CD8 proportion in (D) (N=3 T cell donors). (F) Representative density plots of CD8 memory phenotype on T-cells after transduction and gene KO, measured by flow cytometry. (E) Quantification of CD8 memory phenotype in (F): Central Memory (CM; CCR7+CD45RA-), Effector memory (EM;CCR7-CD45RA-), Terminally differentiated (TEMRA; CCR7-CD45RA+), and Naïve like (CCR7+CD45RA+) (N=3). (H) Representative histogram plot of CD69 expression on T-cells transduction and gene KO. (**I**) CD69 expression quantification in (H) (N=4 T cell donors, paired t-test. *p= 0.164).
