# Supplementary material for: RASA2 deletion rescues immune synapse dysfunction, enhancing CAR T cell efficacy against DMGs
Source: J Immunother Cancer. 2026 Mar 30;14(3):e013134. doi: 10.1136/jitc-2025-013134 (PMC13052770; doi:10.1136/jitc-2025-013134)
Supplement: online supplemental figure 12 [file jitc-14-3-s012.pdf]

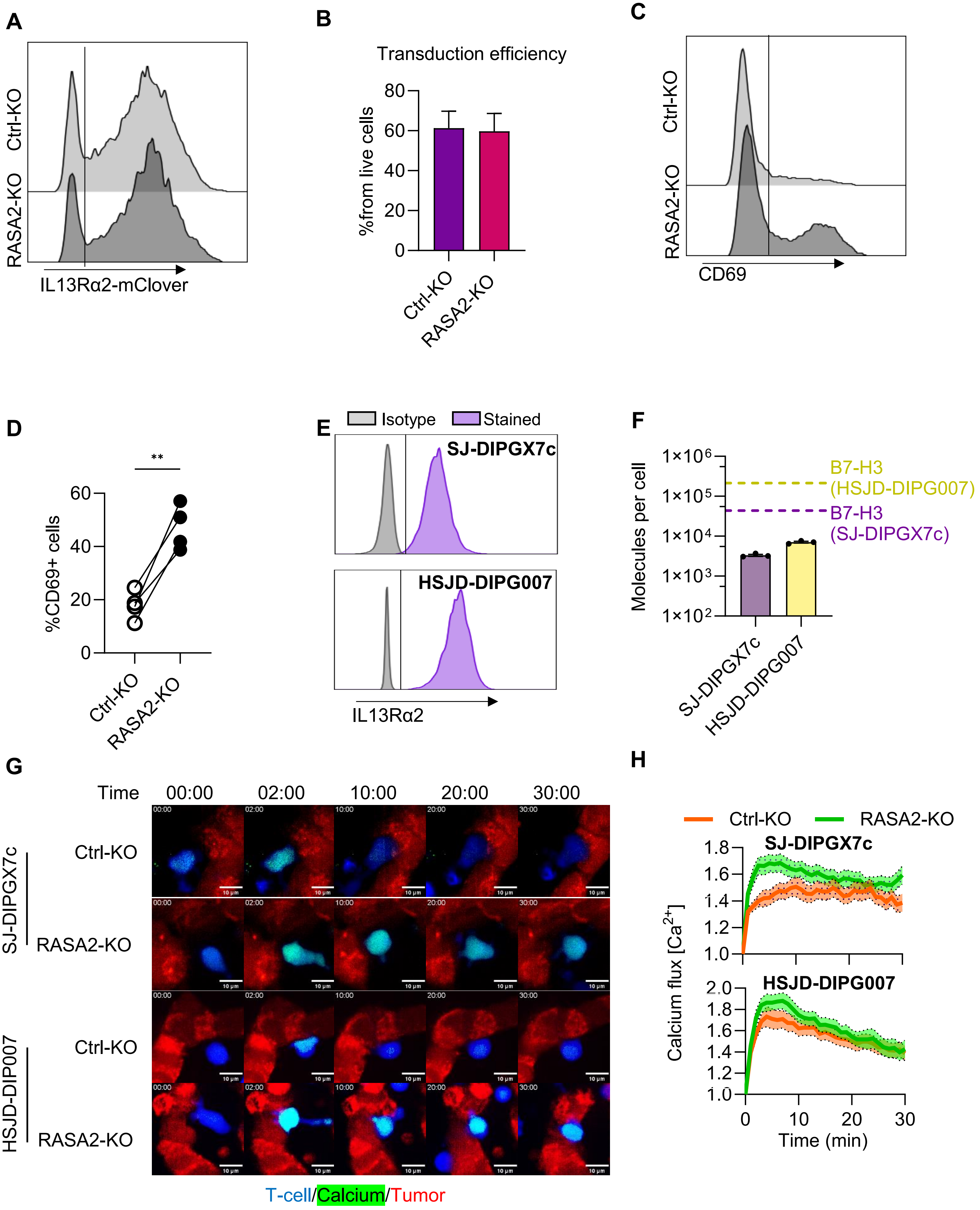
A
Ctrl-KO
RASA2-KO
IL13Rα2-mClover
B
Transduction efficiency
%from live cells
0
20
40
60
80
Ctrl-KO
RASA2-KO
C
Ctrl-KO
RASA2-KO
CD69
D
**
%CD69+ cells
0
20
40
60
Ctrl-KO
RASA2-KO
E
Isotype
Stained
SJ-DIPGX7c
HSJD-DIPG007
IL13Rα2
F
Molecules per cell
1×10^2
1×10^3
1×10^4
1×10^5
1×10^6
B7-H3 (HSJD-DIPG007)
B7-H3 (SJ-DIPGX7c)
SJ-DIPGX7c
HSJD-DIPG007
G
Time
00:00
02:00
10:00
20:00
30:00
SJ-DIPGX7c
Ctrl-KO
RASA2-KO
HSJD-DIP007
Ctrl-KO
RASA2-KO
T-cell/Calcium/Tumor
H
Ctrl-KO
RASA2-KO
SJ-DIPGX7c
HSJD-DIPG007
Calcium flux [Ca2+]
1.0
1.2
1.4
1.6
1.8
2.0
0
10
20
30
Time (min)

Fig. S12. RASA2-KO does not alter IL13Rα2 CAR transduction but increases CD69 surface expression in T-cells and Calcium flux in IL13Rα2 –specific CAR T-cells upon DMG interaction. (A) Representative histogram plot of CAR expression on T-cells after 5 days of transduction, measured by mClover expression by flow cytometry. (B) Quantification of transduction efficiency showed in (A) (N=4 T cell donors). (C) and (D) Representative histogram plot of CD69 expression on T-cells after transduction and gene KO, and its respective quantification (N=4 T cell donors, paired t-test, **p= 0.0058). (E) Representative histogram plots of IL13Rα2 expression in SJ-DIPGX7c and HSJD-DIPG007. (F) IL13Rα2 molecules per cell quantification in SJ-DIPGX7c and HSJD-DIPG007. B7-H3 molecules per cell levels are shown as dashed lines for both DMG tumor cells. (G) Representative confocal live cell time-lapse images of RASA2-KO or Ctrl-KO CAR T-cells interacting with different DMGs. CAR T-cells were labeled with CellTrace violet (Blue) and CAL590AM (Green) for calcium flux, and tumor cells were labeled with CellTrace Far red (Red) (scale bar=10μm). (H) Quantification of calcium flux in CAR T-cells upon tumor cell interaction shown in (G).
