# Supplementary material for: RASA2 deletion rescues immune synapse dysfunction, enhancing CAR T cell efficacy against DMGs
Source: J Immunother Cancer. 2026 Mar 30;14(3):e013134. doi: 10.1136/jitc-2025-013134 (PMC13052770; doi:10.1136/jitc-2025-013134)
Supplement: online supplemental figure 13 [file jitc-14-3-s013.pdf]

Fig. S13

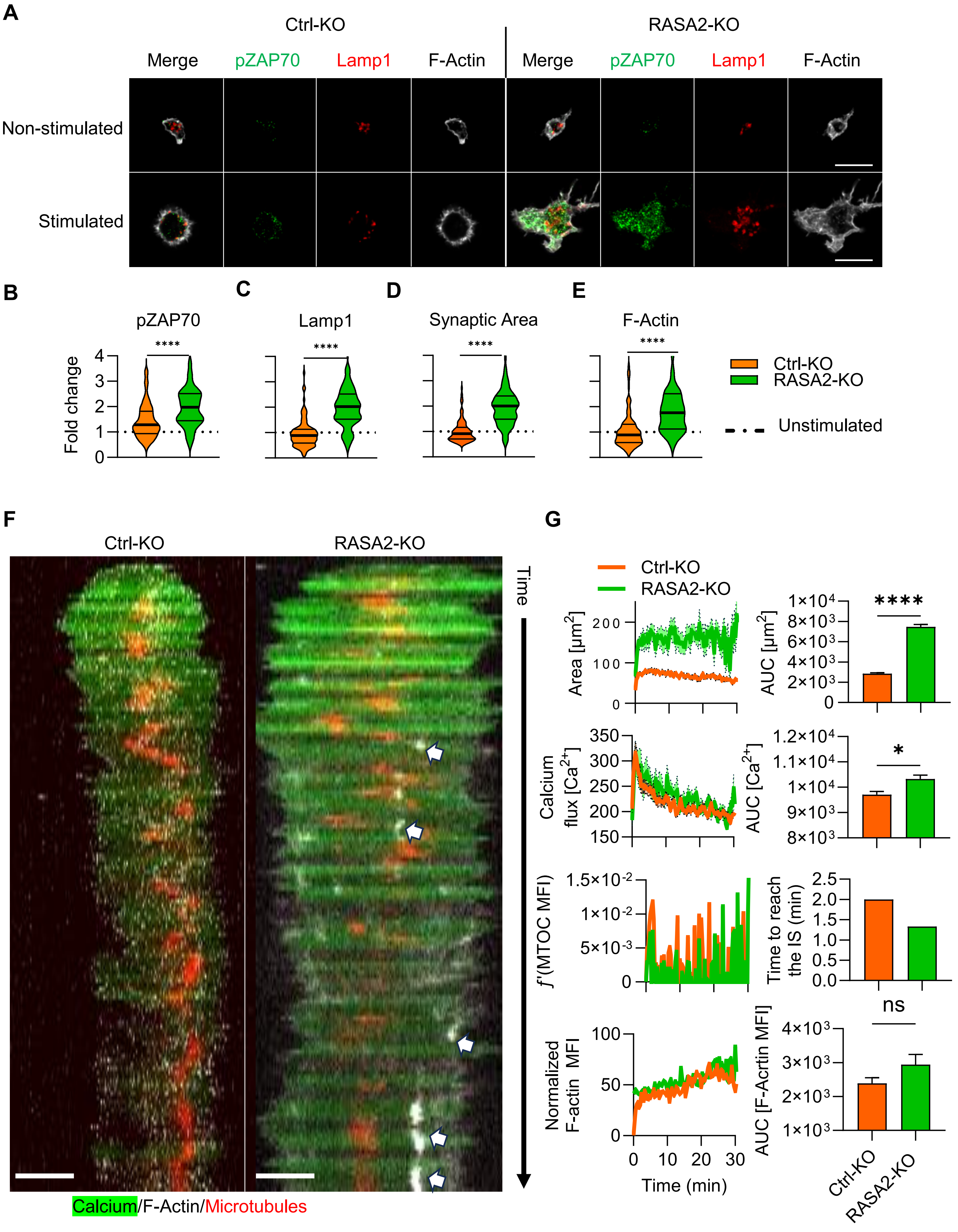
A
Ctrl-KO
RASA2-KO
Merge
pZAP70
Lamp1
F-Actin
Non-stimulated
Stimulated
B
pZAP70
Fold change
C
Lamp1
D
Synaptic Area
E
F-Actin
Ctrl-KO
RASA2-KO
Unstimulated
F
Ctrl-KO
RASA2-KO
Time
Calcium/F-Actin/Microtubules
G
Ctrl-KO
RASA2-KO
Area [µm²]
AUC [µm²]
Calcium flux [Ca²⁺]
AUC [Ca²⁺]
f'(MTOC MFI)
Time to reach the IS (min)
Normalized F-actin MFI
AUC [F-Acrtin MFI]
ns
Time (min)

Fig. S13. RASA2-KO increases the immune synapse quality in CAR T-cells upon CAR or CD3/CD28 activation. (A) Representative confocal images of CAR T-cells (blue) activated onto B7-H3 coated-cover glasses for 30 minutes. pZAP70 (green), Lysosomes (Lamp1) (Red), and F-actin (gray), merge are shown (scale bar=10μm). (B-E) Quantification of the accumulation of pZAP70, Lysosomes (Lamp1), Synapse area, and Actin cytoskeleton (F-Actin) at the immune synapse, measured as the fluorescence accumulation at the synapse normalized by unstimulated control. respectively (N=78-94 total cells analyzed, Unpaired t-test. ****p<0.0001). (F) Representative time-lapse kymograph (x-axis represents synapse size and y-axis represents time) of T-cells activated onto CD3/CD28 coated surfaces, imaged by TIRFM (scale bar=10μm). White arrows represent actin foci formation. (G) Quantification of the synaptic size, calcium flux, MTOC recruitment to the IS (measured as the first derivative of MTOC MFI), and F-actin accumulation at the IS (measured as F-Actin MFI) (N=11-29 total cells analyzed, unpaired t-test. *p=0.0113, ****p<0.0001).
