# Supplementary material for: RASA2 deletion rescues immune synapse dysfunction, enhancing CAR T cell efficacy against DMGs
Source: J Immunother Cancer. 2026 Mar 30;14(3):e013134. doi: 10.1136/jitc-2025-013134 (PMC13052770; doi:10.1136/jitc-2025-013134)
Supplement: online supplemental figure 14 [file jitc-14-3-s014.pdf]

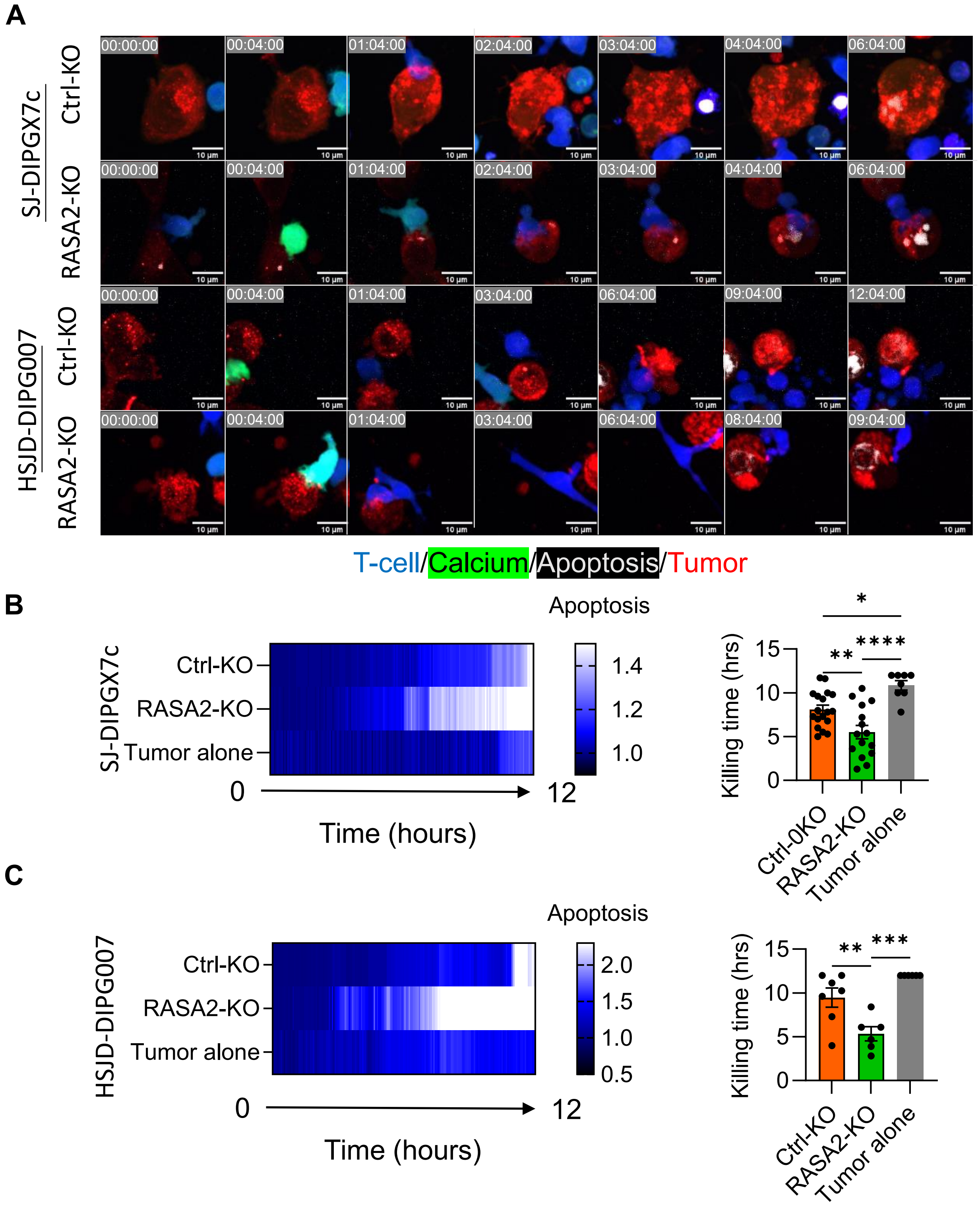

Fig. S14. RASA2-KO increases the killing speed of CAR T-cells against DMGs. (A) Representative time-lapse images of CAR T-cells (Ctrl-KO and RASA2-KO) interacting with tumor cells (SJ-DIPGX7c and HSJD-DIPG007) acquired by confocal live cell imaging. CAR T-cells were labeled with CellTrace violet (Blue) and CAL520 (Green), tumor cells were labeled with CellTracker Red-CMTPX (Red), Apoptosis was measured as the internalization of DRAQ7 into the tumor cell nucleus (white) (scale bar=10 µm). (B) and (C) Quantification of DRAQ7 MFI in tumor cells normalized by its MFI at the T-cell interaction timepoint shown in (A). Tumor alone condition quantification, and the killing time upon single T-cell:tumor cell interaction are also shown (N=4-7 total cells analyzed, Unpaired t-test. *p<0.05, **p<0.01, ***p<0.001).
