# Supplementary material for: RASA2 deletion rescues immune synapse dysfunction, enhancing CAR T cell efficacy against DMGs
Source: J Immunother Cancer. 2026 Mar 30;14(3):e013134. doi: 10.1136/jitc-2025-013134 (PMC13052770; doi:10.1136/jitc-2025-013134)
Supplement: online supplemental figure 15 [file jitc-14-3-s015.pdf]

Fig. S15

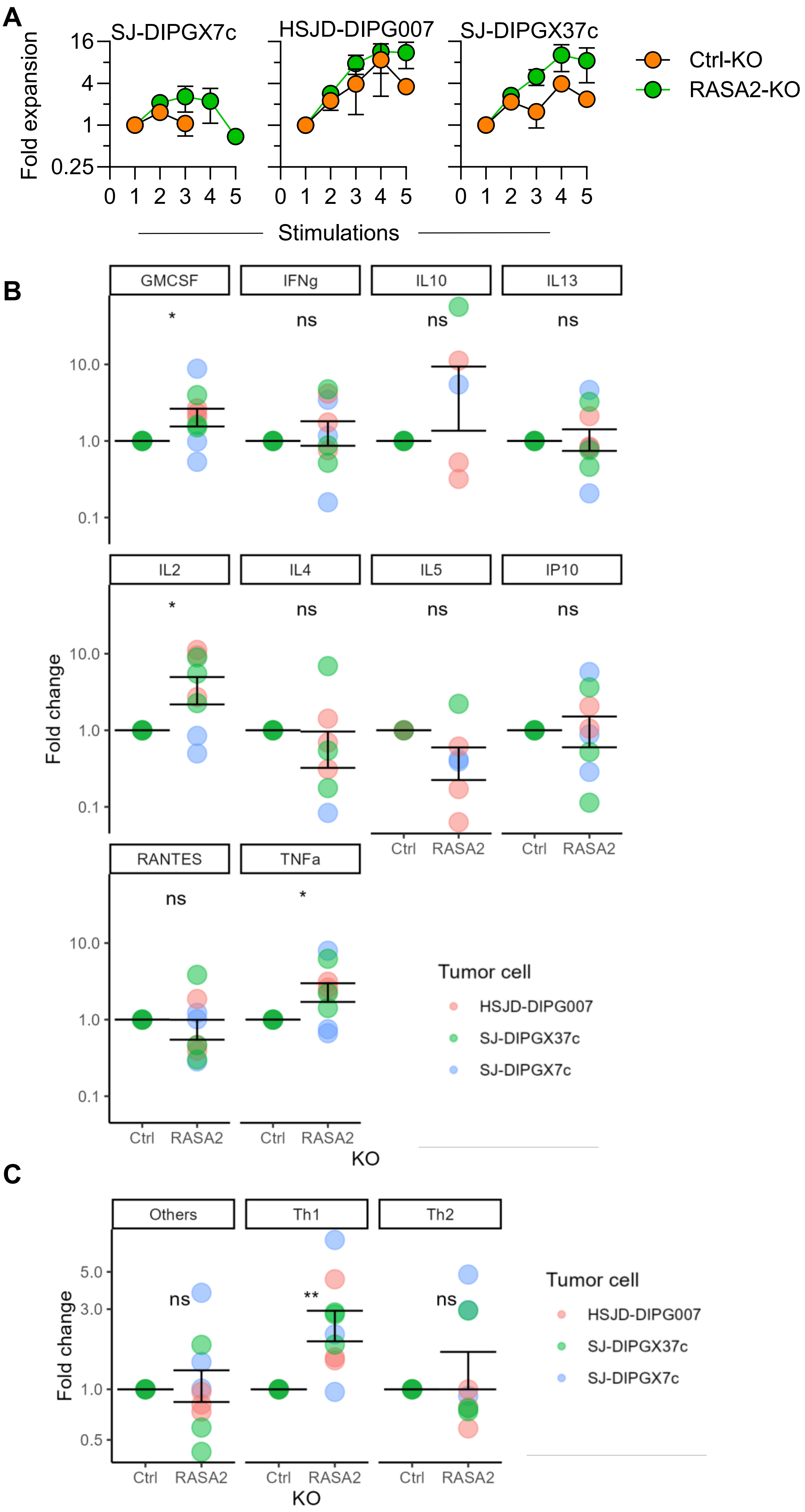
A
SJ-DIPGX7c
HSJD-DIPG007
SJ-DIPGX37c
Fold expansion
16
4
1
0.25
0 1 2 3 4 5
Stimulations
Ctrl-KO
RASA2-KO
B
GMCSF
IFNg
IL10
IL13
IL2
IL4
IL5
IP10
RANTES
TNFa
*
ns
10.0
1.0
0.1
Fold change
Ctrl
RASA2
KO
Tumor cell
HSJD-DIPG007
SJ-DIPGX37c
SJ-DIPGX7c
C
Others
Th1
Th2
**
5.0
3.0
1.0
0.5

Fig. S15. RASA2-KO increases cytokine secretion but not persistence of B7H3 CAR T-cells against DMGs. (A) Serial stimulation assay using effector T-cells (B7-H3 Ctrl-KO or RASA2-KO T-cells) against SJ-DIPGX7c, HSJD-DIPG007, and SJ-DIPGX37c (N=5 T cell donors), target cells at 2:1 E:T ratio. Fresh target cells were added every 7 days. (B) Summary plots of cytokine production (GM-CSF, IFN-γ, TNF-α, IL-2, IL-4, IL-5, IL-10, IL-13, RANTES, and IP-10) by B7-H3-CAR T (Ctrl-KO or RASA2-KO) in the supernatant when cultured with SJ-DIPGX7c (N=3 T cell donors), HSJD-DIPG007 (N=3 T cell donors), and SJ-DIPGX37c (N=3 T cell donors) at a 2:1 E:T ratio after 24 hours of stimulation. Cytokines were measured using MILLIPLEX® cytokine assay (Paired t-test, *p<0.05). (C) Cytokine secretion aggregation by response type. Th1: GM-CSF, IFN-γ, TNF-α, IL-2; Th2: IL-4, IL-5, IL-10, IL-13; and Others: RANTES and IP-10 in B7-H3 RASA2-KO CAR T-cells, normalized by Ctrl-KO CAR T-cells against SJ-DIPGX7c, HSJD-DIPG007, and SJ-DIPGX37c. (N=3 per tumor type, paired t-test, *p<0.05).
