# Supplementary material for: RASA2 deletion rescues immune synapse dysfunction, enhancing CAR T cell efficacy against DMGs
Source: J Immunother Cancer. 2026 Mar 30;14(3):e013134. doi: 10.1136/jitc-2025-013134 (PMC13052770; doi:10.1136/jitc-2025-013134)
Supplement: online supplemental figure 16 [file jitc-14-3-s016.pdf]

A

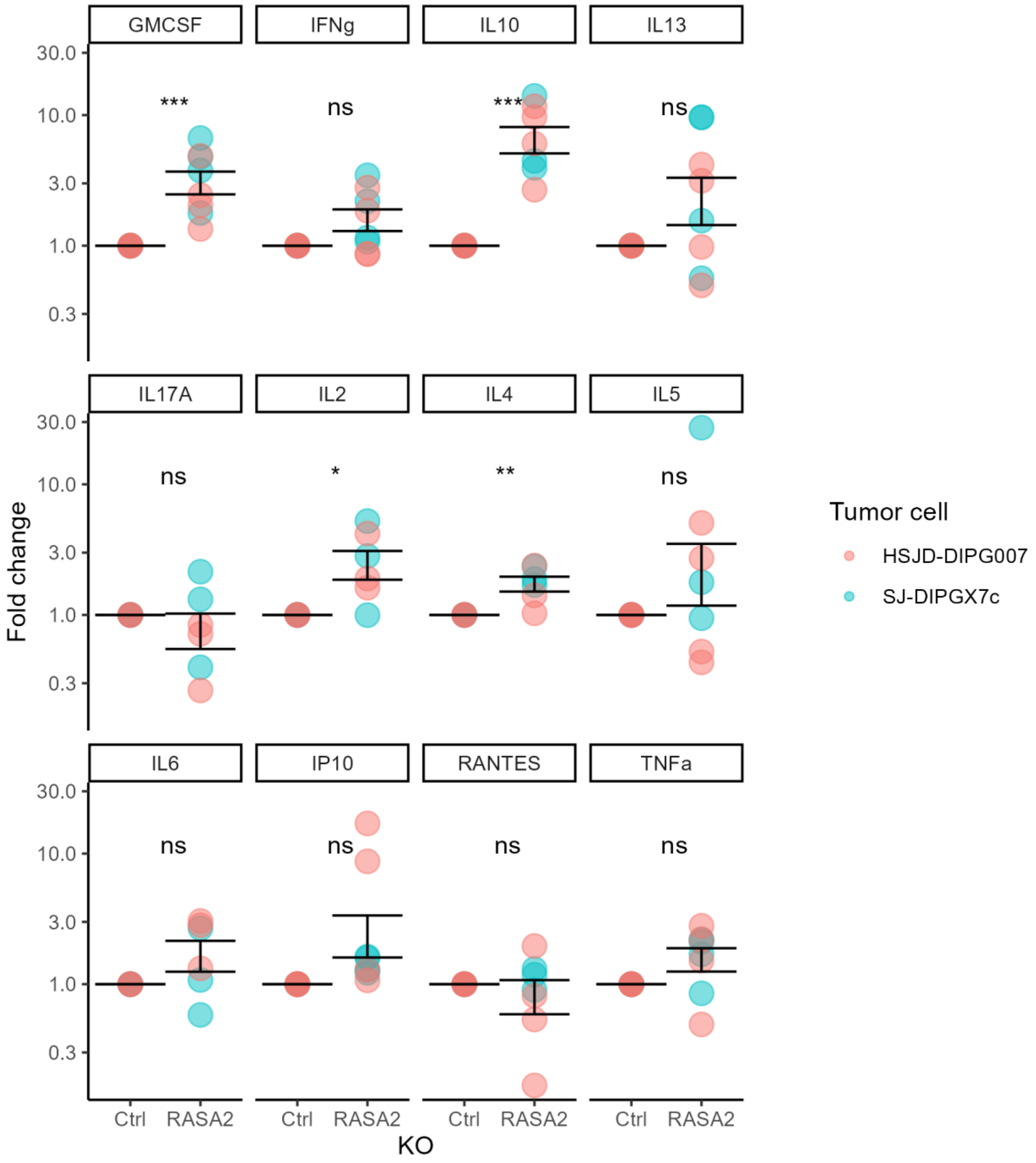
GMCSF
IFNg
IL10
IL13
IL17A
IL2
IL4
IL5
IL6
IP10
RANTES
TNFa
***
ns
***
ns
ns
*
**
ns
ns
ns
ns
ns
30.0
10.0
3.0
1.0
0.3
Fold change
Ctrl
RASA2
KO
Tumor cell
HSJD-DIPG007
SJ-DIPGX7c

B

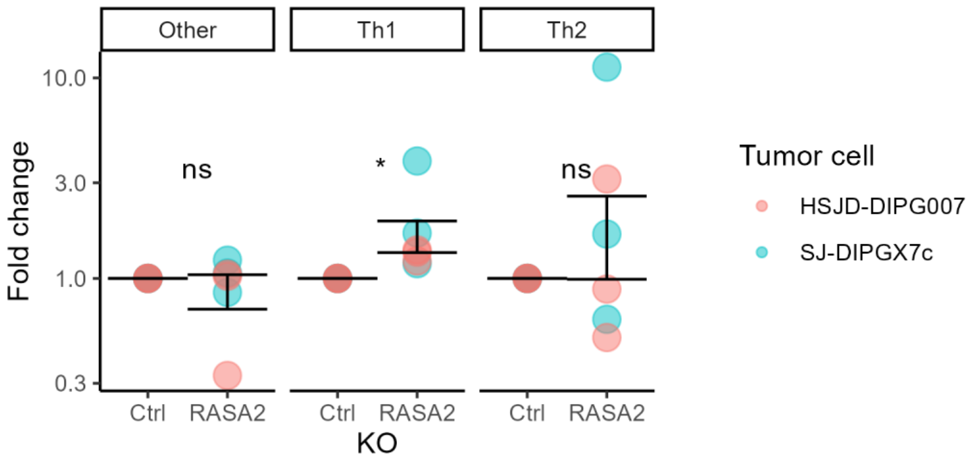
Other
Th1
Th2
ns
*
ns
10.0
3.0
1.0
0.3
Fold change
Ctrl
RASA2
KO
Tumor cell
HSJD-DIPG007
SJ-DIPGX7c

**Fig. S16. RASA2-KO increases CAR T-cell cytokine secretion in IL13Rα2 CAR T-cells against DMGs.** (A) Summary plots of cytokine production (GM-CSF, IFN-γ, TNF-α, IL-2, IL-4, IL-5, IL-10, IL-13, IL17A, RANTES, and IP-10) by IL13Rα2-CAR T-cells (Ctrl-KO or RASA2-KO) in the supernatant when cultured with SJ-DIPGX7c (N=4 T cell donors), and HSJD-DIPG007 (N=4 T cell donors) cells at 2:1 E:T ratio after 24 hours of stimulation. Cytokines were measured by using MILLIPLEX® cytokine assay. (Paired t-test. *$p<0.05$, **$p<0.01$). (B) Cytokine secretion aggregation by response type. Th1: GM-CSF, IFN-γ, TNF-α, IL-2; Th2: IL-4, IL-5, IL-10, IL-13; and Others: RANTES, IL17A, and IP-10 in B7-H3 RASA2-KO IL13Rα2-CAR T-cells , normalized by Ctrl-KO IL13Rα2-CAR T-cells against SJ-DIPGX7c, and HSJD-DIPG007. (N=4 T cell donors per tumor type, paired t-test. *$p<0.05$).
