# Supplementary material for: RASA2 deletion rescues immune synapse dysfunction, enhancing CAR T cell efficacy against DMGs
Source: J Immunother Cancer. 2026 Mar 30;14(3):e013134. doi: 10.1136/jitc-2025-013134 (PMC13052770; doi:10.1136/jitc-2025-013134)
Supplement: online supplemental figure 17 [file jitc-14-3-s017.pdf]

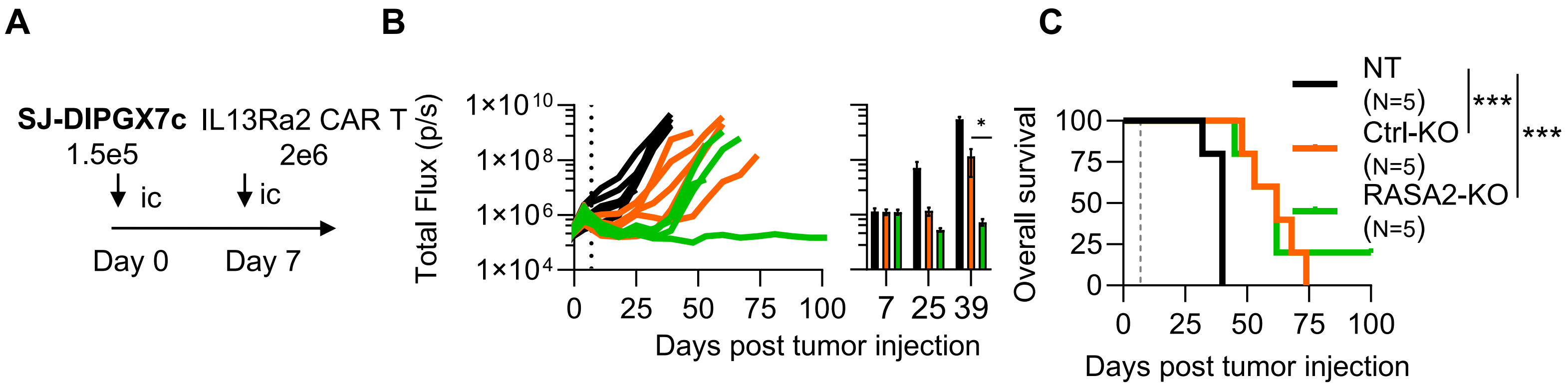

Fig. S17. RASA2-KO improves IL13Rα2 CAR T-cells early *in vivo* tumor burden control against SJ-DIPGX7c. (A) Schematic of the *in vivo* experimental design for tumor intracranial implantation model. Tumor cells were implanted intracranially (ic) into the brain cortex, followed by a single ic dose of $2x10^6$ IL13Rα2 (Ctrl- and RASA2-KO) or non-transduced (NT) T-cells post tumor injection. (B) Total flux from tumor cells in all mice treated with CAR T-cells. The tumors were measured weekly using bioluminescence imaging (Multiple unpaired t-test, *$p<0.05$). (C) Kaplan-Meier survival analysis of mice treated with CAR T-cells log rank (Mantel-Cox) test, N=5 mice per group, ***$p<0.001$.
