# Supplementary material for: RASA2 deletion rescues immune synapse dysfunction, enhancing CAR T cell efficacy against DMGs
Source: J Immunother Cancer. 2026 Mar 30;14(3):e013134. doi: 10.1136/jitc-2025-013134 (PMC13052770; doi:10.1136/jitc-2025-013134)
Supplement: online supplemental figure 18 [file jitc-14-3-s018.pdf]

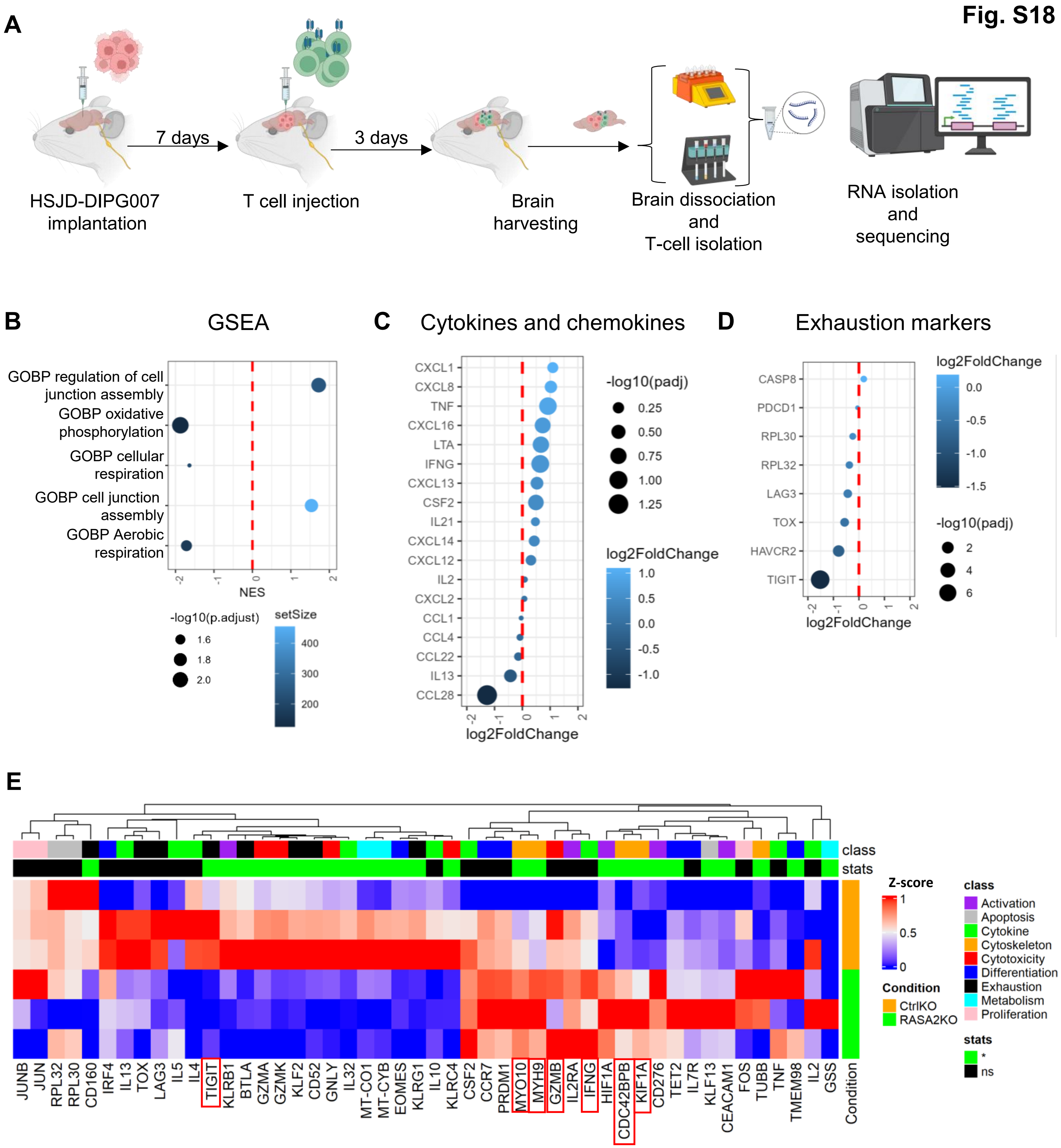

Fig. S18. RASA2 KO increases T-cell effector and cell adhesion signatures *in vivo* against DMG. (A) Schematic of CAR T-cell (RASA2- and Ctrl-KO B7-H3-CAR expressing cells) isolation from brain tumors (HSJD-DIPG007) and total RNA sequencing from purified RNA. CAR T-cells were harvested from 3 mice per group. (B) GSEA results using the gene ontology pathways database and comparing the gene expression of RASA2- and Ctrl-KO CAR T-cells from (A). (C) and (D) Dotplot illustrating the differential gene expression between RASA2 and Ctrl-KO CAR T cells for genes associated with cytokine and chemokine expression and exhaustion, respectively. (E) Heatmap representing the most differently regulated genes when comparing RASA2- and Ctrl-KO CAR T-cells from (A), depicting each individual replicate, the functional group for each gene (class) and their respective statistical significance (stats).
