# Supplementary material for: RASA2 deletion rescues immune synapse dysfunction, enhancing CAR T cell efficacy against DMGs
Source: J Immunother Cancer. 2026 Mar 30;14(3):e013134. doi: 10.1136/jitc-2025-013134 (PMC13052770; doi:10.1136/jitc-2025-013134)
Supplement: online supplemental figure 19 [file jitc-14-3-s019.pdf]

Fig. S19

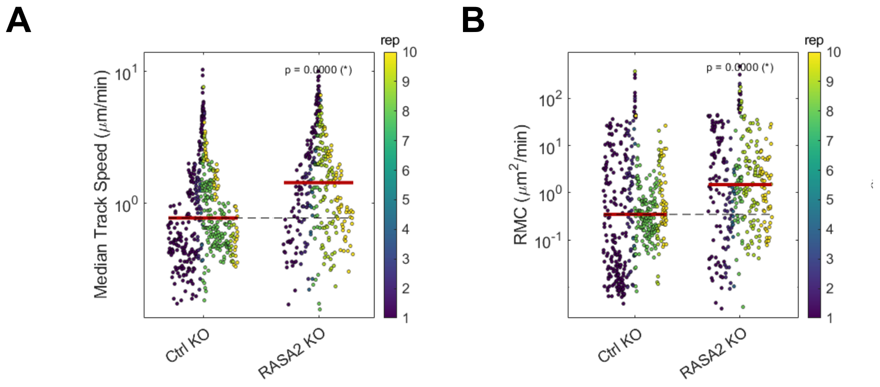

Fig. S19. Extended T cell migration analysis in brain slices. (A) Overall Median track speed of Ctrl- and RASA2-KO CAR T cells in brain slices. (B) Random motility coefficient (RMC) (N=3, cells=67-238, Kruskal-Wallis test, *$p<0.05$)
