# Supplementary material for: RASA2 deletion rescues immune synapse dysfunction, enhancing CAR T cell efficacy against DMGs
Source: J Immunother Cancer. 2026 Mar 30;14(3):e013134. doi: 10.1136/jitc-2025-013134 (PMC13052770; doi:10.1136/jitc-2025-013134)
Supplement: online supplemental figure 20 [file jitc-14-3-s020.pdf]

Fig. S20

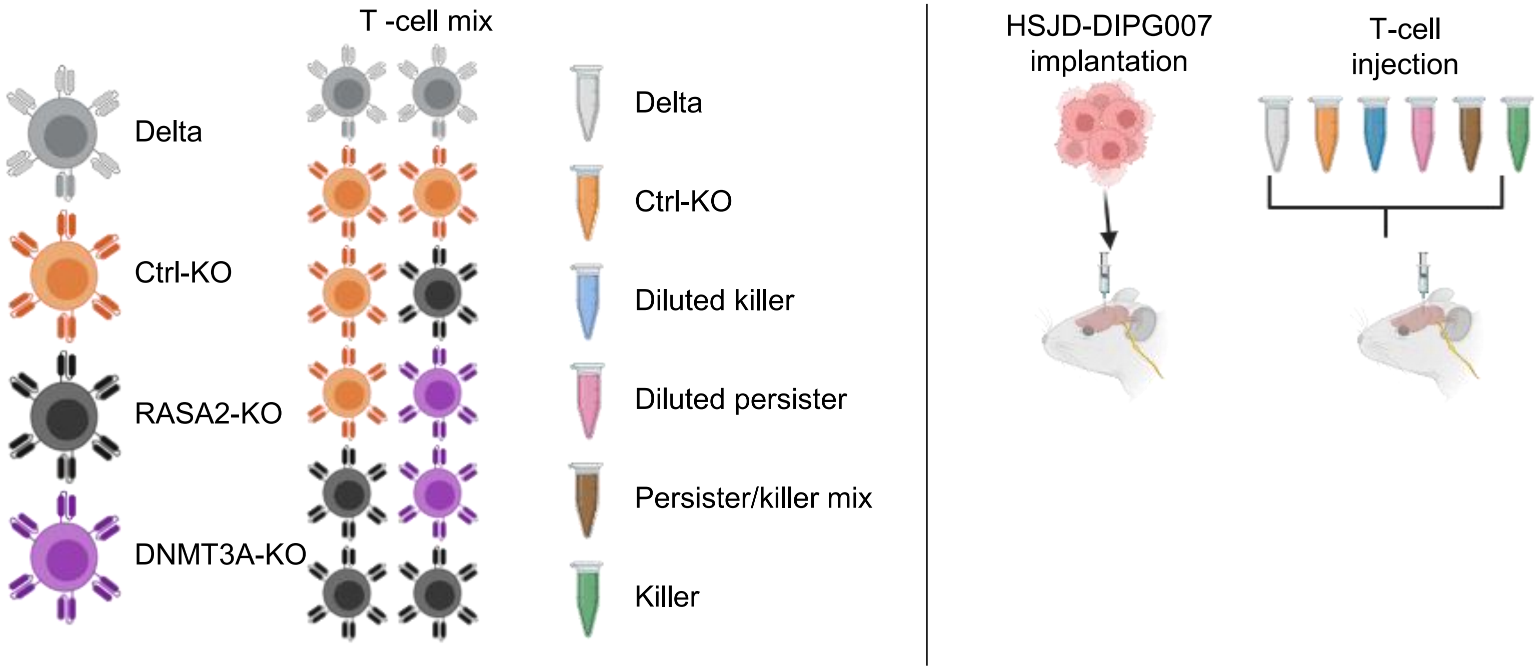

Fig. S20. Experimental schematic for CAR T-cell knockout mixture in DMG model. Schematic representation of CAR T-cell Knockout mix and *in vivo* experiment setup. All mixtures were 1:1 ratio between CAR T-cell products (Ctrl-KO, RASA2-KO, and DNMT3A-KO).
